# tRF-His-GTG-1 enhances NETs formation and interferon-α production in lupus by extracellular vesicle

**DOI:** 10.1186/s12964-024-01730-7

**Published:** 2024-07-07

**Authors:** Yi-Ming Chen, Kuo-Tung Tang, Hung-Jen Liu, Shih-Ting Huang, Tsai-Ling Liao

**Affiliations:** 1 Department of Medical Research, Taichung Veterans General Hospital, No.1650, Sec.4, Taiwan Boulevard, Xitun Dist, Taichung, 40705 Taiwan; 2 Rong Hsing Research Center for Translational Medicine, National Chung Hsing University, Taichung, 40227 Taiwan; 3 Ph.D. Program in Translational Medicine, National Chung Hsing University, Taichung, 40227 Taiwan; 4 Division of Allergy, Immunology and Rheumatology, Department of Internal Medicine, Taichung Veterans General Hospital, Taichung, 40705 Taiwan; 5 Institute of Molecular Biology, National Chung Hsing University, Taichung, 40227 Taiwan; 6 The iEGG and Animal Biotechnology Center, National Chung Hsing University, Taichung, 40227 Taiwan; 7 Division of Nephrology, Department of Internal Medicine, Taichung Veterans General Hospital, Taichung, 40705 Taiwan; 8 Department of Post-Baccalaureate Medicine, College of Medicine, National Chung Hsing University, Taichung, 40227 Taiwan

**Keywords:** Systemic lupus erythematosus, Platelet, Extracellular vesicles, tRNA-derived small RNA, Neutrophil extracellular traps, Interferon-α

## Abstract

**Background:**

Hyperactive neutrophil extracellular traps (NETs) formation plays a crucial role in active severe systemic lupus erythematosus (SLE). However, what triggers the imbalance in dysregulated NETs formation in SLE is elusive. Transfer RNA-derived small RNAs (tsRNAs) are novel non-coding RNAs, which participate in various cellular processes. We explore the role of tsRNAs on NETs formation in SLE.

**Methods:**

We analyzed the levels of NETs DNA and platelet-derived extracellular vesicles (pEVs) from 50 SLE patients and 20 healthy control subjects. The effects of pEVs on NETs formation were evaluated by using immunofluorescence assay and myeloperoxidase-DNA PicoGreen assay. The regulatory mechanism of pEVs on NETs formation and inflammatory cytokines production were investigated using an in vitro cell-based assay.

**Results:**

Increased circulating NETs DNA and pEVs were shown in SLE patients and were associated with disease activity (*P* < 0.005). We demonstrated that SLE patient-derived immune complexes (ICs) induced platelet activation, followed by pEVs release. ICs-triggered NETs formation was significantly enhanced in the presence of pEVs through Toll-like receptor (TLR) 8 activation. Increased levels of tRF-His-GTG-1 in pEVs and neutrophils of SLE patients were associated with disease activity. tRF-His-GTG-1 interacted with TLR8 to prime p47phox phosphorylation in neutrophils, resulting in reactive oxygen species production and NETs formation. Additionally, tRF-His-GTG-1 modulated NF-κB and IRF7 activation in neutrophils upon TLR8 engagement, resulting IL-1β, IL-8, and interferon-α upregulation, respectively.

**Conclusions:**

The level of tRF-His-GTG-1 was positively correlated with NETs formation in SLE patients; tRF-His-GTG-1 inhibitor could efficiently suppress ICs-triggered NETs formation/hyperactivation, which may become a potential therapeutic target.

**Supplementary Information:**

The online version contains supplementary material available at 10.1186/s12964-024-01730-7.

## Abstract (plain language summary)

Neutrophils and platelets are key members in the immunopathogenesis of SLE. EVs play a key role in intercellular communication. Abnormal NETs formation promotes vascular complications and organ damage in SLE patients. tsRNA is a novel regulatory small non-coding RNA and participates in diverse pathological processes. Herein, we showed that SLE patient-derived ICs activates platelets directly, followed by intracellular tRF-His-GTG-1 upregulation, which is loaded into pEVs. The pEV-carried tRF-His-GTG-1 could interact with TLR8 in neutrophils, followed by activation of the downstream signaling pathway, including p47phox-NOX2-ROS, which causes NETs enhancement, while IRF7 promotes the expression of IFN-α. The tRF-His-GTG-1 inhibitor could suppress efficiently SLE ICs-induced NETs formation and pEVs primed NETs enhancement. This study offers new molecular machinery to explain the association between the platelets-derived tsRNAs, pEVs, and hyperactive NETs formation in lupus. tRF-His-GTG-1 may serve as a potential therapeutic target and help to advance our understanding of tsRNAs in SLE pathogenesis.

**Supplementary Information:**

The online version contains supplementary material available at 10.1186/s12964-024-01730-7.

## Background

Systemic lupus erythematosus (SLE) is a systemic autoimmune disease characterized by autoantibody production against nuclear antigens, with resultant immune complexes (ICs) deposition and inflammation in organs [1]. Platelets are potent immune cells capable of a range of immune responses and play a key role in SLE pathogenesis [2]. Recently, Melki et al. demonstrated that platelets are activated through ICs-FcγRIIA interaction in patients with SLE and that they could contribute to the circulating autoantigenic load through the release of mitochondrial antigens [3]. In addition to mitochondria, higher levels of platelet-derived extracellular vesicles (pEVs) have been found in the blood of SLE patients and their levels correlate with disease activity [4, 5].

EVs can circulate through various bodily fluids and carry cellular components (nucleic acids, proteins, and lipids) to mediate intercellular communication and thus play a key role in immune modulation [6]. Transfer RNA (tRNA)-derived small RNAs (tsRNAs) are novel small noncoding RNAs in EVs, which could mediate immune responses and metabolic regulation [7, 8]. Recent studies identified EVs-carried tsRNAs associated with disease activity of SLE, but the regulatory mechanism remains elusive [9, 10].

In addition to platelets, neutrophils have been hypothesized as another prominent factor of the initiation and perpetuation of SLE. SLE-derived low-density granulocytes (LDGs) induce vascular damage and synthesize increased amounts of type I interferons (IFNs) [11]. Moreover, increased cell death and enhanced neutrophil extracellular traps (NETs) formation were observed in SLE patient-derived neutrophils, which play a crucial role in autoimmunity induction and organ damage in this disease [12]. In the present study, we explored the association between pEVs and hyperactive NETs formation, and dissected their regulatory mechanism in SLE pathogenesis.

## Methods

### Subjects

A total of 50 SLE patients fulfilling the 1997 revised criteria of the American College of Rheumatology (ACR) [13] and 2012 Systemic Lupus International Collaborating Clinics (SLICC) classification criteria [14] were included. Twenty healthy volunteers, who had no rheumatic disease, served as normal controls. Inactive and active SLE were defined as SLE Disease Activity Index (SLEDAI) ≤ 4 and > 4, respectively. Demographic and clinical characteristics of SLE patients and healthy control (HC) subjects are listed in Table 1. Sex was not considered as a biological variable. The Institutional Review Board of Taichung Veterans General Hospital approved this study (CF21176A), and the written consent of all participants was obtained according to the Declaration of Helsinki.

**Table 1:** Demographics and clinical characteristics of systemic lupus erythematosus (SLE) patients and healthy control subjects

Variable	SLE patients (*n* = 50)	Healthy controls (*n* = 20)
Age (years)	39.5 ± 14.8	34.1 ± 8.7
Gender (female, %)	40 (80.0)	15 (75.0)
SLEDAI	6.4 ± 3.3	N.A.
≤ 4	20 (40.0)	N.A.
> 4	30 (60.0)	N.A.
Positive anti-dsDNA	30 (60.0)	N.A.
Anti-dsDNA (IU/mL)	180.5 ± 146.4	N.A.
C3 (mg/dL)	82.9 ± 26.3	N.A.
C4 (mg/dL)	16.5 ± 8.5	N.A.
Prednisolone	49 (98.0)	N.A.
Prednisolone dose (mg/day)	13.9 ± 11.8	N.A.
Hydroxychloroquine	45 (90.0)	N.A.

Values are mean ± SD or the number (%) of patients

N.A., not available

### Neutrophils and platelets isolation

Neutrophils were immediately isolated from venous blood by using Polymorphprep (Axis-Shield, USA), according to the manufacturer’s instructions. After centrifugation at 500 × *g* for 30 min at 25 °C, the polymorphonuclear leukocyte (PMN) layer was transferred to a clean tube. The ammonium-chloride-potassium (ACK) lysing buffer (Thermo Fisher Scientific, USA) was added and mixed gently. After 5 min, the mixtures were centrifuged at 500 × *g* for 5 min at 25 °C. The pellets were suspended in 10 ml Hank’s balanced salt solution (HBSS, Sigma-Aldrich, USA) and dispersed gently. After centrifugation at 500 × *g* for 5 min at 25 °C, the neutrophil pellets were obtained and suspended in 1 ml HBSS.

Platelets were harvested from peripheral blood by centrifugation at 230 × *g* for 15 min at 25 °C, followed by centrifugation at 1000 × *g* for 10 min. Pellets were resuspended with Tyrode’s buffer (Sigma-Aldrich, USA) containing 1/6 volumes of acid citrate dextrose (ACD) anticoagulant (Sigma-Aldrich, USA) and 1µM Prostaglandin I2 (PGI2) (Sigma-Aldrich, USA), then centrifuged at 1000 × *g* for 10 min at 25 °C before being resuspended in Tyrode’s buffer containing 1µM PGI2 and 0.04 U/ml apyrase (Sigma-Aldrich, USA) and placed on the shaker. Before use, samples were subjected to centrifugation at 1000 × *g* for 10 min and were resuspended in Tyrode’s buffer.

### EVs isolation and quantification

Samples were centrifuged at 350 × *g* for 10 min at 4 °C to remove cell debris. For analysis of EVs characteristics and functional assays, 2.5 ml of each sample was diluted with 7.5 ml of phosphate buffered saline (PBS) and concentrated using Amicon ultra-0.5 centrifugal filter devices (Millipore, Amicon Ultra 100 K device) at 3000 × *g* for 30 min at 4 °C. One hundred microliters of retentate were diluted with 1.4 ml of PBS and subjected to centrifugation at 10,000 × *g* for 30 min at 4 °C. The pellets were resuspended in 1.5 ml PBS and ultracentrifuged at 120,000 × *g* for 90 min at 4 °C. The pellets were resuspended in 50 µl PBS and stored at -80 °C [15]. The purified EVs were confirmed using transmission electron microscopy image analysis (Hitachi HT7700, Japan), the tunable resistive pulse sensing assay (IZON Science, New Zealand), immunoblotting, and flow cytometry, respectively. The EVs were quantified using a direct ELISA-based method to quantify the EVs surface marker CD63 according to the manufacturer’s instructions (System Biosciences, USA).

### Immune complex isolation

Sera were precipitated with 3% (wt/vol) ice cold polyethylene glycol (PEG) 6000 (Sigma-Aldrich, USA), centrifuged at 2000 × *g* for 20 min at 4 °C, and washed three times in sterile PBS and subsequently diluted to the initial serum volume in sterile PBS [16]. The ICs from SLE patients were verified by anti-DNA ELISA assays (CUSABIO, USA).

### Quantitative PCR for EVs-carried tsRNAs

The total EVs-carried tsRNAs were extracted from the EVs using the rtStar tRF&tiRNA Pretreatment Kit (Arraystar, USA) to remove tRNA modifications. Twenty-five femtomoles of synthetic *Caenorhabditis elegans* miRNA (cel-miR-39, Thermo Fisher Scientific, USA) were added to each sample as the internal control. For cDNA synthesis, 250ng of total tsRNA was reverse transcribed using the rtStar cDNA Synthesis Kit (Arraystar, USA). Real-time PCR quantification of specific tsRNA was performed using the LightCycler 480 SYBR Green I Master (Roche, Germany) with a specific primer set (Supplementary Table 1) and analyzed with the LightCycler 96 real-time PCR System (Roche, Germany) using a standard thermoprofile according to the manufacturer’s protocol. The fold expression of the target gene relative to the averaged internal control gene in each sample was calculated using the comparative threshold cycle (Ct) method and evaluated by 2^dCt^, where dCt = mean of healthy controls (Ct _tsRNAs gene_–Ct _cel−miR−39_) – patient (Ct _tsRNAs gene_–Ct _cel−miR−39_).

### Loading of tsRNAs mimic, or control into EVs

Electroporation was used in loading tRF-His-GTG-1 mimic, or control into human pEVs. In brief, 0.1µmole of tRF-His-GTG-1 mimic, or control were added to 20 µl of human pEVs sample (approximately 5 × 10^8^ particles). The mixtures were electroporated at 500 pulse voltage/10 pulse width (ms)/ 3 pulse number using a Neon Transfection system (Thermo Fisher Scientific). After electroporation, the mixture was immediately treated with one unit of RNase A (QIAGEN, Germany) for 30 min, followed by the addition of the 2 µl RNase inhibitor. tRF-His-GTG-1 mimic-, or control-loaded EVs were extracted by using ultracentrifugation [17].

### Immunofluorescence assay

Human neutrophils from each treatment group were fixed with 4% paraformaldehyde at room temperature for 10 min and then washed three times with PBS. Cells were permeabilized in PBS containing 1% BSA and 0.2% saponin and then blocked for 1 h in PBS containing 2% BSA. For NETs formation analysis, cells were incubated with primary antibodies (mouse myeloperoxidase [MPO] antibody [Santa Cruz, USA] at 1:200) followed by a secondary antibody for MPO detection. DNA was stained with Hoechst 33342 (Thermo Fisher Scientific, USA) and extracellular NETs DNA was detected with the cell-impermeable extracellular DNA dye Sytox Green (5µM, Thermo Fisher Scientific) for 10 min after fixation and permeabilization. The staining procedures were conducted at room temperature and with the samples protected from direct light. Coverslips were mounted onto glass slides with Slow-Fade mounting medium (Thermo Fisher Scientific, USA), and images were observed and recorded on an Olympus FV1000 laser scanning confocal microscope. Images were analyzed using FV10-ASW version 4.2 software.

### Quantification of NETs DNA

Briefly, 100 µl of plasma or neutrophil culture supernatant was incubated overnight in a 96-well plate precoated with anti-MPO antibody (Santa Cruz, USA). The DNA bound to MPO was quantified using the Quant-iT PicoGreen kit (Thermo Fisher Scientific, USA) according to the manufacturer’s instructions. The Mann-Whitney U test was performed for between-group comparisons using GraphPad Prism software version 8.

### RNA-protein pull-down assay

A single biotinylated nucleotide was attached to the 3´ terminuses of the tRF-His-GTG-1 mimic or the control by using the Pierce RNA 3´ End Desthiobiotinylation Kit (Thermo Fisher Scientific, USA) according to the manufacturer’s instructions. Human neutrophils (2 × 10^6^ cells) were transiently transfected with 30nM labeled tRF-His-GTG-1 mimic, or control by using Lipofectamine RNAiMAX Transfection Reagent (Thermo Fisher Scientific, USA) according to the manufacturer’s instructions. Approximately 24 h after transfection, cells were washed with PBS and lysed with lysis buffer. After centrifugation at 12,000 × *g* at 4 °C for 15 min, the protein was collected and quantified. The tRF-His-GTG-1 RNA binding proteins were collected by using the Pierce Magnetic RNA-Protein Pull-Down Kit (Thermo Fisher Scientific) according to the manufacturer’s instructions. The immunoprecipitated proteins were analyzed by immunoblotting with the indicated antibodies.

### Statistics

The results are presented as the mean ± standard deviation (SD). The unpaired, two-tailed Student’s t-test and Mann-Whitney U test was used for between-group comparisons. A one-way analysis of variance (ANOVA) with the Bonferroni post hoc test was used for multiple comparisons. The correlation coefficient was calculated using Spearman’s correlation test. *P* values less than 0.05 were considered statistically significant and tests were performed using GraphPad Prism 8.

## Results

### Increased extracellular vesicles release in the plasma of SLE patients

**Fig. 1:**
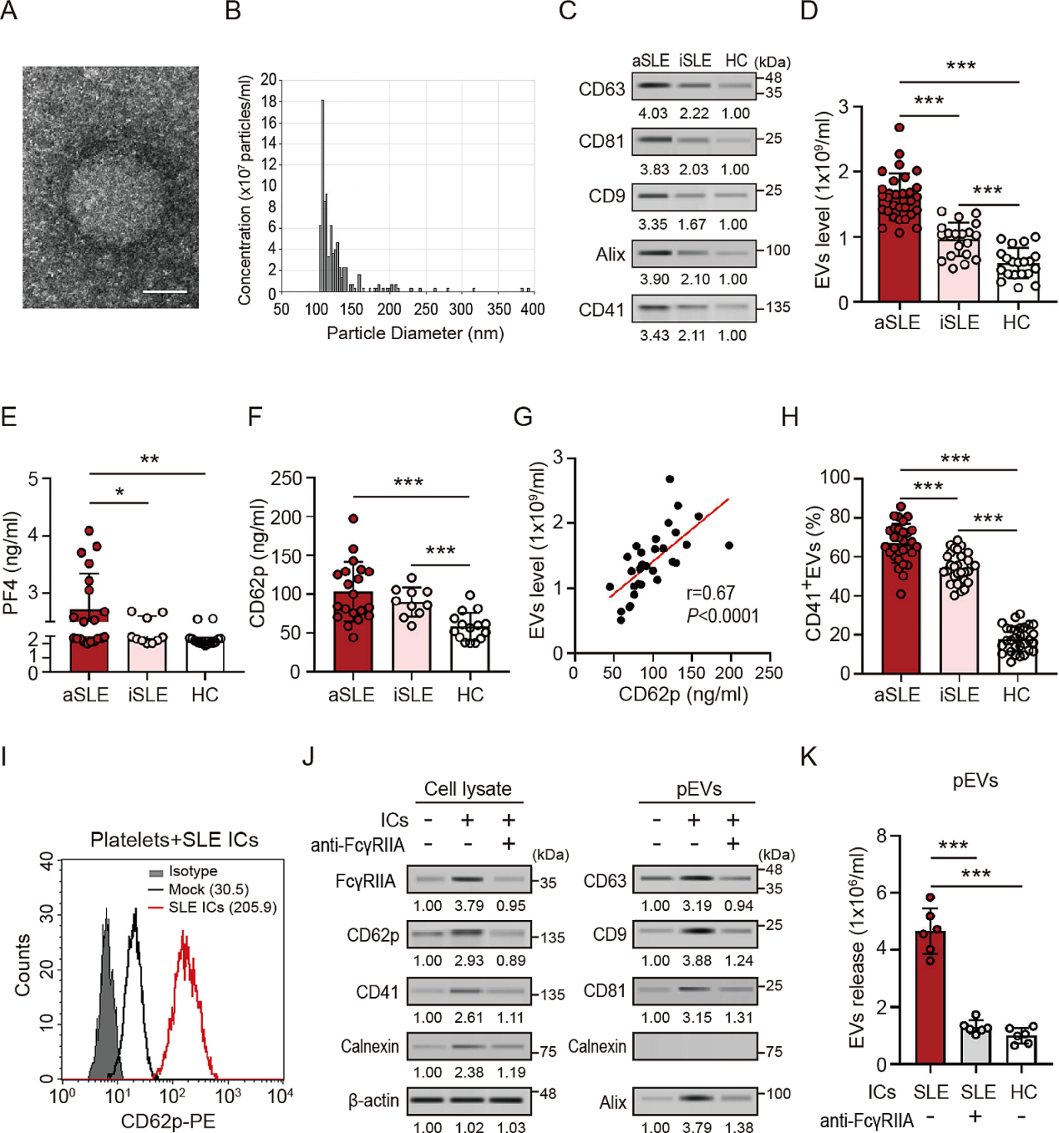
SLE patient-derived immune complexes induced platelets activation and extracellular vesicles production through FcγIIA. (**A**)Transmission electron micrographs of purified extracellular vesicles (EVs) isolated from plasma of SLE patient. The scale bar in the image represents 50 nm. (**B**) The size distribution of EVs isolated from the plasma of SLE patient was analyzed using a tunable resistive pulse sensing assay. (**C**) Expression of EV-specific surface markers (CD63, CD81, CD9, and Alix [ALG-2-interacting protein X]) and a platelet-specific marker (CD41) in EVs from plasma was analyzed using immunoblotting. (**D**) Comparison of EVs levels in the plasma of SLE patients with different activity and HC subjects by using enzyme-linked immunosorbent assay (ELISA)-based assay. (E and F) The levels of (**E**) platelet factor 4 (PF4), and (**F**) CD62p in the plasma of SLE patients. (**G**) A positive correlation between the levels of CD62p and EVs release was shown in SLE patients. (**H**) Increased percentages of pEVs (CD41^+^CD63^+^) were released in the plasma of patients with SLE and are associated with disease severity. (**I** to **K**) SLE patient-derived immune complexes (ICs) induced normal platelets activation, which is accompanied by increased pEVs release through FcγIIA. All experiments were performed in triplicate, and the data are presented as the mean ± SD. The densitometric analysis of immunoblotting were presented in Additional file 2. *P < 0.05, **P < 0.01, ***P < 0.005. aSLE, active SLE; iSLE, inactive SLE; HC, healthy control

The images of transmission electron microscope (Fig. 1A) and the results of tunable resistive pulse sensing assay (Fig. 1B) showed that the average particle size of EVs in the plasma of SLE patients was 120.0 nm, which was consistent with the size of typical small EVs (< 200 nm) [18]. The immunoblotting results showed that SLE patient-derived EVs expressed EVs-specific markers (CD63, CD81, CD9, and Alix) (Fig. 1C). We further compared the levels of EVs in the plasma of SLE patients with different disease severity by using an ELISA-based assay (Fig. 1D). Significantly increased levels of circulating EVs were observed in the plasma of SLE patients compared with those in HC subjects and were associated with disease severity (active SLE: *n* = 30, 1.62 ± 0.36 × 10^9^ EV particles/ml; inactive SLE: *n* = 20, 0.96 ± 0.26 × 10^9^ EV particles/ml versus HC: *n* = 20, 0.59 ± 0.23 × 10^9^ EV particles/ml, *P* < 0.005).

### Increased extracellular vesicles in SLE are associated with platelet activation

Increased levels of platelet activation markers (e.g., platelet factor 4 [PF4], and CD62p) were detected in the plasma of SLE patients and were associated with disease severity (Fig. 1E and F). Moreover, a positive correlation between the levels of CD62p and EVs release was shown in SLE patients (*r* = 0.67, *P* < 0.0001, Fig. 1G). Elevated percentages of pEVs (CD63^+^CD41^+^) were shown in patients with active SLE (66.94 ± 10.01%, Fig. 1H), compared to those in inactive SLE patients (54.38 ± 7.62%, *P* < 0.005) or HC subjects (17.89 ± 6.45%, *P* < 0.005).

We observed that increased CD62p was induced in human platelets after treatment with SLE patient-derived ICs (Fig. 1I), suggesting that SLE ICs could activate platelets. To examine whether SLE ICs-induced platelet activation may contribute to pEVs release through binding to FcγRIIA, normal human platelets were treated with SLE ICs for 4 h in the presence or absence of anti-FcγRIIA antibody. The pEVs were isolated from supernatant using human CD63 Dynabeads magnetic separation technology (Thermo Fisher Scientific, USA). The levels of FcγRIIA, CD62p, and CD41 were significantly induced in platelets after treating with SLE ICs (left panel, Fig. 1J), which were accompanied by increased pEVs release (right panel, Fig. 1J). These effects were suppressed in the presence of anti-FcγRIIA antibody. We further confirmed our observations by using ICs isolated from different SLE patients (*n* = 6) and HC subjected (*n* = 6), and measured the levels of pEVs (CD63^+^CD41^+^) release using ELISA-based analysis (Fig. 1K, *P* < 0.005).

### SLE patient-derived pEVs induced NETs formation

**Fig. 2:**
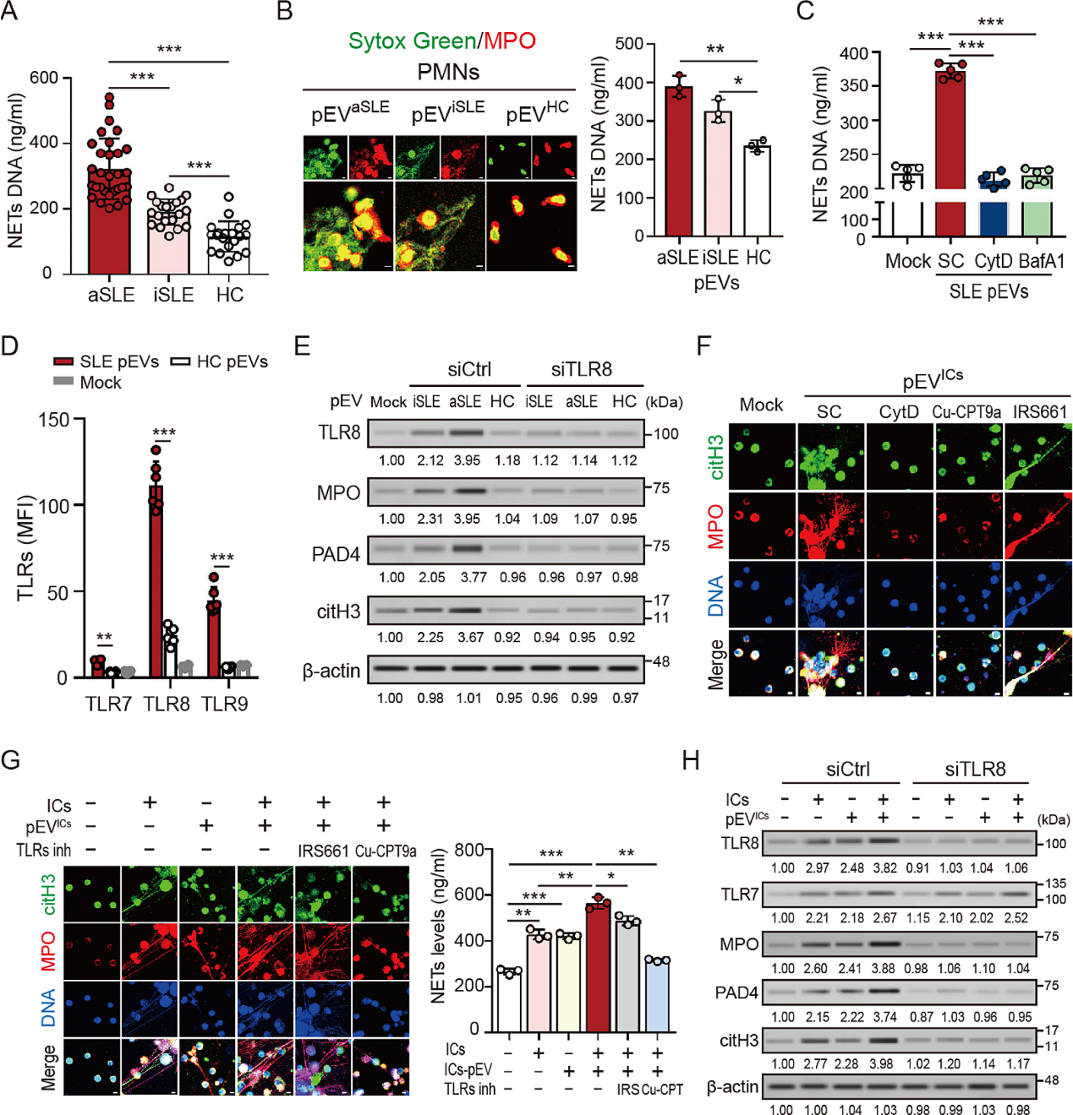
SLE patient-derived pEVs induced NETs formation. (**A**) Increased NETs DNA release in plasma of SLE patients with different disease activity. (**B**) Increased levels of NETs DNA were detected in normal human neutrophils after treatment with SLE patient-derived pEVs (approximately 5 × 10^8^ particles), and (**C**) this effect was decreased in the presence of the endocytosis inhibitor cytochalasin D (Cyt D, 10µM) or the vacuolar type H^+^-ATPase inhibitor bafilomycin A1 (BafA1, 100nM). (**D**) A dramatically elevated level of TLR8 was shown in normal neutrophils after treatment with SLE pEVs, compared to those with healthy control (HC) subject-derived pEVs treatment or mock control cells. (**E** and **F**) SLE pEVs induced NETs formation through TLR8 activation (**E**, left panel). This effect was diminished in TLR8 knockdown cells (**E**, right panel), and (**F**) in the presence of Cyt D or the TLR8-specific inhibitor Cu-CPT9a (10µM). (**G**) pEV^ICs^ enhanced ICs-induced NETs formation, and this effect was suppressed in the presence of Cu-CPT9a, or in (**H**) TLR8 knock-down cells. The scale bar in the IFA image represents 5 μm. All experiments were performed in triplicate, and the data are presented as mean ± SD. The densitometric analysis of immunoblotting were presented in Additional file 2. *P < 0.05, **P < 0.01, ***P < 0.005

Next, we compared the levels of NETs DNA release in SLE patients with different disease activity. Elevated levels of NETs DNA were detected in SLE patients with different severity (active SLE: *n* = 30, 322.5 ± 92.5 ng/ml, *P* < 0.005; inactive SLE: *n* = 20, 190.0 ± 39.8 ng/ml, *P* < 0.005; Fig. 2A), compared to those in HC subjects (*n* = 20, 114.7 ± 46.4 ng/ml). To examine the effect of SLE patient-derived pEVs on NETs formation, human neutrophils were stimulated with active SLE-, inactive SLE-, and HC subject-derived pEVs (approximately 5 × 10^8^ particles) for 4 h, respectively. Significantly increased NETs formation was shown in neutrophils after stimulation with SLE pEVs (active SLE: 390.32 ± 27.75 ng/ml; inactive SLE: 326.30 ± 28.71 ng/ml, Fig. 2B), compared with those in HC subject-derived pEVs (235.31 ± 15.01 ng/ml, *P* < 0.05). This effect decreased in the presence of the endocytosis inhibitor cytochalasin D (372.30 ± 11.02 versus 213.80 ± 9.97 ng/ml, *P* < 0.005, Fig. 2C) and the vacuolar type H^+^-ATPase inhibitor bafilomycin A1 (219.42 ± 10.68 ng/ml, *P* < 0.005), suggesting that SLE pEVs induced NETs formation by uptake and activation of endolysosomal Toll-like receptors (e.g., TLR3, 7, 8 and 9).

Given that mature neutrophils express all TLRs except TLR3 [19], we analyzed the intracellular expressions of TLR7, TLR8, and TLR9 in human neutrophils after treatment with pEVs from SLE patients (*n* = 6) and HC subjects (*n* = 6). Compared to TLR7 and TLR9, a dramatically elevated level of TLR8 was shown in neutrophils after treatment with SLE pEVs (MFI, 111.31 ± 13.87 versus 24.47 ± 5.02, *P* < 0.005, Fig. 2D). We further confirmed the effect of TLR8 on SLE pEVs-induced NETs formation by using TLR8 knockdown cells. As shown in Fig. 2E, SLE pEVs-induced NETs-associated proteins (e.g., MPO, peptidylarginine deiminases 4 [PAD4], and citrullinated histone H3 [citH3]) were almost completely suppressed in TLR8-knockdown cells.

Next, we explored whether SLE ICs-induced pEVs (pEV^ICs^) may contribute to NETs formation. Significantly increased NETs formation was induced in normal neutrophils after treatment with pEV^ICs^ (Fig. 2F), and this effect was suppressed in the presence of cytochalasin D or the TLR8-specific inhibitor Cu-CPT9a, but no effect was shown in cells after treatment with the TLR7-specific inhibitor IRS661. Our results suggested that pEV^ICs^ may carry specific ssRNA to induce NETs formation directly by uptake and TLR8 activation. Additionally, it is worth noting that dramatically increased ICs-primed NETs formation was shown in the presence of pEV^ICs^ (565.2 ± 24.74 ng/ml versus 427.7 ± 21.59 ng/ml, *P* < 0.01, Fig. 2G), which suggested that pEV^ICs^ play a crucial role in hyperactive NETs formation. This effect was reduced slightly under IRS661 treatment (487.8 ± 20.63, *P* < 0.05), but was almost completely suppressed in the presence of Cu-CPT9a (315.4 ± 5.01, *P* < 0.01, Fig. 2G). We confirmed this observation by using TLR8 knockdown neutrophils (Fig. 2H and Supplementary Fig. S1), suggesting that ssRNA carried by pEV^ICs^ contribute NETs enhancement through TLR8 activation.

### SLE patient-derived pEVs carried tsRNA to induce NETs formation

**Fig. 3:**
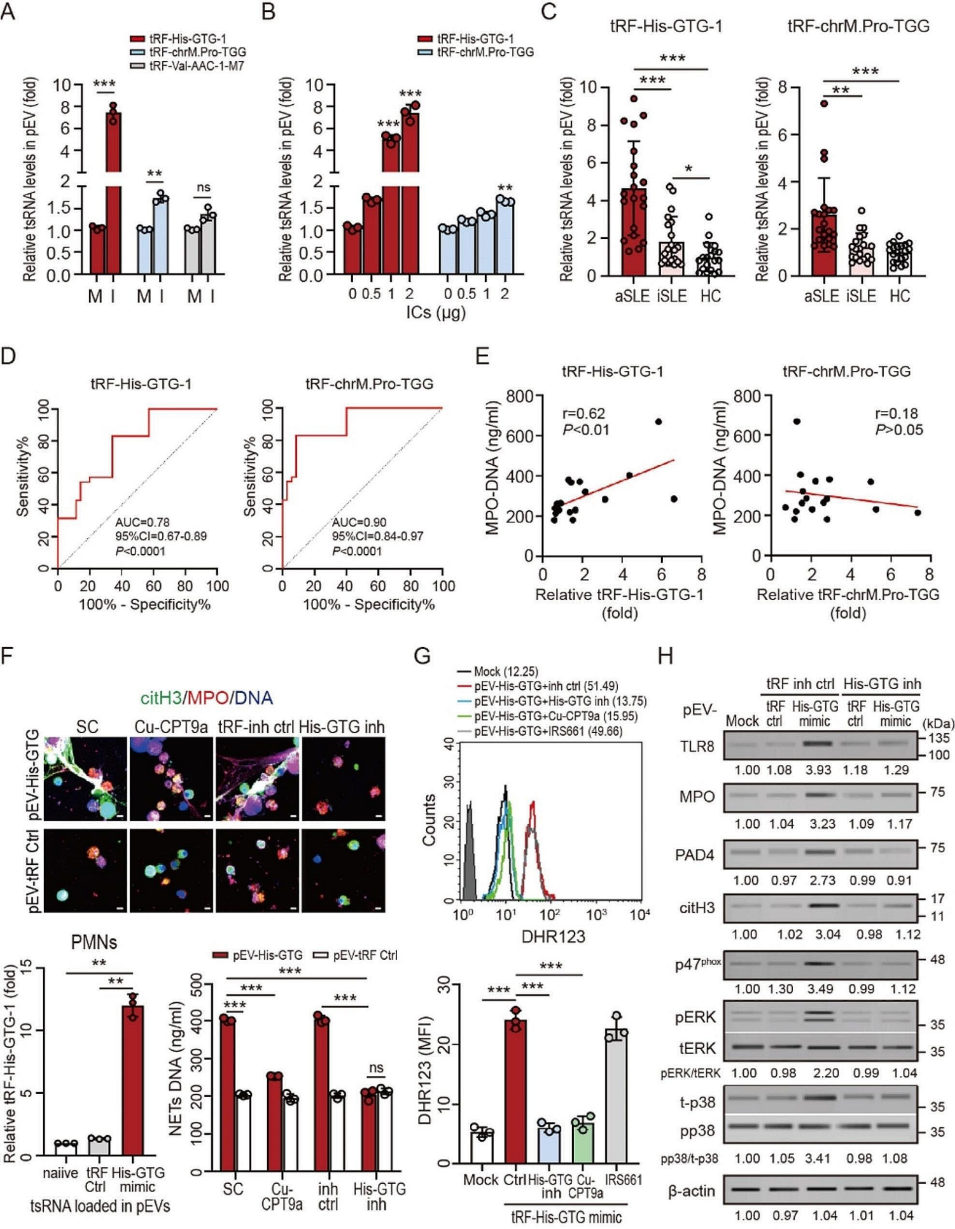
Elevated levels of tRF-His-GTG-1 in SLE patients-derived pEVs induce NETs formation through TLR8 activation and ROS production. (**A**) Increased levels of tRF-His-GTG-1, and tRF-chrM.Pro-TGG were shown in EVs released from normal platelets after stimulation with SLE patients-derived ICs (I) compared to those in mock control cells (M), and (**B**) this effect was dose-dependent. (**C**) Elevated tRF-His-GTG-1 and tRF-chrM.Pro-TGG expression were revealed in pEVs of patients with SLE, and associated with disease severity. (**D**) Receiver operating characteristic (ROC) curve analysis of tRF-His-GTG-1 and tRF-chrM.Pro-TGG expression in SLE patients with different severity (SLEDAI score), respectively. (**E**) The expression of tRF-His-GTG-1 was positively correlated with NETs DNA release levels in patients with SLE. (**F** to **H**) The tRF-His-GTG-1 mimic or mimic control (10µM) were loaded into human platelet-derived EVs (pEVs) using electroporation. Human neutrophils were treated with pEVs that carried-tRF-His-GTG-1 mimic or mimic control in the presence of tRF-His-GTG-1 inhibitor, tsRNA inhibitor control, or TLR8 inhibitor for 4 h. (**F**) NETs formation was observed using confocal microscopy (upper panel) and quantified by the MPO-DNA PicoGreen assay (right below panel). The intracellular tRF-His-GTG-1 in the neutrophils were measured by QRT-PCR (Left below panel). (**G**) The levels of cytosolic ROS were detected using flow cytometry with dihydrorhodamine (DHR) 123 staining. (**H**) The expression of intracellular TLR8 and NETs-associated proteins was analyzed by using immunoblotting. Immunoblotting bands from β-actin were densitometrically measured by ImageJ to determine the lane normalization factor for samples. The scale bar in the IFA image represents 5 μm. The image shown is from a single experiment that is representative of at least three separate experiments. Data are presented as the mean ± SD. The densitometric analysis of immunoblotting were presented in Additional file 2. *P < 0.05, **P < 0.01, ***P < 0.005. ns, non-significant

Yang et al. identified that several EV-carried tsRNAs were upregulated in SLE patients, but their pathogenic role was unclear [9]. Based on their results, we choose three candidates (tRF-His-GTG-1, tRF-chrM.Pro-TGG and tRF-Val-AAC-1-M7) for further study. We assessed the association between these tsRNA candidates, pEVs, and SLE disease. Human platelets were stimulated with different SLE ICs (*n* = 3) for 1 h, and the levels of tsRNAs loaded into pEVs were quantified using QRT-PCR (Fig. 3A). There was no significant difference in the expression of tRF-Val-AAC-1-M7. But significantly increased levels of tRF-His-GTG-1 (7.42 ± 0.75-fold, *P* < 0.005) and tRF-chrM.Pro-TGG (1.67 ± 0.04-fold, *P* < 0.01) were both shown in pEVs released from platelets after stimulation with SLE ICs, and this effect was dose-dependent (Fig. 3B). We validated the levels of tRF-His-GTG-1 and tRF-chrM.Pro-TGG in pEVs by using QRT-PCR (Fig. 3C). Elevated tRF-His-GTG-1 (active SLE: 4.65 ± 2.49-fold, *P* < 0.005; inactive SLE: 1.84 ± 1.32-fold, *P* < 0.05; HC: 1.00 ± 0.76-fold), and tRF-chrM.Pro-TGG (active SLE: 2.60 ± 1.57-fold, *P* < 0.005; inactive SLE: 1.22 ± 0.60-fold; HC: 1.00 ± 0.39-fold) expression was revealed in pEVs of SLE patients and associated with disease severity (SLEDAI score) (tRF-His-GTG-1: AUC = 0.86, 95% CI = 0.77–0.94, *P* < 0.0001; tRF-chrM.Pro-TGG: AUC = 0.92, 95% CI = 0.85–0.99, *P* < 0.0001, Fig. 3D). We further analyzed the association between pEVs-carried tsRNAs and NETs DNA release. The expression of tRF-His-GTG-1 was positively correlated with NETs DNA release (*r* = 0.63, *P <* 0.01, Fig. 3E), but tRF-chrM.Pro-TGG was not.

### Platelet-derived tRF-His-GTG-1-induced NETs formation is TLR8 dependent

**Fig. 4:**
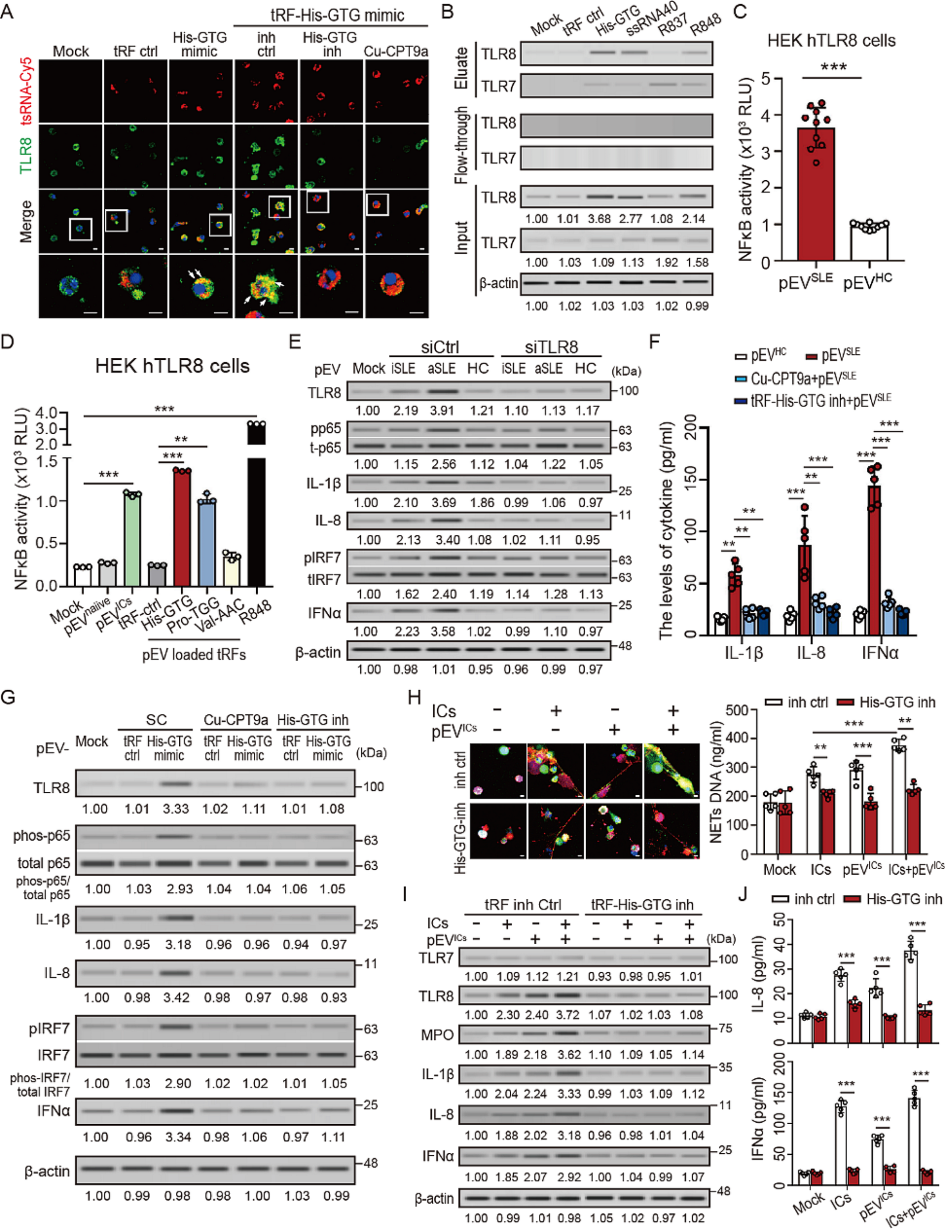
pEVs-carried tRF-His-GTG-1 enhanced NETs formation and induced IL-1β/IL-8/interferon-α upregulation by TLR8 activation. (**A**) Human platelet-derived EVs (pEVs) were loaded with Cy5-labeled tRF-His-GTG-1 mimic (red) or mimic control (red) and then co-cultured with human neutrophils for 4 h. Endogenous TLR8 was stained with TLR8 antibodies (green). The tRF-His-GTG-1 transmission and endogenous TLR8 in neutrophils was detected by using the immunofluorescence assay. The scale bar in the image represents 10 μm. (**B**) Human neutrophils were treated with tRF-His-GTG-1 mimic, mimic control, TLR8- (ssRNA40), TLR7- (R837), or TLR7/8- (R848) agonist, respectively. The RNA-binding protein was collected using the RNA-protein pull-down assay kit and analyzed by immunoblotting with specific TLR7-, and TLR8 antibodies. (**C** and **D**) The HEK-Blue hTLR8 cells were stimulated with pEVs from the indicated individuals or (**D**) indicated tsRNA-loaded pEVs for 24 h. NF-κB activation was evaluated in terms of luciferase activity compared to control cells. (**E** to **G**) Human neutrophils were treated with pEVs from the indicated individuals or indicated tsRNA-loaded pEVs in TLR8 knock-down cells, or in the presence of TLR8 agonist for 24 h. The (**E** and **G**) intracellular and (**F**) extracellular proinflammatory cytokines, and interferon α were analyzed using immunoblotting and ELISA, respectively. (**H** to **J**) tRF-His-GTG-1 inhibitor suppressed SLE-ICs/SLE-ICs-primed pEVs induced (**H**, **I**) NETs formation/ hyperactivation, and (**J**) IL-1β, IL-8 and interferon α production. Immunoblotting bands from β-actin were densitometrically measured by ImageJ to determine the lane normalization factor for samples. The scale bar in the IFA image represents 5 μm. The image shown is from a single experiment that is representative of at least three separate experiments. Data are presented as the mean ± SD. The densitometric analysis of immunoblotting were presented in Additional file 2. **P < 0.01, ***P < 0.005

Therefore, we focused on the effect of pEVs-carried tRF-His-GTG-1 on NETs formation. We loaded tRF-His-GTG-1 mimics or tsRNA control mimics into human pEVs by using electroporation [20]. The tRF-His-GTG-1-loaded pEVs were then added to human neutrophils. After 4 h, significantly increased intracellular tRF-His-GTG-1 levels (left lower panel, Figs. 3F and 4A), accompanied by elevated NETs formation (upper panel, Fig. 3F) and NETs DNA release (right lower panel, Fig. 3F), were observed in neutrophils with the addition of tRF-His-GTG-1 mimics-loaded pEVs. This effect was almost completely suppressed in the presence of the TLR8-specific inhibitor or tRF-His-GTG-1 inhibitor. Our results showed that ICs primed platelet-derived tRF-His-GTG-1 could induce NETs formation directly through EVs transmission and TLR8 activation.

Reactive oxygen species (ROS) are essential in the regulation of NETs formation in SLE [12]. We showed that pEV-carried tRF-His-GTG-1 induced cytosolic ROS production in neutrophils through TLR8 activation (Fig. 3G). The phosphorylation of p47phox is a key factor in ROS production, which is regulated by the TLR8-mediated ERK (extracellular-signal-regulated kinase) and p38 MAPK (mitogen-activated protein kinase) signaling pathways [21]. We showed that elevated levels of ERK and p38 MAPK activation were induced in cells with tRF-His-GTG-1-loaded pEVs treatment, accompanied by increased p47phox phosphorylation and the enhancement of NETs-associated proteins. These effects were inhibited efficiently in the presence of tRF-His-GTG-1 inhibitor (Fig. 3H), or Cu-CPT9a (Supplementary Fig. S2).

### tRF-His-GTG-1 interacts with TLR8

Next, we assessed the association between SLE ICs and tRF-His-GTG-1 expression in neutrophils. Increased levels of intracellular tRF-His-GTG-1 were revealed in normal neutrophils after treatment of ICs from different SLE patients (*n* = 5), and the effect was dose-dependent (*P* < 0.005, Supplementary Fig. S3A). A dramatically elevated intracellular tRF-His-GTG-1 was detected in ICs-primed neutrophils in the presence of pEV^ICs^ (*P* < 0.01, Supplementary Fig. S3B), and this effect was diminished in the presence of cytochalasin D (*P* < 0.005, Supplementary Fig. S3B). We further examined whether tRF-His-GTG-1 could bind to TLR8 on neutrophils by using immunofluorescence assay. Platelet-derived EVs were loaded with Cy5-labeled tRF-His-GTG-1 mimic or mimic control and then co-cultured with human neutrophils for 4 h. The tRF-His-GTG-1 transmission and endogenous TLR8 in human neutrophils were detected by using immunofluorescence assay. As shown in Fig. 4A, colocalization of tRF-His-GTG-1 and TLR8 was detected in neutrophils, and this effect was inhibited in the presence of tRF-His-GTG-1 inhibitor or Cu-CPT9a. We further performed RNA-protein pull-down assay to confirm that tRF-His-GTG-1 could induce endogenous TLR8 expression and interact with TLR8 directly (Fig. 4B).

### pEVs carried tRF-His-GTG-1 induced IL-1β/IL-8/interferon-α upregulation in neutrophils through TLR8 activation

IL-1β, IL-8 and IFN-α were significantly increased in neutrophils of SLE patients and correlated with disease activity [22, 23], which were regulated by NF-κB and IRF7 signaling pathways through TLR8 and adaptor protein MyD88 activation, respectively [24]. Therefore, we examined whether SLE pEVs could regulate NF-κB activity by TLR8 activation. As shown in Fig. 4C, elevated NF-κB activity was revealed in cells with SLE pEVs modulation upon TLR8 engagement by using the HEK-hTLR8 cell model (InvivoGen, USA). In addition to NF-κB, increased levels of IRF7 activation were detected in neutrophils with SLE pEVs (Fig. 4E), accompanied by upregulated IL-1β, IL-8, and IFNα (Fig. 4E and F). These effects were diminished in TLR8-knockdown cells (Fig. 4E) or in the presence of Cu-CPT9a and tRF-His-GTG-1inhibitor (Fig. 4F). Consistent with this, tRF-His-GTG-1-loaded pEVs also had the same effects on NF-κB activation (Fig. 4D), the phosphorylation of p65 subunits and IRF7 activation (Fig. 4G), resulting in elevated IL-1β, IL-8, and IFNα. The above effects were completely inhibited in the presence of the Cu-CPT9a, or tRF-His-GTG-1 inhibitor.

### tRF-His-GTG-1 inhibitor suppressed SLE-derived ICs primed NETs formation and IL-1β/IL-8/interferon-α production

Finally, we assessed the effects of tRF-His-GTG-1 inhibitor on SLE ICs (*n* = 5) and/or ICs-primed pEVs on NETs formation/hyperactivation. As shown in Fig. 4H, SLE ICs and/or ICs-primed pEVs induced NETs formation/ hyperactivation was significantly suppressed in the presence of tRF-His-GTG-1 inhibitor (*P* < 0.005), accompanied by downregulated IL-1β, IL-8, and IFNα (Fig. 4I and J).

## Discussion

Neutrophils have a variety of crucial biological functions in both innate and adaptive immunity. The release of NETs has been attributed to the capture and elimination of pathogens [25]. Increasing evidence has proven that NETs have emerging roles in noninfectious diseases, including SLE. Garcia-Romo et al. [26] demonstrated that ribonucleoprotein-containing ICs isolated from patients with SLE can induce neutrophil activation and NET formation. Recent studies have shown that NETs contain bioactive cytokines (e.g., IL-17 A, IL-33), resulting in thromboinflammation and IFN-α production in patients with SLE [27, 28]. Impaired degradation of NETs has been identified in some patients with SLE and is thought to be due to increased deoxyribonuclease 1 inhibitors and production of anti-NETs antibodies [29]. Additionally, excessive NET formation has been reported in the neutrophils of patients with severe SLE, resulting in coagulopathy and immunothrombosis [12, 27, 28]. However, the factors that trigger the imbalance in dysregulated NET formation in patients with severe SLE remain unclear. In the present study, we observed that platelet-derived EVs of patients with SLE not only directly induce NET formation but also enhance IC-primed NET formation.

Platelets are derived from mature megakaryocytes in bone marrow and circulate in the blood [30]. In addition to their role in preservation of vascular integrity, platelets also regulate immunological responses. IFN‑α plays a key role in the development of SLE [31]. Duffau et al. [32] reported that activated platelets caused aberrant activation of IFN-α in patients with SLE via release of CD40 ligand (CD40L, also called CD154). Melki et al. [3] demonstrated that in lupus-prone transgenic mice, ICs triggered platelet activation and released mitochondrial antigens. A recent study showed that platelet activation trigger EVs release [33]. In the present study, we observed that ICs of patients with SLE activated platelets through FcγRIIA, and this was accompanied by pEV release. Moreover, IC-triggered pEVs enhanced IC-primed NET formation and IFN-α production in neutrophils through uptake and TLR8 activation, suggesting that platelet-derived ssRNA carried by pEVs is involved in the pathogenesis of SLE.

Recent evidence has revealed that non-coding RNAs, including long non-coding RNAs, microRNAs, and tsRNAs, play key roles in the pathogenesis of SLE [9, 10, 34]. tsRNA expression is spatially and temporally controlled under physiological conditions, thus playing an important role in many biological processes [35]. However, the biological functions of tsRNAs in patients with SLE are illusive. In the present study, the levels of tRF-His-GTG-1 were elevated in the pEVs from patients with SLE, which is consistent with the findings of other studies [9, 10]. Tao et al. [36] showed that tRF-His-GTG-1 regulates the HIF1α/ANG axis in response to hypoxia and mediates LATS2 to promote colorectal cancer progression. Pawar et al. [37] found that Mycobacterium tuberculosis induced abundant accumulation of tRF-His-GTG-1 in human monocyte-derived macrophage-secreted EVs to activate TLR7 in recipient cells. We found that ICs in patients with SLE induced tRF-His-GTG-1 expression in platelets in a dose-dependent manner. Upregulated platelet-derived tRF-His-GTG-1 was carried by pEVs and delivered to neutrophils. We also found that tRF-His-GTG-1 interacted with TLR8 in neutrophils, and this was accompanied by ROS production and NET formation. These effects were completely suppressed in the presence of the TLR8-specific antagonist Cu-CPT9a or tRF-His-GTG-1 inhibitor. tRF-His-GTG-1 inhibitor is an RNA oligonucleotide with specific 2’-O-methyl modification, and recent studies have confirmed that this tRF-His-GTG-1 antagomir performs the same function as the tRF-His-GTG inhibitor [9, 36]. Our result suggest that tRF-His-GTG-1 inhibitor is a potential therapeutic tool to inhibit hyperactive NET formation in patients with severe SLE. Further in-depth in vivo experiments are needed to confirm our observations.

In addition to our findings regarding tRF-His-GTG-1, we also showed that TLR8 plays a crucial role in NET formation and IFN-α production in patients with SLE. Increased levels of TLR8 have been observed in patients with SLE [38]. Lood et al. [39] demonstrated that IC-induced TLR7/8 activation shifted neutrophils away from phagocytosis of ICs toward NETosis. Krug et al. [17] observed that TLR7/8 regulated type I interferon signaling in peripheral blood mononuclear cells. Karama [21] revealed that TLR8, but not TLR7, induced the priming of p47phox phosphorylation in human neutrophils, thus triggering NET formation. We showed that IC-triggered tRF-His-GTG-1–induced NET formation is TLR8-dependent. We also proved that tRF-His-GTG-1 directly interacts with TLR8. Human neutrophils express more TLR8 than TLR7 [19]. Although our results revealed that tRF-His-GTG-1 also interacts with TLR7, the effect of TLR7 on NET formation was lower than that of TLR8. Our results revealed that SLE IC-primed platelet-derived tRF-His-GTG-1 interacts with TLR8 of neutrophils through pEV delivery, resulting in ERK/p38 MAPK activation and p47phox phosphorylation to promote ROS production and the enhancement of NET formation. A TLR8 antagonist could suppress the above effects and may therefore be a potent therapeutic target for SLE.

In addition to NET formation, TLR8 activation leads to the production of a variety of NF-κB-mediated cytokines/chemokines (e.g., IL-1β, IL-8) and type I IFNs in neutrophils [22–24]. We showed that in SLE, ICs upregulate tRF-His-GTG-1 to induce IL-1β, IL-8, and IFN-α production in neutrophils through TLR8 activation. Either a tRF-His-GTG-1 inhibitor or a TLR8 antagonist could efficiently suppress IC-primed inflammatory cytokines/chemokines and IFN-α production. The tRF-His-GTG-1-TLR8 axis might serve as a novel target for drug development against hyperactive inflammation in patients with SLE. Further in vivo experiments are ongoing to validate our in vitro observations.

Despite its novel findings, this pilot study had two main limitations. First, the number of patients with SLE enrolled in our study was quite limited. Further larger and in-depth studies are required to confirm our observations. Second, this study was cross-sectional in design, and we cannot exclude the possibility that NET formation, EV production, or tsRNA expression varied with the therapeutic strategy.

**Fig. 5:**
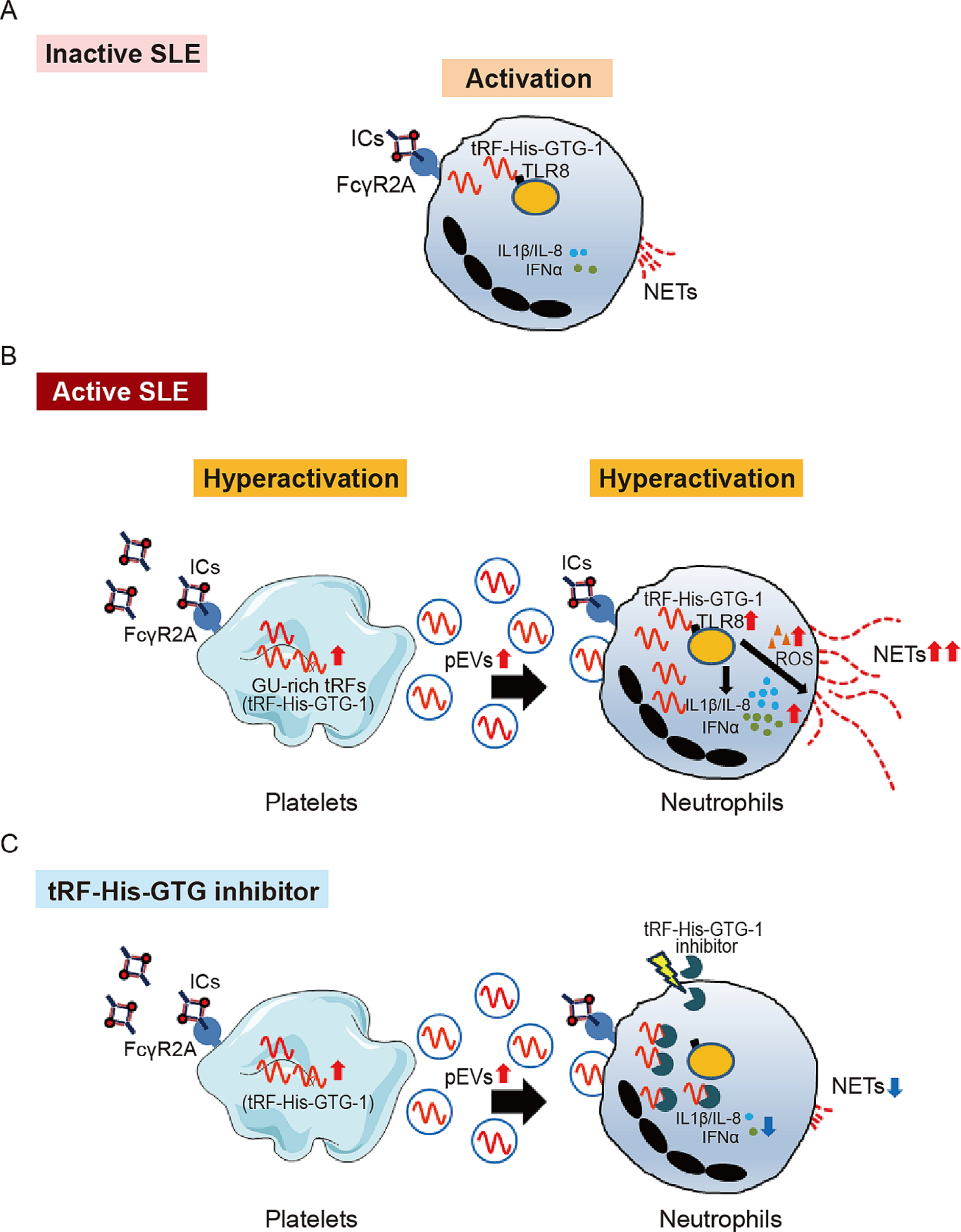
Proposed model for the biological role of tRF-His-GTG-1, TLR8, and platelets in NETs formation/hyperactivation, based on the results of this study. (**A**) SLE patient-derived immune complexes (ICs) induce elevated tRF-His-GTG-1 expression in neutrophils and then stimulate TLR8 activation and NETs formation. (**B**) In addition to neutrophils, ICs induce tRF-His-GTG-1 expression in the platelet in a dose-dependent matter. ICs-primed platelet-derived tRF-His-GTG-1 enhance the original ICs-induced NETs formation and inflammatory cytokines/IFNα production through extracellular vesicles (EVs) transmission. (**C**) tRF-His-GTG-1 inhibitor or TLR8 antagonist suppresses ICs-triggered NETs formation/hyperactivation

In this study, we found that SLE ICs induced tRF-His-GTG-1 upregulation, accompanied by TLR8 activation in neutrophils through FcγRIIA (Fig. 5A). We proved that tRF-His-GTG-1 interacts with TLR8 directly and that this is accompanied by ROS production, NET formation, and IL-1β/IL-8/IFNα production. In addition to neutrophils, SLE-derived ICs activated platelets, and this activation was accompanied by tRF-His-GTG-1 upregulation and pEV release. We also demonstrated that IC-primed platelet-derived tRF-His-GTG-1 enhanced NET formation and IL-1β/IL-8/IFNα production through pEV delivery (Fig. 5B). Moreover, our in vitro cell-based results showed that tRF-His-GTG inhibitor efficiently suppressed SLE IC-primed NET formation and IL-1β/IL-8/IFNα production (Fig. 5C). Therefore, the tRF-His-GTG-TLR8 axis may be a potential therapeutic target for SLE.

## Conclusions

Accumulating evidence shows that tsRNA is a novel category of regulatory noncoding RNA with a distinct biological function and potential application as a therapeutic target [8]. EVs are key mediators of intercellular communication that regulate diverse biological processes, and they may serve as novel targets for therapeutic intervention. This study introduces new molecular machinery to explain the association between platelets and hyperactive NET formation in patients with lupus. tRF-His-GTG-1 may serve as a potential therapeutic target and help to advance our understanding of tsRNAs in the pathogenesis of SLE.

### Electronic supplementary material

Below is the link to the electronic supplementary material.

Supplementary Material 1

Supplementary Material 2

Supplementary Material 3

## Data Availability

No datasets were generated or analysed during the current study.

## Abbreviations

ACD: Acid citrate dextrose
ACK: Ammonium-chloride-potassium
ACR: American College of Rheumatology
ANOVA: A one-way analysis of variance
CCD: Charge-coupled device
CD40L: CD40 ligand
citH3: citrullinated histone H3
Ct: threshold cycle
DHR: dihydrorhodamine
DNase 1: deoxyribonuclease 1
ERK: extracellular-signal-regulated kinase
HBSS: Hank’s balanced salt solution
ECL: enhanced chemiluminescence
HC: healthy control
ICs: immune complexes
IFNs: interferons
LDGs: low-density granulocytes
lncRNAs: long non-coding RNAs
MAPK: mitogen-activated protein kinase
MFI: mean fluorescence intensity
miRNAs: microRNAs
MPO: myeloperoxidase
ncRNAs: non-coding RNAs
NETs: neutrophil extracellular traps
PAD4: peptidylarginine deiminases 4
PBS: phosphate buffered saline
PEG: polyethylene glycol
PGI2: Prostaglandin I2
pEVs: platelet-derived extracellular vesicles
PF4: platelet factor 4
PMN: polymorphonuclear leukocyte
PVDF: polyvinylidene difluoride
ROS: reactive oxygen species
SD: standard deviation
SDS-PAGE: sodium dodecyl sulfate -polyacrylamide gel electrophoresis
SLE: systemic lupus erythematosus
SLICC: Systemic Lupus International Collaborating Clinics
SLEDAI: SLE Disease Activity Index
ssRNA: single-stranded RNA
TLR: Toll-like receptor
tsRNAs: transfer RNA-derived small RNAs
