# Supplementary material for: tRF-His-GTG-1 enhances NETs formation and interferon-α production in lupus by extracellular vesicle
Source: Cell Commun Signal. 2024 Jul 7;22:354. doi: 10.1186/s12964-024-01730-7 (PMC11229248; doi:10.1186/s12964-024-01730-7)
Supplement: Supplementary file 1 — Supplementary Material 1 [file 12964_2024_1730_MOESM1_ESM.pdf]

Supplementary information

tRF-His-GTG-1 enhances NETs formation and interferon-α production in systemic lupus erythematosus by extracellular vesicles delivery

Yi-Ming Chen[1-4,8], Kuo-Tung Tang[4], Hung-Jen Liu[2,3,5,6], Shih-Ting Huang[7], Tsai-Ling Liao[1-3*]

[1]Department of Medical Research, Taichung Veterans General Hospital, Taichung, 40705, Taiwan

[2]Rong Hsing Research Center for Translational Medicine, National Chung Hsing University, Taichung, 40227, Taiwan

[3]Ph.D. Program in Translational Medicine, National Chung Hsing University, Taichung, 40227, Taiwan

[4]Division of Allergy, Immunology and Rheumatology, Department of Internal Medicine, Taichung Veterans General Hospital, Taichung, 40705, Taiwan

[5]Institute of Molecular Biology, National Chung Hsing University, Taichung, 40227, Taiwan

[6]The iEGG and Animal Biotechnology Center, National Chung Hsing University, Taichung, 40227, Taiwan

[7]Division of Nephrology, Department of Internal Medicine, Taichung Veterans General Hospital, Taichung, 40705, Taiwan

[8]Department of Post-Baccalaureate Medicine, College of Medicine, National Chung Hsing University, Taichung, 40227, Taiwan

Corresponding author and address reprint requests:

Dr. Tsai-Ling Liao, Department of Medical Research, Taichung Veterans General Hospital, Taichung, Taiwan

Address: No.1650, Sec.4, Taiwan Boulevard, Xitun Dist., Taichung City 40705, Taiwan

Tel.: 886-4-23592525, extension 4020; Fax: 886-4-23592705

Email: tlliao@vghtc.gov.tw

Supplementary Materials

Reagent or Resource	Source	Identifier (Cat No.)
Antibodies		
Mouse anti-Alix antibody	Santa Cruz	sc-53540
Mouse anti-CD9 antibodies	Abcam	ab58989
Mouse anti-CD63 antibodies	Abcam	ab68418
Mouse anti-CD81 antibodies	Abcam	ab79559
Mouse anti-citH3 antibodies	Abcam	ab5103
Rabbit anti-Erk1/2 antibodies	Cell Signaling Technology	#4695
Mouse anti-IL-1β antibodies	Santa Cruz	sc-52012
Mouse anti-IL-8 antibodies	Santa Cruz	sc-8427
Mouse anti-MPO/Myeloperoxidase antibodies (WB)	Santa Cruz	sc-52707
Mouse anti-MPO/Myeloperoxidase antibodies (ELISA)	Bio-Rad	MCA1757
Mouse anti-NFκB p65 antibodies	Santa Cruz	sc-8008
Mouse anti-p-NFκB p65 antibodies	Santa Cruz	sc-136548
Rabbit anti-p38 MAPK Antibody	Cell Signaling Technology	#9212
Rabbit anti-phospho p38 MAPK Antibody	Cell Signaling Technology	#9211
Mouse anti-p47phox antibodies	Santa Cruz	sc-17845
Mouse anti-TLR7 antibodies	Santa Cruz	sc-57463
Mouse anti-TLR8 antibodies	Santa Cruz	sc-373760
Mouse anti-TSG101 antibodies	Santa Cruz	sc-7964
Rabbit anti-CD41 antibodies	Cell Signaling Technology	#13807

Rabbit anti-IFNα antibodies	Abcam	ab191903
Rabbit anti-IRF-7 antibodies	Cell Signaling Technology	#4920
Rabbit anti-phospho-IRF-7 antibodies	Cell Signaling Technology	#5184
Rabbit anti-PAD4 antibodies	Abcam	ab96758
Rabbit anti-TLR8 antibodies	Abcam	ab24185
Cell lines		
HEK-Blue^{TM} hTLR8 cells	InvivoGen	hkb-htlr8
Chemicals and Assay kits		
Apyrase	Sigma-Aldrich	A6535
Bafilomycin A1	Sigma-Aldrich	B1793
Citrate-dextrose solution	Sigma-Aldrich	C3821
Cytochalasin D	Sigma-Aldrich	C8273
CU-CPT9a	InvivoGen	inh-cc9a
Dihydrorhodamine 123	Thermo Fisher Scientific	D23806
Hank's balanced salt solution	Sigma-Aldrich	55021C
Hoechst 33342	Thermo Fisher Scientific	H3570
Imiquimod	Invivogen	tlrl-imqs
Polymorphprep	Axis-Shield	1895
Prostaglandin I2	Sigma-Aldrich	P6188
Tyrode's buffer	Sigma-Aldrich	T2397
Resiquimod	Invivogen	tlrl-r848
ssRNA40/LyoVec	Invivogen	tlrl-lrna40
SYTOX Green	Thermo Fisher Scientific	S7020
ExoQuick exosome precipitation solution	System Biosciences	EXOQ5A-1S
ExoQuick-TC	System Biosciences	EXOTC10A-1
Ficoll®-Paque Premium	GE Healthcare Biosciences	GE17-5442-02
LightCycler 480 SYBR Green I Master	Roche	04707516001

Pierce RNA 3′ End Desthiobiotinylation Kit	Thermo Fisher Scientific	20163
Pierce Magnetic RNA-Protein Pull-Down Kit	Thermo Fisher Scientific	20164
QIAamp DNA Blood Mini Kit	QIAGEN	51106
rtStar tRF&tiRNA Pretreatment Kit	Arraystar	AS-FS-005
rtStar First-Strand cDNA Synthesis Kit	Arraystar	AS-FS-003-02
Trizol	Thermo Fisher Scientific	15596018
cel-miR-39-3p mimic	Thermo Fisher Scientific	MC10956
On TARGETplus SMARTpool siTLR7	Dharmacon	L-004714-00-0005
On TARGETplus SMARTpool siTLR8	Dharmacon	L-004715-00-0005
Human anti-double stranded DNA (dsDNA) antibody (IgG) ELISA Kit ELISA Kit	CUSABIO	CSB-E04911h
Human IL-1 beta/IL-1F2 Quantikine ELISA Kit	R&D	DLB50
Human IL-8 Quantikine ELISA Kit	R&D	D8000C
Human IFNα ELISA kit	Thermo Fisher Scientific	LTMBMS216INST
ExoELISA-ULTRA Complete Kit	System Biosciences	EXEL-ULTRA-CD63-1

Supplementary Methods

Immunoblotting

The cells with different treatments were lysed in RIPA buffer (25 mM Tris-HCl pH 7.6, 150 mM NaCl, 1% NP-40, 1% sodium deoxycholate and 0.1% SDS) containing a protease inhibitor cocktail (Roche, Germany). Twenty micrograms of total protein from exosome lysate were loaded and separated on a standard sodium dodecyl sulfate (SDS)-polyacrylamide gel electrophoresis (PAGE) gel and transferred to a polyvinylidene difluoride (PVDF) membrane (Millipore, USA). The membranes were incubated with primary antibodies, followed by peroxidase-conjugated secondary antibodies. The results were detected using a charge-coupled device (CCD) camera-based imager (GE Healthcare Life Sciences, USA) after membrane incubation with enhanced chemiluminescence (ECL) substrates (Millipore, USA). The levels of specific protein were normalized to β-actin. The ImageJ software was used for image acquisition and densitometric analysis of the immunoblots. All results were obtained in three independent experiments and the data are presented as the mean ± SD. An unpaired, two-tailed Student's t-test was performed for between-group comparisons using GraphPad Prism software version 8. All results of densitometric analysis are presented in Additional file 2.

Transient transfection

Human neutrophils (1×10^6 cells) were transiently transfected with 30 nM TLR8

siRNA (Dharmacon, horizon, USA) or controls by using Lipofectamine RNAiMAX Transfection Reagent (Thermo Fisher Scientific, USA) according to the manufacturer's instructions, and incubated at 37°C for 24 h.

Flow cytometry

Platelet- or EV-specific surface marker staining for EVs was performed using the Exo-Flow exosome capture kit (System Biosciences, USA) according to the manufacturer's instructions. Briefly, purified EVs were mixed with immune-magnetic beads coated with anti-human CD63 monoclonal antibody (BioLegend, USA) and incubated overnight at 4°C. After incubation, the beads were washed using Bead Wash buffer (System Biosciences, USA). The EVs were incubated with the Exo-FITC exosome stain (System Biosciences, USA) for exosome detection, AF647-conjugated anti-CD41 monoclonal antibody for platelet-surface marker detection (BD Biosciences, USA), respectively; then, they were examined by flow cytometry (FACSCanto II, BD Biosciences, USA). AF647-conjugated IgG1 (BD Biosciences, USA) was used as an isotype control. All data were analyzed using the CellQuest (BD Biosciences) or FlowJo software.

Quantification of ROS production

The levels of cytosolic ROS were analyzed by using fluorescent dye dihydrorhodamine (DHR) 123 (Thermo Fisher Scientific) staining and quantified by

flow cytometry. Data of flow cytometry were analyzed by the CellQuest software and expressed as the mean fluorescence intensity (MFI) of cytosolic ROS.

Supplementary Table 1 The sequence of tsRNAs primer sets, mimic, and inhibitor used in this study.

Name	Sequence	Ref.
tRF Universal Primer R	5′-AGTGCAGGGTCCGAGGTATT-3′	[9]
tRF-His-GTG-1_F	5′-CGCGGCCGGACGAAGG-3′	[9]
tRF-chrM.Pro-TGG _F	5′-CGCGCGCGCGAAGACC-3′	[9]
tRF-Val-AAC-1-M7_F	5′-CGCGCGCGCGGCCGAG-3′	[9]
tRF-His-GTG-1 mimic	5′-GCCGUGAUCGUAUAGUGGUUAGUACUCU-3′ (2'-O-Methyl modification)	[9]
tRF-His-GTG-1 inhibitor	5′-AACCACUAUACGAUCACGGC-3′ (2'-O-Methyl modification)	[9]
tRF mimic negative control	5′-UUGUACUACACAAAAGUACUG-3′ (2'-O-Methyl modification)	[32]
tRF inhibitor negative control	5′-CAGUACUUUUGUGUAGUACAA-3′ (2'-O-Methyl modification)	[32]

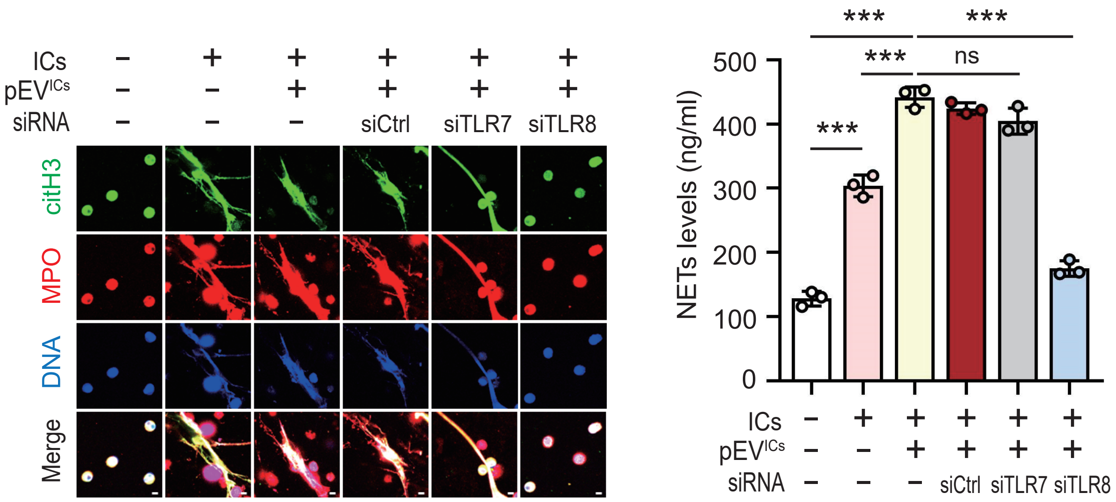

Supplementary Figure S1. (A) SLE immune complex (ICs)-primed platelet-derived extracellular vesicles (pEV^{ICs}) enhanced ICs-induced NETs formation, and this effect was declined in TLR8 knock-down cells. The scale bar in the IFA image represents 5μm. All experiments were performed in triplicate, and data were presented as mean ± SD. ***$P < 0.005$. ns, non-significant.

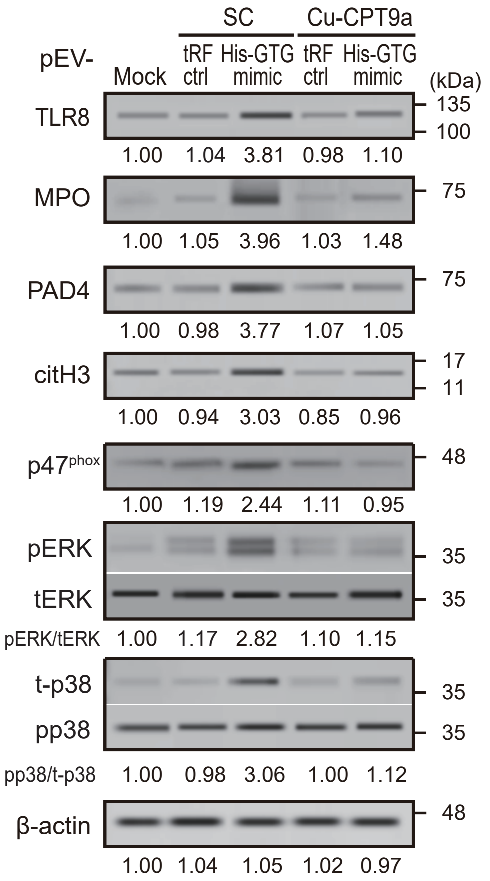

Supplementary Figure S2. Human neutrophils were treated with pEVs that carried-tRF-His-GTG-1 mimic or mimic control in the presence or absence of TLR8 inhibitor Cu-CPT9a for 24h. The expression of intracellular TLR8 and NETs-associated proteins was analyzed by using immunoblotting. Immunoblotting bands from β-actin were densitometrically measured by ImageJ to determine the lane normalization factor for samples. The image shown is from a single experiment that is representative of at least three separate experiments.

A

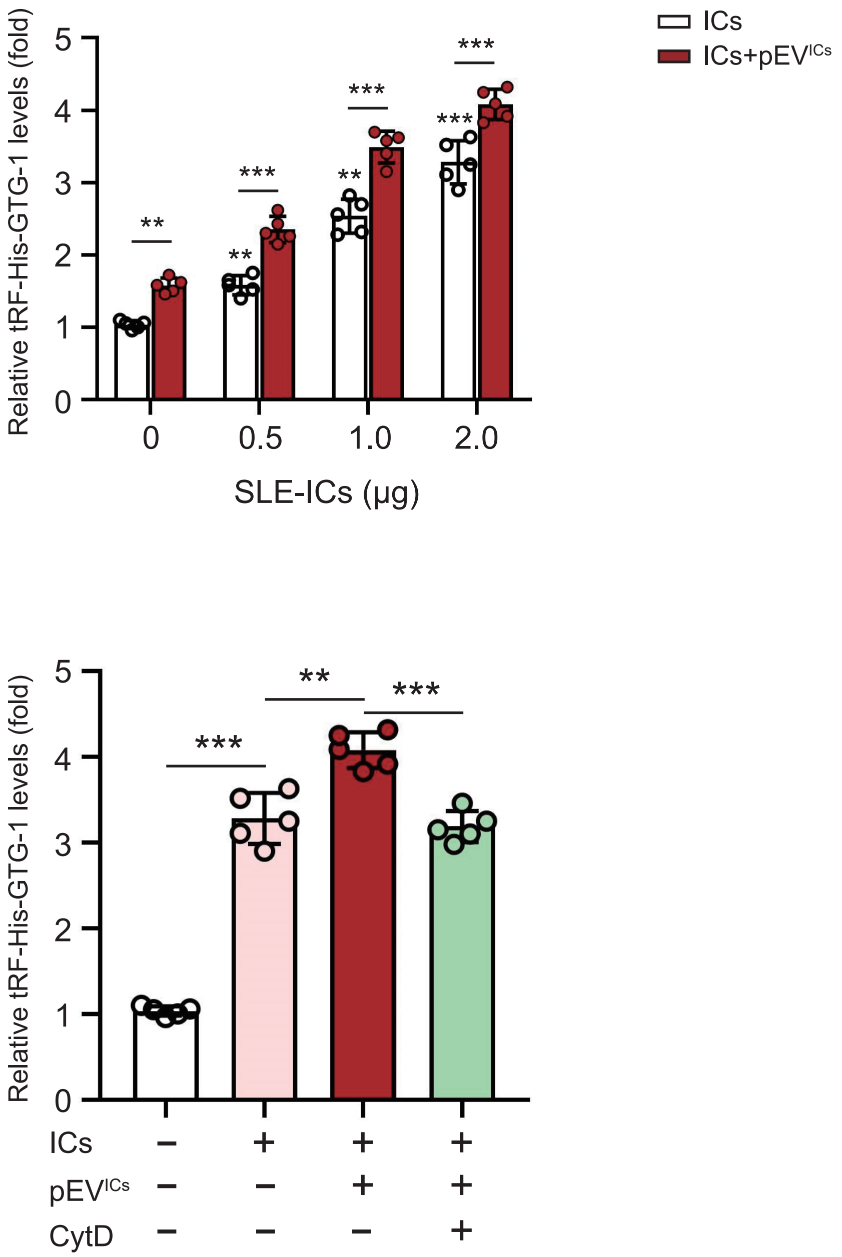

Supplementary Figure S3. (A) Elevated levels of tRF-His-GTG-1 in normal neutrophils with SLE patients-derived immune complexes (ICs) treatment in a dose-depentdent manner. (B) SLE patients-ICs primed platelets-derived extracellular vesicles (pEV^{ICs}) enhanced ICs-induced tRF-His-GTG-1 expression in neutrophils through uptake. Data are presented as the mean ± SD. $^{**}P < 0.01$, $^{***}P < 0.005$.
