# Supplementary material for: tRF-His-GTG-1 enhances NETs formation and interferon-α production in lupus by extracellular vesicle
Source: Cell Commun Signal. 2024 Jul 7;22:354. doi: 10.1186/s12964-024-01730-7 (PMC11229248; doi:10.1186/s12964-024-01730-7)
Supplement: Supplementary file 2 — Supplementary Material 2 [file 12964_2024_1730_MOESM2_ESM.pdf]

Fig. 1B

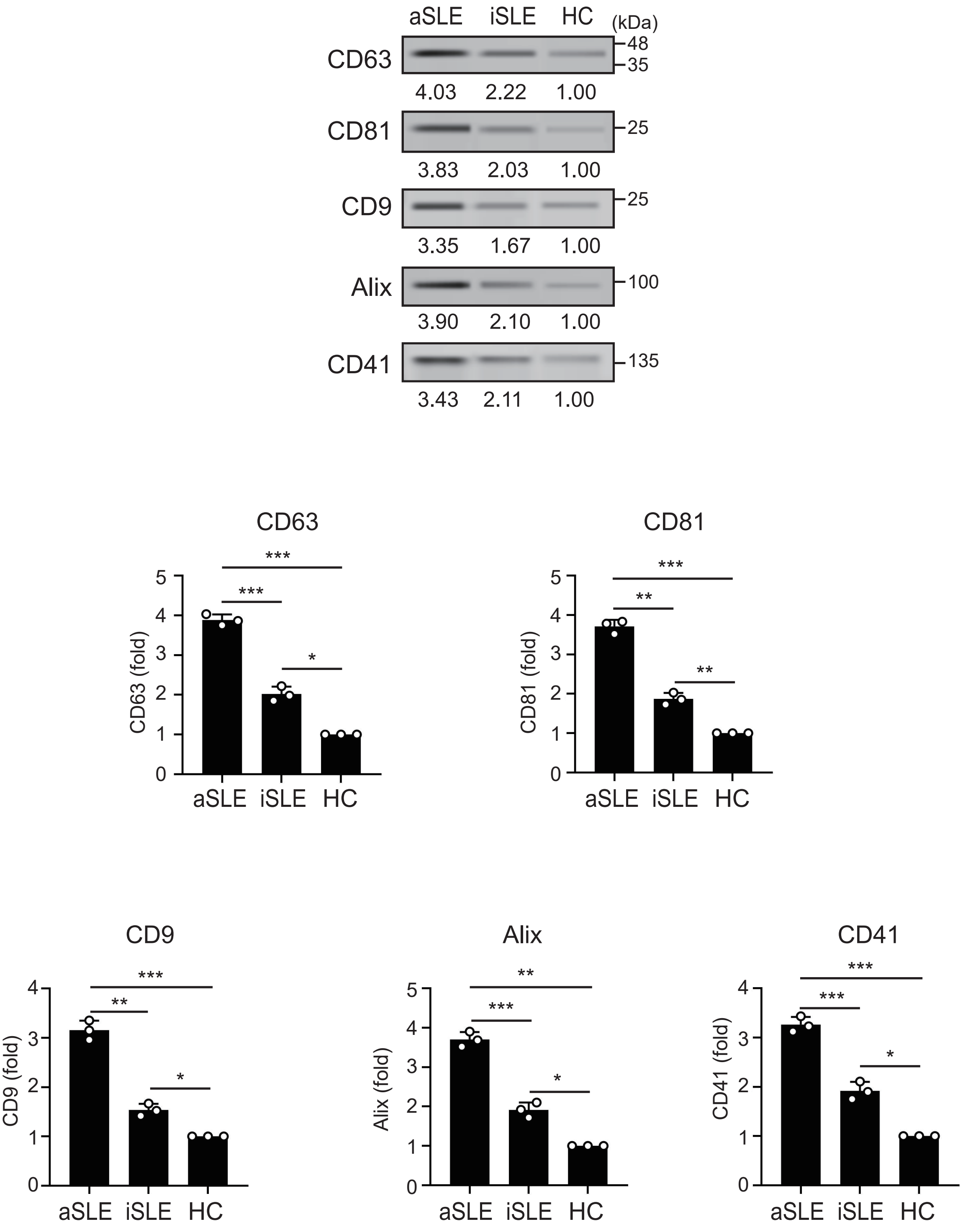
aSLE iSLE HC
(kDa)
CD63
48
35
4.03 2.22 1.00
CD81
25
3.83 2.03 1.00
CD9
25
3.35 1.67 1.00
Alix
100
3.90 2.10 1.00
CD41
135
3.43 2.11 1.00
CD63
CD63 (fold)
CD81
CD81 (fold)
CD9
CD9 (fold)
Alix
Alix (fold)
CD41
CD41 (fold)
aSLE iSLE HC

Fig. 1I

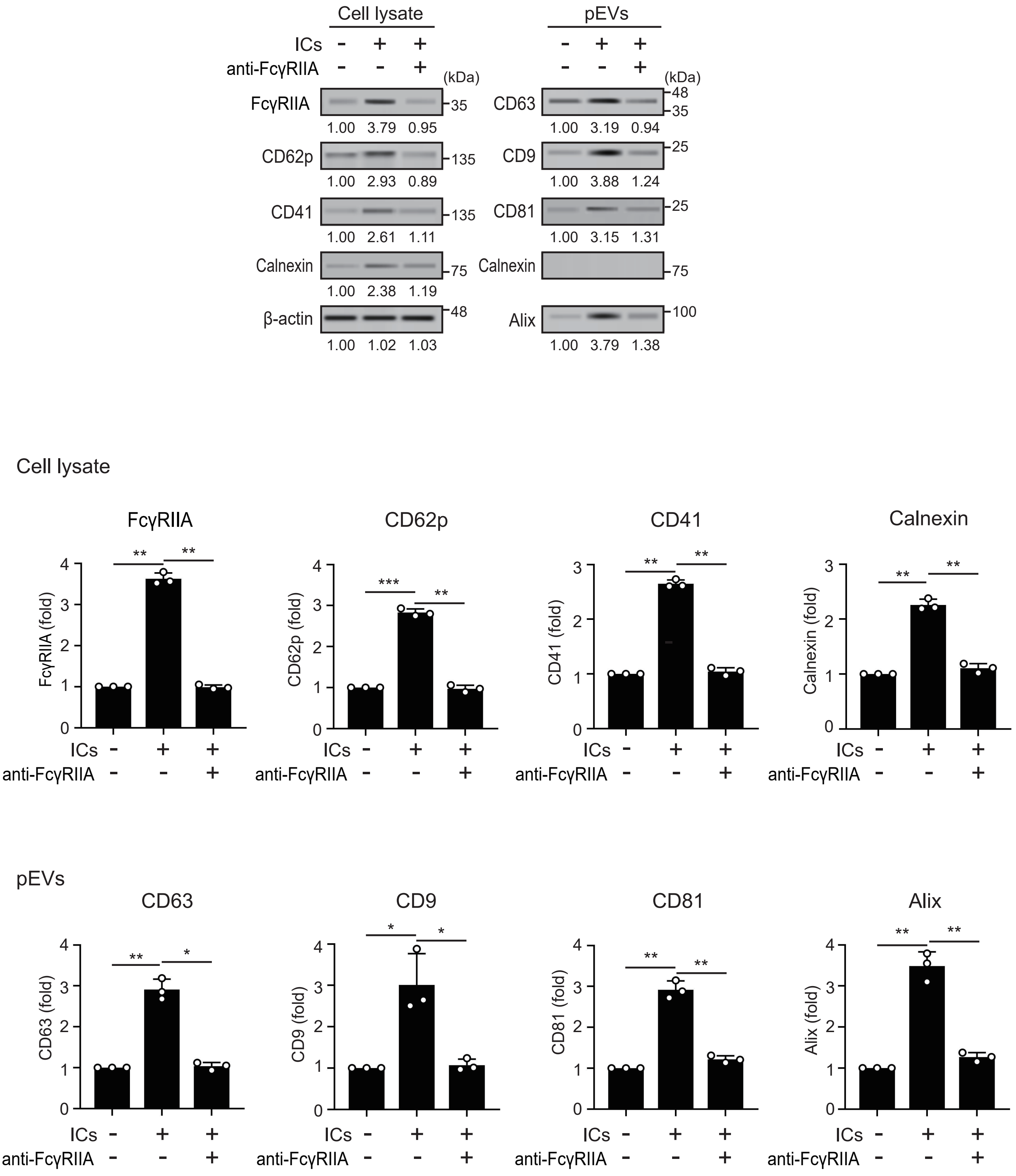
Cell lysate
pEVs
ICs - + +
anti-FcγRIIA - - +
(kDa)
FcγRIIA
35
1.00 3.79 0.95
CD62p
135
1.00 2.93 0.89
CD41
135
1.00 2.61 1.11
Calnexin
75
1.00 2.38 1.19
β-actin
48
1.00 1.02 1.03
CD63
48
35
1.00 3.19 0.94
CD9
25
1.00 3.88 1.24
CD81
25
1.00 3.15 1.31
Calnexin
75
Alix
100
1.00 3.79 1.38
Cell lysate
FcγRIIA
FcγRIIA (fold)
CD62p
CD62p (fold)
CD41
CD41 (fold)
Calnexin
Calnexin (fold)
pEVs
CD63
CD63 (fold)
CD9
CD9 (fold)
CD81
CD81 (fold)
Alix
Alix (fold)
ICs - + +
anti-FcγRIIA - - +

Fig. 2E

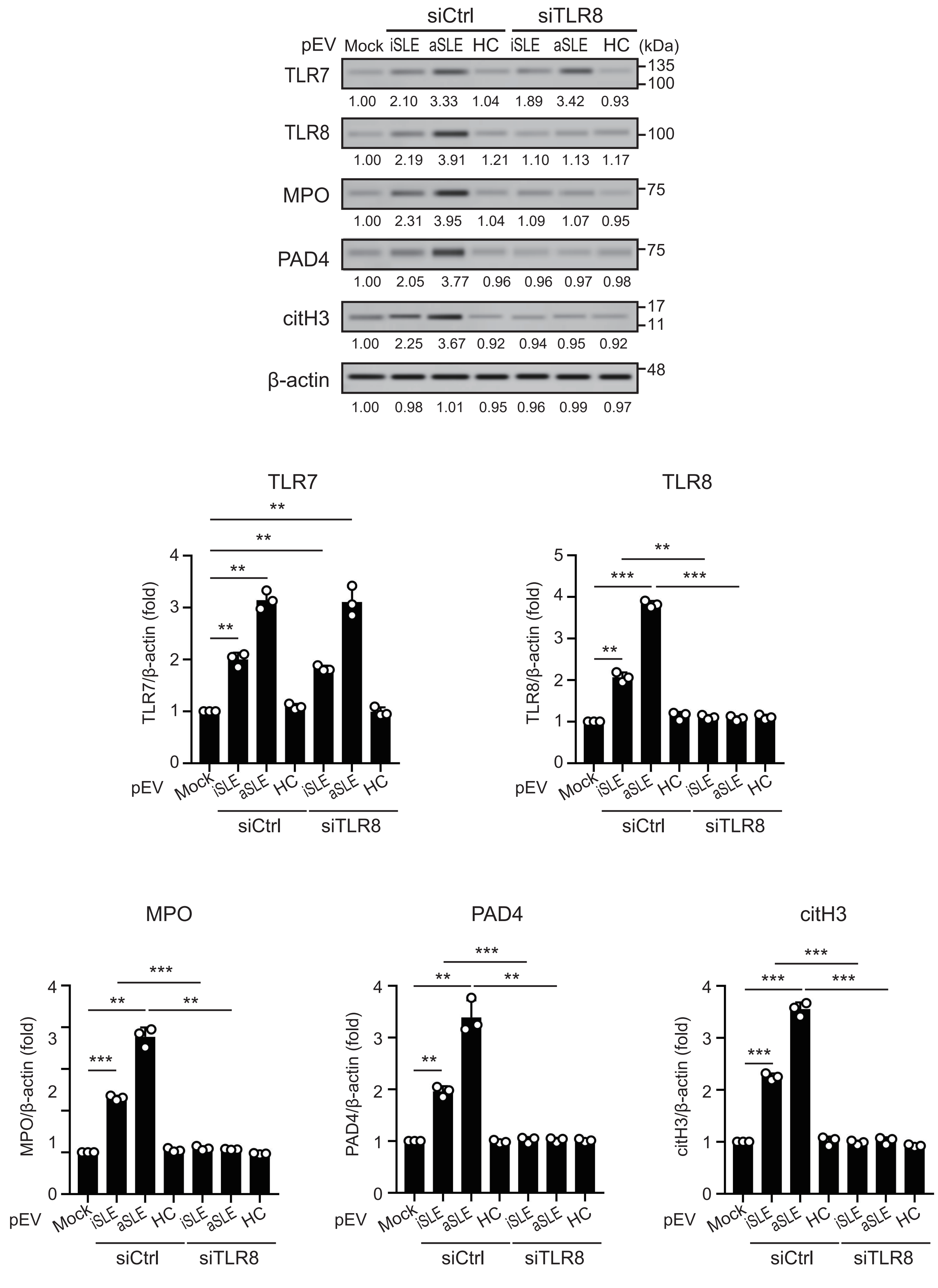
siCtrl
siTLR8
pEV Mock iSLE aSLE HC iSLE aSLE HC (kDa)
TLR7
135
100
1.00 2.10 3.33 1.04 1.89 3.42 0.93
TLR8
100
1.00 2.19 3.91 1.21 1.10 1.13 1.17
MPO
75
1.00 2.31 3.95 1.04 1.09 1.07 0.95
PAD4
75
1.00 2.05 3.77 0.96 0.96 0.97 0.98
citH3
17
11
1.00 2.25 3.67 0.92 0.94 0.95 0.92
β-actin
48
1.00 0.98 1.01 0.95 0.96 0.99 0.97
TLR7
TLR7/β-actin (fold)
TLR8
TLR8/β-actin (fold)
MPO
MPO/β-actin (fold)
PAD4
PAD4/β-actin (fold)
citH3
citH3/β-actin (fold)
pEV Mock iSLE aSLE HC iSLE aSLE HC
siCtrl
siTLR8

Fig. 2H

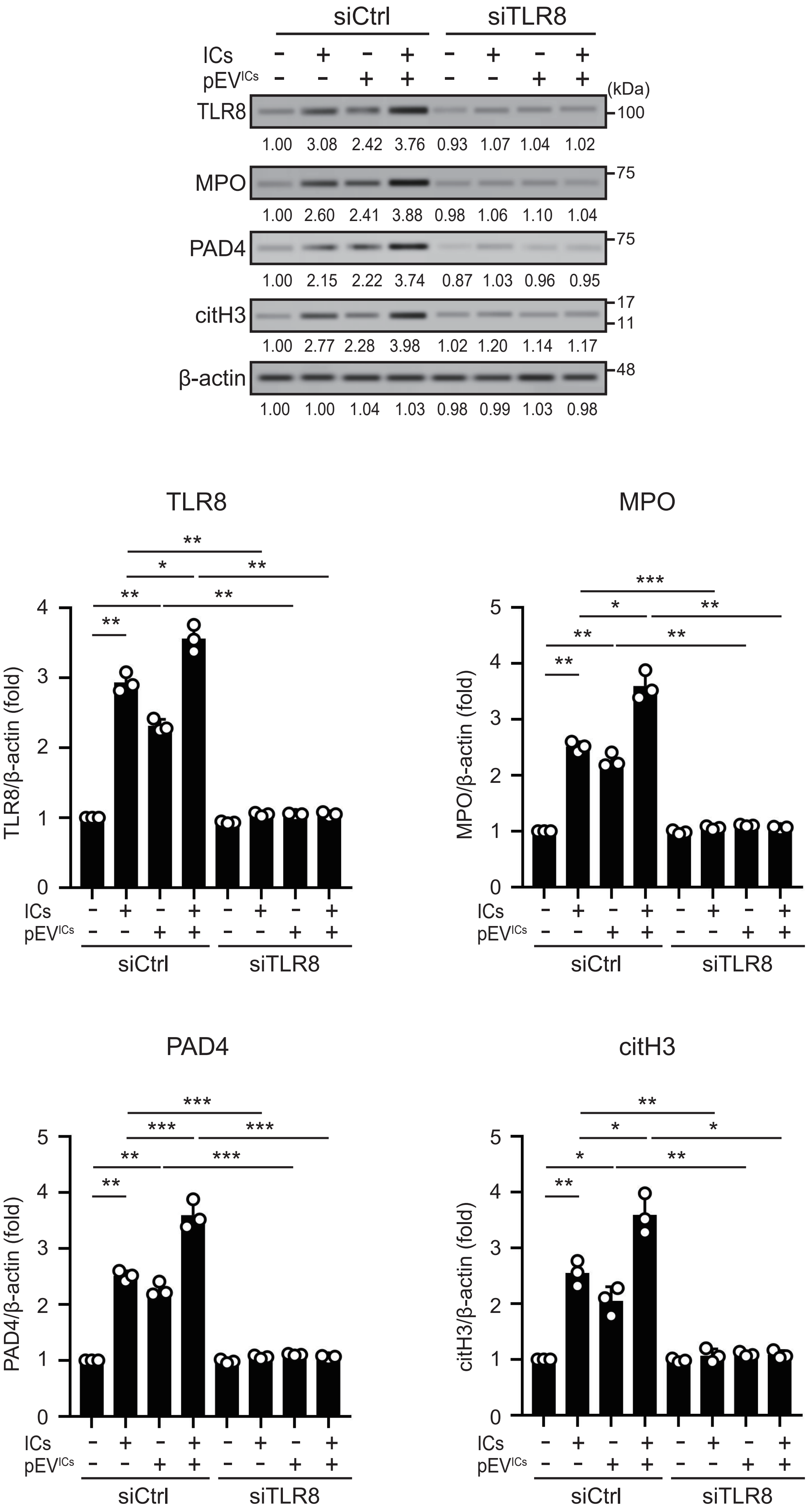

siCtrl
siTLR8
ICs
pEV^ICs
(kDa)
TLR8
100
1.00 3.08 2.42 3.76 0.93 1.07 1.04 1.02
MPO
75
1.00 2.60 2.41 3.88 0.98 1.06 1.10 1.04
PAD4
75
1.00 2.15 2.22 3.74 0.87 1.03 0.96 0.95
citH3
17
11
1.00 2.77 2.28 3.98 1.02 1.20 1.14 1.17
β-actin
48
1.00 1.00 1.04 1.03 0.98 0.99 1.03 0.98
TLR8
TLR8/β-actin (fold)
MPO
MPO/β-actin (fold)
PAD4
PAD4/β-actin (fold)
citH3
citH3/β-actin (fold)

Fig. 3H

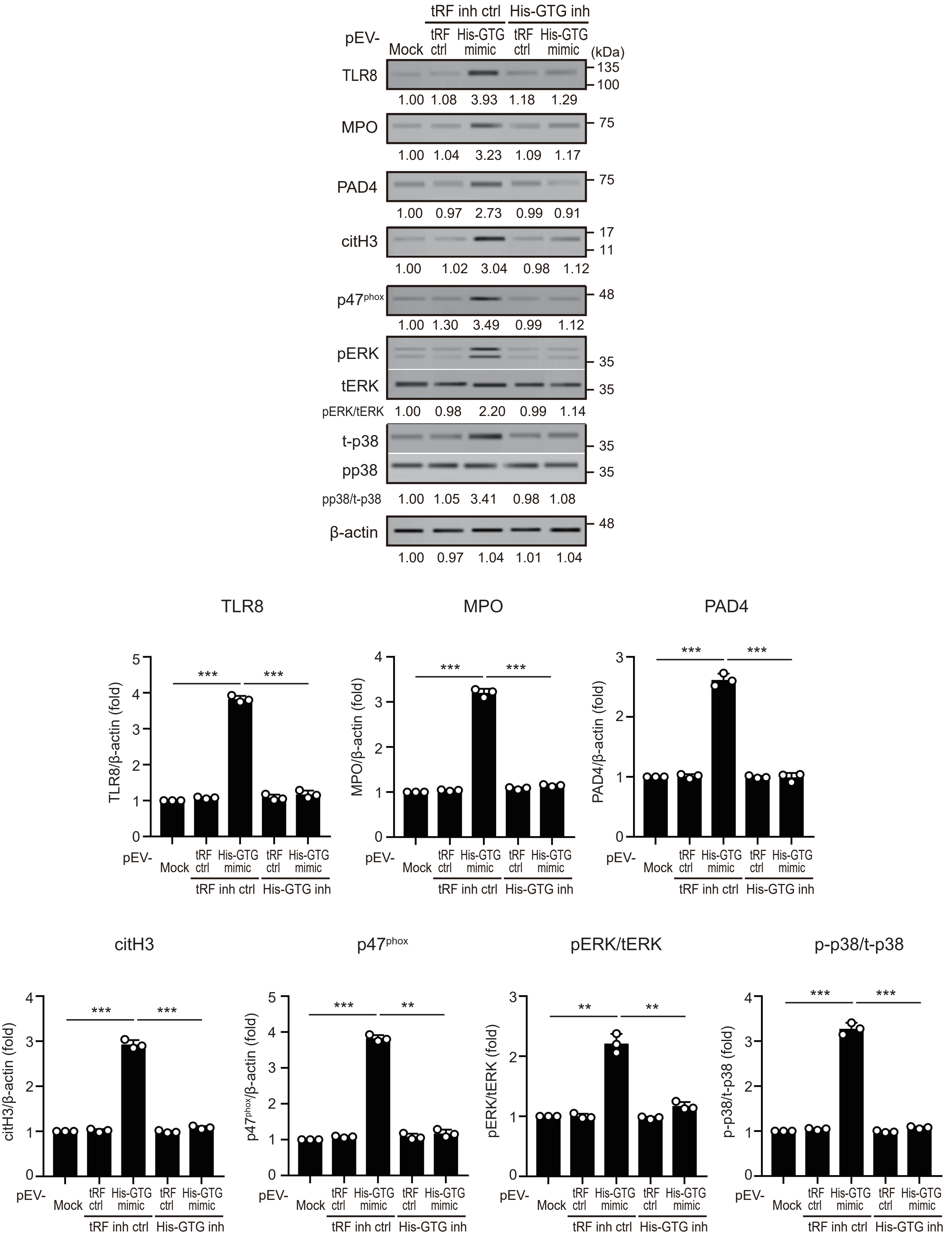

tRF inh ctrl
His-GTG inh
pEV-
Mock
tRF ctrl
His-GTG mimic
tRF ctrl
His-GTG mimic
(kDa)
TLR8
135
100
1.00 1.08 3.93 1.18 1.29
MPO
75
1.00 1.04 3.23 1.09 1.17
PAD4
75
1.00 0.97 2.73 0.99 0.91
citH3
17
11
1.00 1.02 3.04 0.98 1.12
p47phox
48
1.00 1.30 3.49 0.99 1.12
pERK
35
tERK
35
pERK/tERK 1.00 0.98 2.20 0.99 1.14
t-p38
35
pp38
35
pp38/t-p38 1.00 1.05 3.41 0.98 1.08
β-actin
48
1.00 0.97 1.04 1.01 1.04
TLR8
TLR8/β-actin (fold)
MPO
MPO/β-actin (fold)
PAD4
PAD4/β-actin (fold)
citH3
citH3/β-actin (fold)
p47phox
p47phox/β-actin (fold)
pERK/tERK
pERK/tERK (fold)
p-p38/t-p38
p-p38/t-p38 (fold)
***
**

Fig. 4D

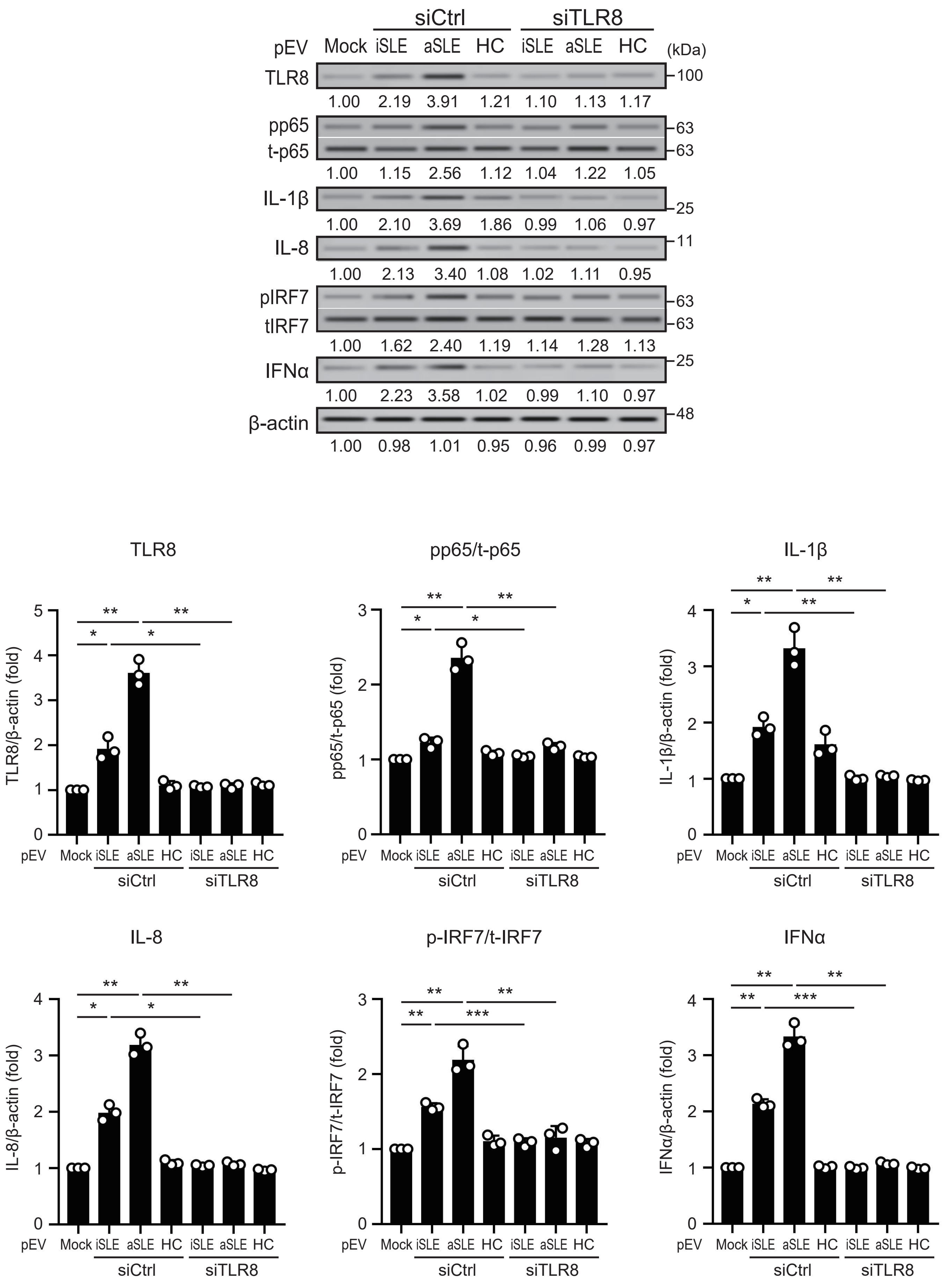

siCtrl
siTLR8
pEV
Mock
iSLE
aSLE
HC
(kDa)
TLR8
100
1.00 2.19 3.91 1.21 1.10 1.13 1.17
pp65
t-p65
63
63
1.00 1.15 2.56 1.12 1.04 1.22 1.05
IL-1β
25
1.00 2.10 3.69 1.86 0.99 1.06 0.97
IL-8
11
1.00 2.13 3.40 1.08 1.02 1.11 0.95
pIRF7
tIRF7
63
63
1.00 1.62 2.40 1.19 1.14 1.28 1.13
IFNα
25
1.00 2.23 3.58 1.02 0.99 1.10 0.97
β-actin
48
1.00 0.98 1.01 0.95 0.96 0.99 0.97
TLR8
TLR8/β-actin (fold)
pp65/t-p65
pp65/t-p65 (fold)
IL-1β
IL-1β/β-actin (fold)
IL-8
IL-8/β-actin (fold)
p-IRF7/t-IRF7
p-IRF7/t-IRF7 (fold)
IFNα
IFNα/β-actin (fold)

Fig. 4G

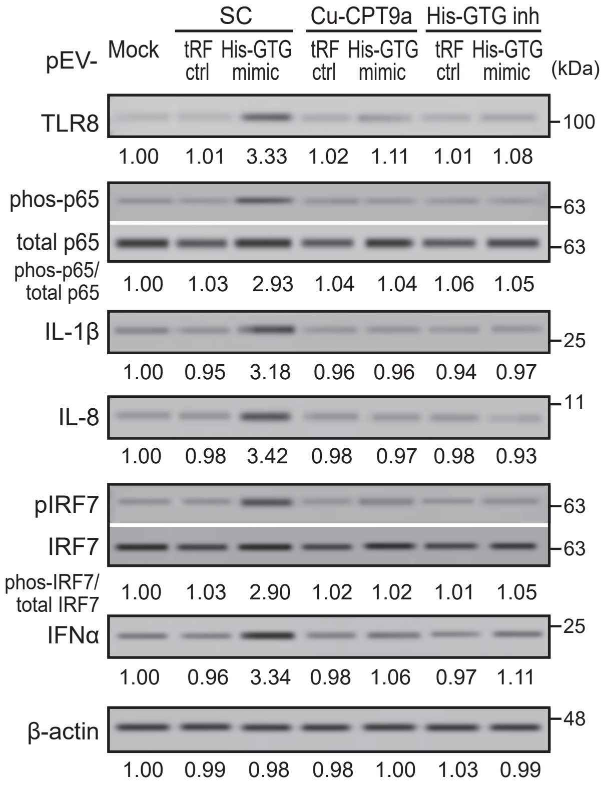

SC
Cu-CPT9a
His-GTG inh
pEV-
Mock
tRF ctrl
His-GTG mimic
(kDa)
TLR8
100
1.00 1.01 3.33 1.02 1.11 1.01 1.08
phos-p65
63
total p65
63
phos-p65/ total p65
1.00 1.03 2.93 1.04 1.04 1.06 1.05
IL-1β
25
1.00 0.95 3.18 0.96 0.96 0.94 0.97
IL-8
11
1.00 0.98 3.42 0.98 0.97 0.98 0.93
pIRF7
63
IRF7
63
phos-IRF7/ total IRF7
1.00 1.03 2.90 1.02 1.02 1.01 1.05
IFNα
25
1.00 0.96 3.34 0.98 1.06 0.97 1.11
β-actin
48
1.00 0.99 0.98 0.98 1.00 1.03 0.99

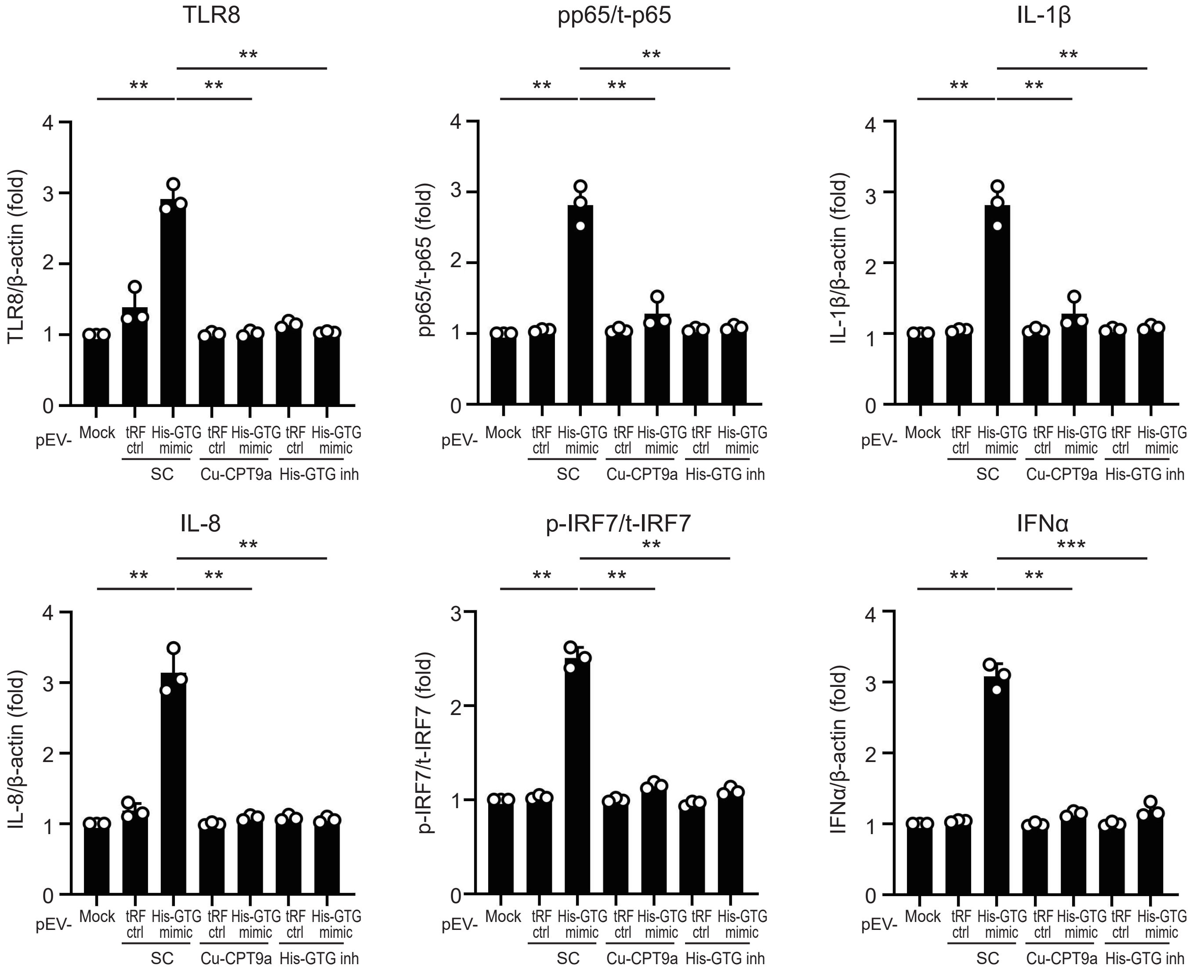

TLR8
TLR8/β-actin (fold)
pp65/t-p65
pp65/t-p65 (fold)
IL-1β
IL-1β/β-actin (fold)
IL-8
IL-8/β-actin (fold)
p-IRF7/t-IRF7
p-IRF7/t-IRF7 (fold)
IFNα
IFNα/β-actin (fold)
**
***
pEV-
Mock
tRF ctrl
His-GTG mimic
SC
Cu-CPT9a
His-GTG inh

Fig. 4I

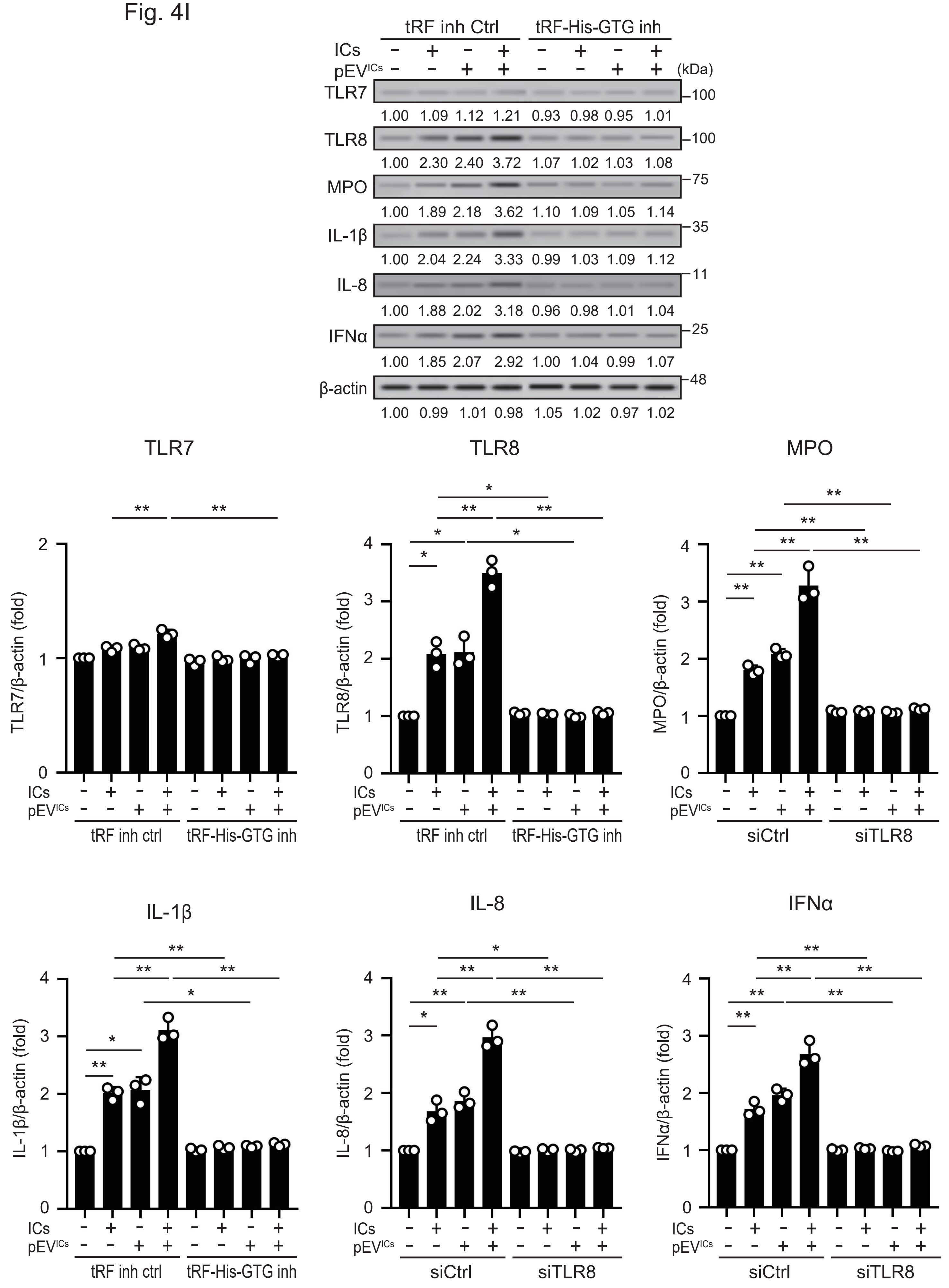
tRF inh Ctrl
tRF-His-GTG inh
ICs
pEV^ICs
(kDa)
TLR7
1.00 1.09 1.12 1.21 0.93 0.98 0.95 1.01
100
TLR8
1.00 2.30 2.40 3.72 1.07 1.02 1.03 1.08
100
MPO
1.00 1.89 2.18 3.62 1.10 1.09 1.05 1.14
75
IL-1β
1.00 2.04 2.24 3.33 0.99 1.03 1.09 1.12
35
IL-8
1.00 1.88 2.02 3.18 0.96 0.98 1.01 1.04
11
IFNα
1.00 1.85 2.07 2.92 1.00 1.04 0.99 1.07
25
β-actin
1.00 0.99 1.01 0.98 1.05 1.02 0.97 1.02
48
TLR7
TLR7/β-actin (fold)
tRF inh ctrl
tRF-His-GTG inh
TLR8
TLR8/β-actin (fold)
tRF inh ctrl
tRF-His-GTG inh
MPO
MPO/β-actin (fold)
siCtrl
siTLR8
IL-1β
IL-1β/β-actin (fold)
tRF inh ctrl
tRF-His-GTG inh
IL-8
IL-8/β-actin (fold)
siCtrl
siTLR8
IFNα
IFNα/β-actin (fold)
siCtrl
siTLR8

Fig. S2

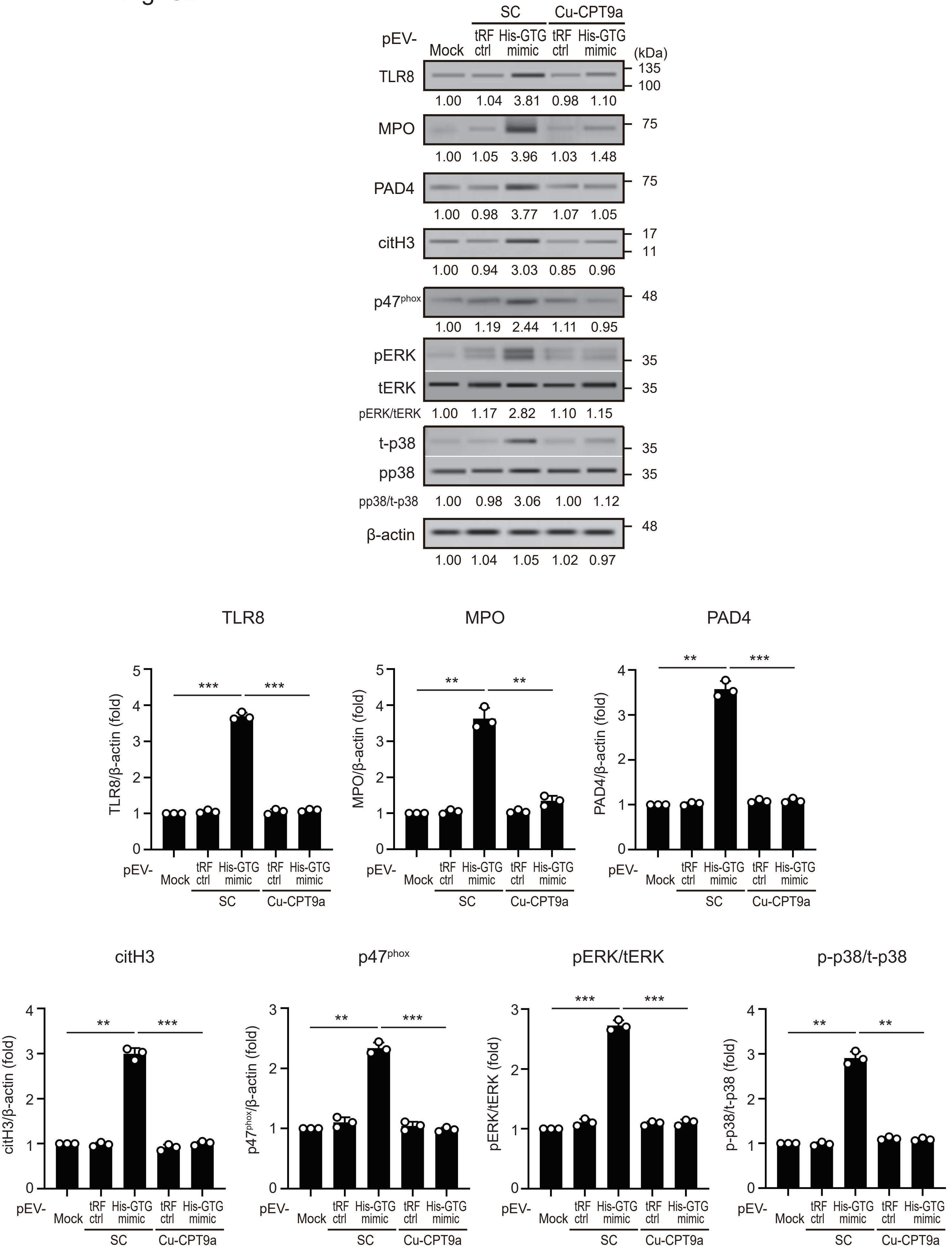
SC
Cu-CPT9a
pEV-
Mock
tRF ctrl
His-GTG mimic
tRF ctrl
His-GTG mimic
(kDa)
TLR8
135
100
1.00 1.04 3.81 0.98 1.10
MPO
75
1.00 1.05 3.96 1.03 1.48
PAD4
75
1.00 0.98 3.77 1.07 1.05
citH3
17
11
1.00 0.94 3.03 0.85 0.96
p47phox
48
1.00 1.19 2.44 1.11 0.95
pERK
35
tERK
35
pERK/tERK 1.00 1.17 2.82 1.10 1.15
t-p38
35
pp38
35
pp38/t-p38 1.00 0.98 3.06 1.00 1.12
β-actin
48
1.00 1.04 1.05 1.02 0.97
TLR8
TLR8/β-actin (fold)
MPO
MPO/β-actin (fold)
PAD4
PAD4/β-actin (fold)
citH3
citH3/β-actin (fold)
p47phox
p47phox/β-actin (fold)
pERK/tERK
pERK/tERK (fold)
p-p38/t-p38
p-p38/t-p38 (fold)
**
***
