# Supplementary material for: tRF-His-GTG-1 enhances NETs formation and interferon-α production in lupus by extracellular vesicle
Source: Cell Commun Signal. 2024 Jul 7;22:354. doi: 10.1186/s12964-024-01730-7 (PMC11229248; doi:10.1186/s12964-024-01730-7)
Supplement: Supplementary file 3 — Supplementary Material 3 [file 12964_2024_1730_MOESM3_ESM.pdf]

Fig. 1B

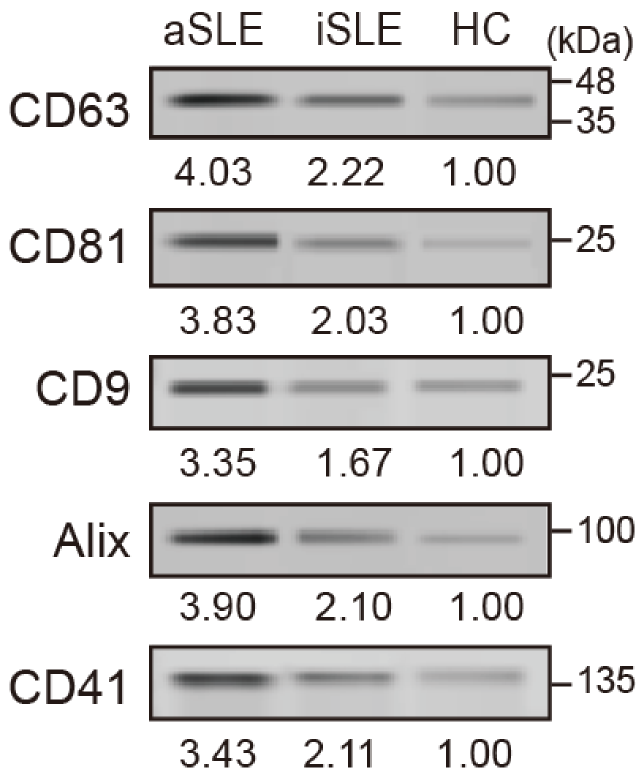

aSLE
iSLE
HC
(kDa)
CD63
48
35
4.03
2.22
1.00
CD81
25
3.83
2.03
1.00
CD9
25
3.35
1.67
1.00
Alix
100
3.90
2.10
1.00
CD41
135
3.43
2.11
1.00

CD63 (43 kDa) & CD81 (25 kDa)

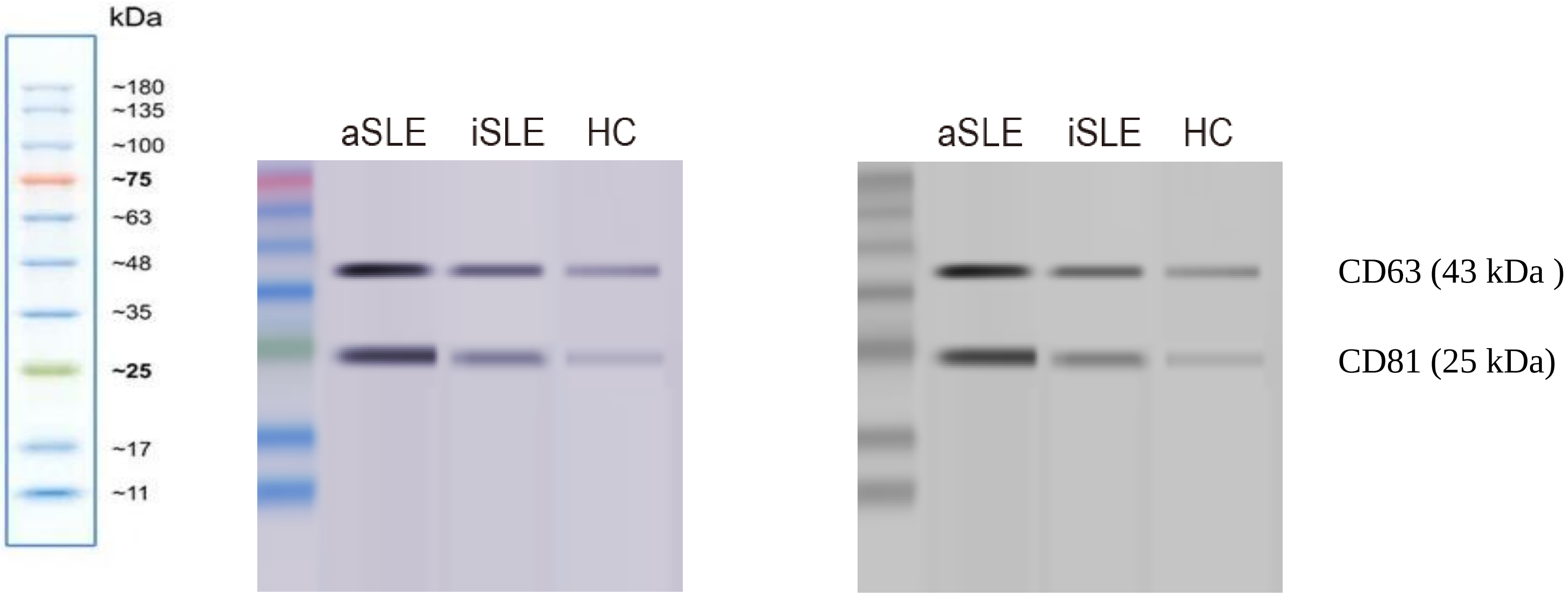

CD41(137 kDa) & CD9 (24 kDa)

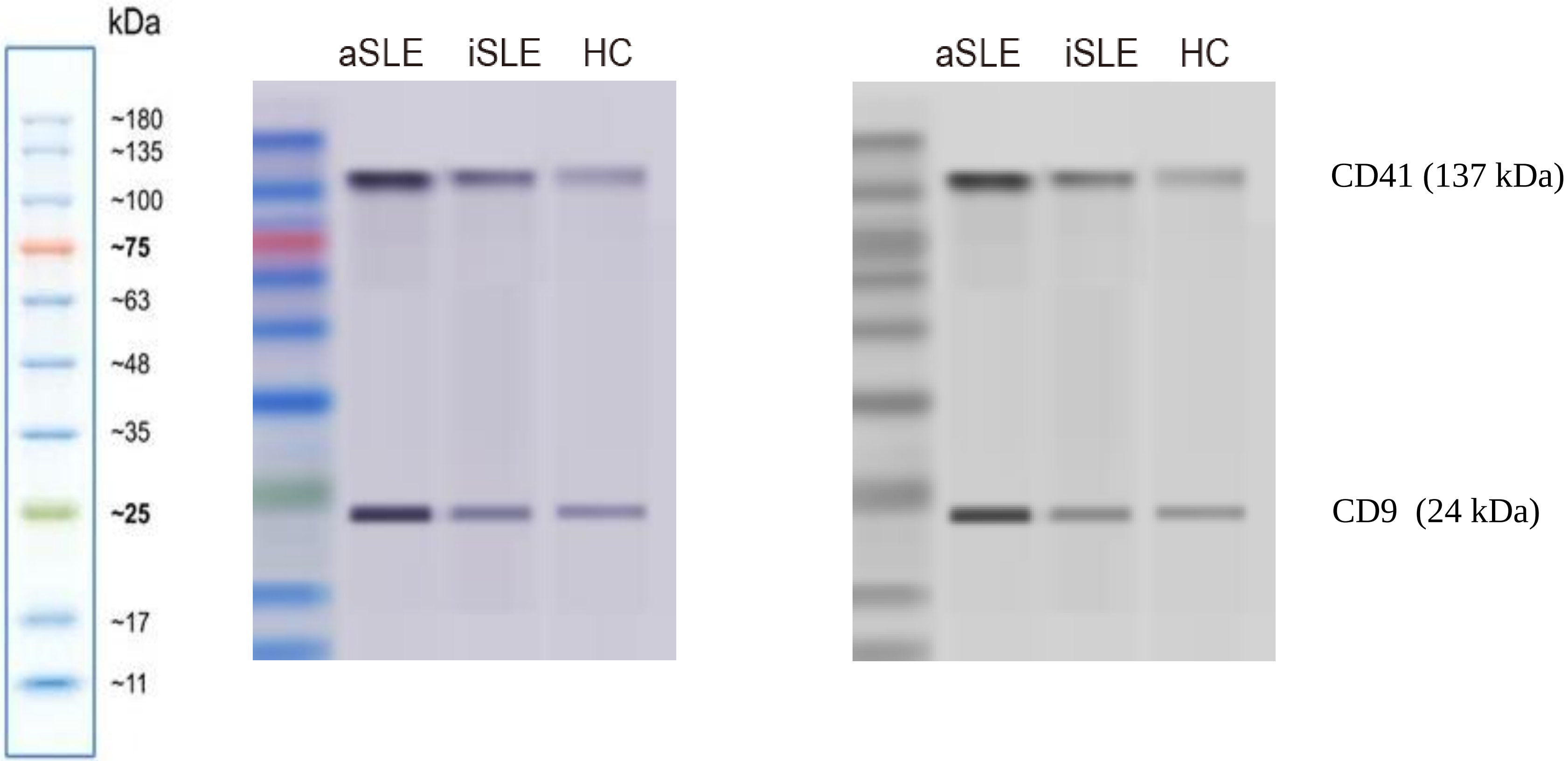

Alix (95 kda)

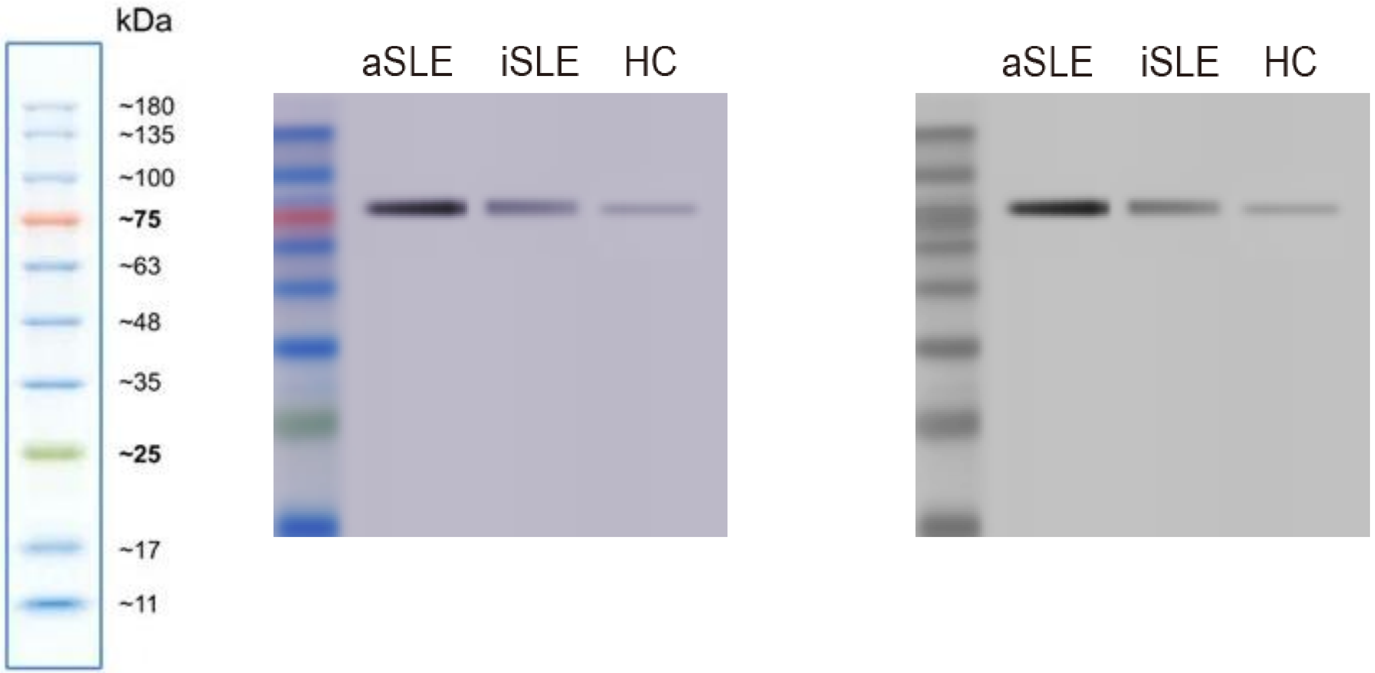

Alix (95 kda)

Fig. 1I

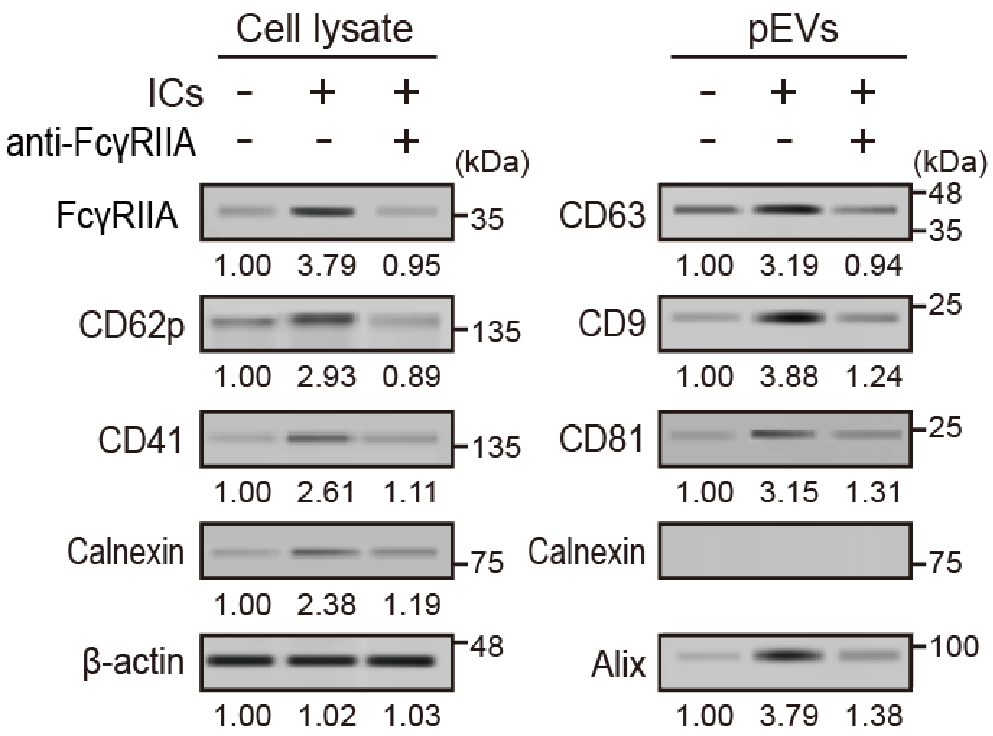

Cell lysate
pEVs
ICs - + +
anti-FcγRIIA - - +
(kDa)
FcγRIIA
35
1.00 3.79 0.95
CD62p
135
1.00 2.93 0.89
CD41
135
1.00 2.61 1.11
Calnexin
75
1.00 2.38 1.19
β-actin
48
1.00 1.02 1.03
- + +
- - +
(kDa)
CD63
48
35
1.00 3.19 0.94
CD9
25
1.00 3.88 1.24
CD81
25
1.00 3.15 1.31
Calnexin
75
Alix
100
1.00 3.79 1.38

FcγRIIA (35 kDa)

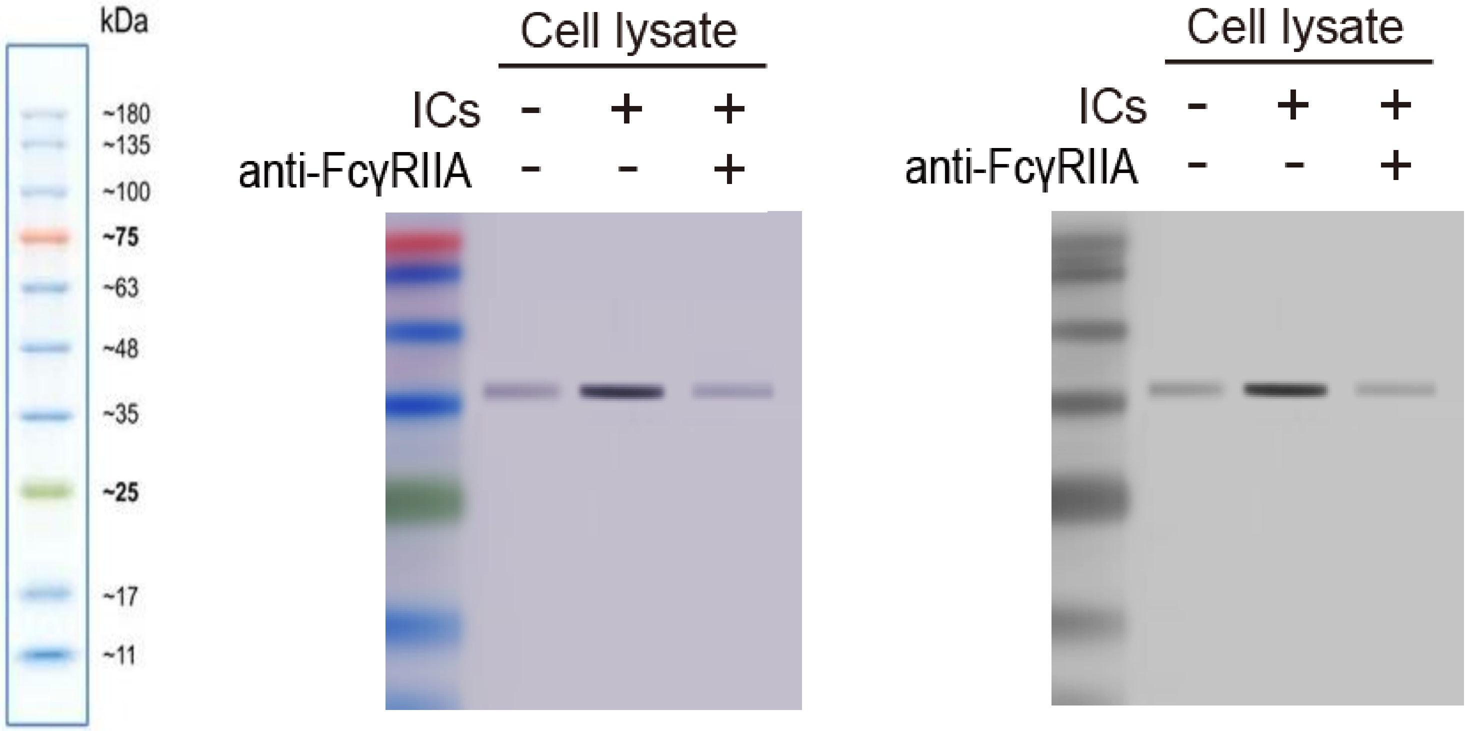

FcγRIIA (35 kDa)

CD62p (140 kda)

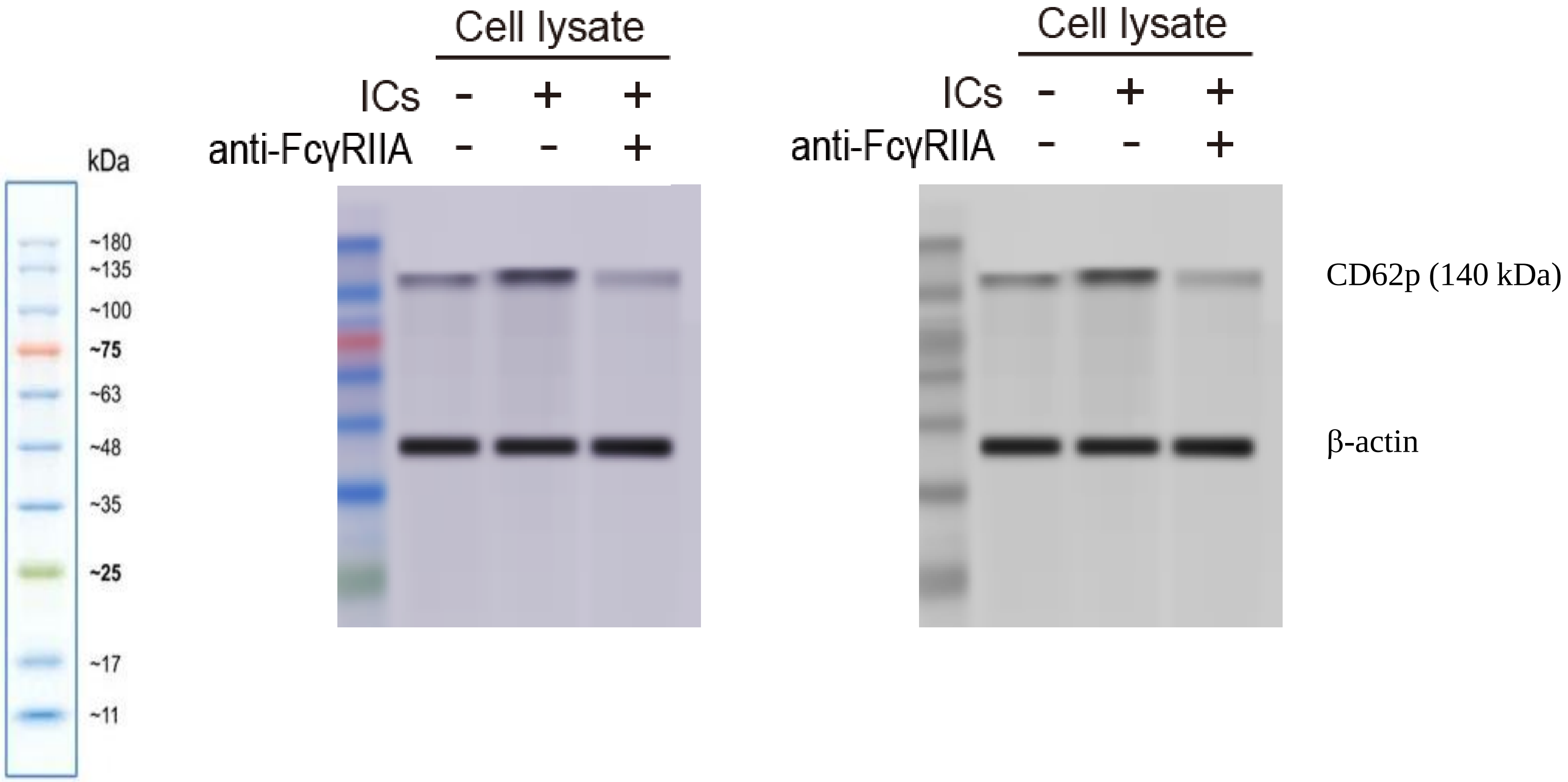

CD41(137 kda)

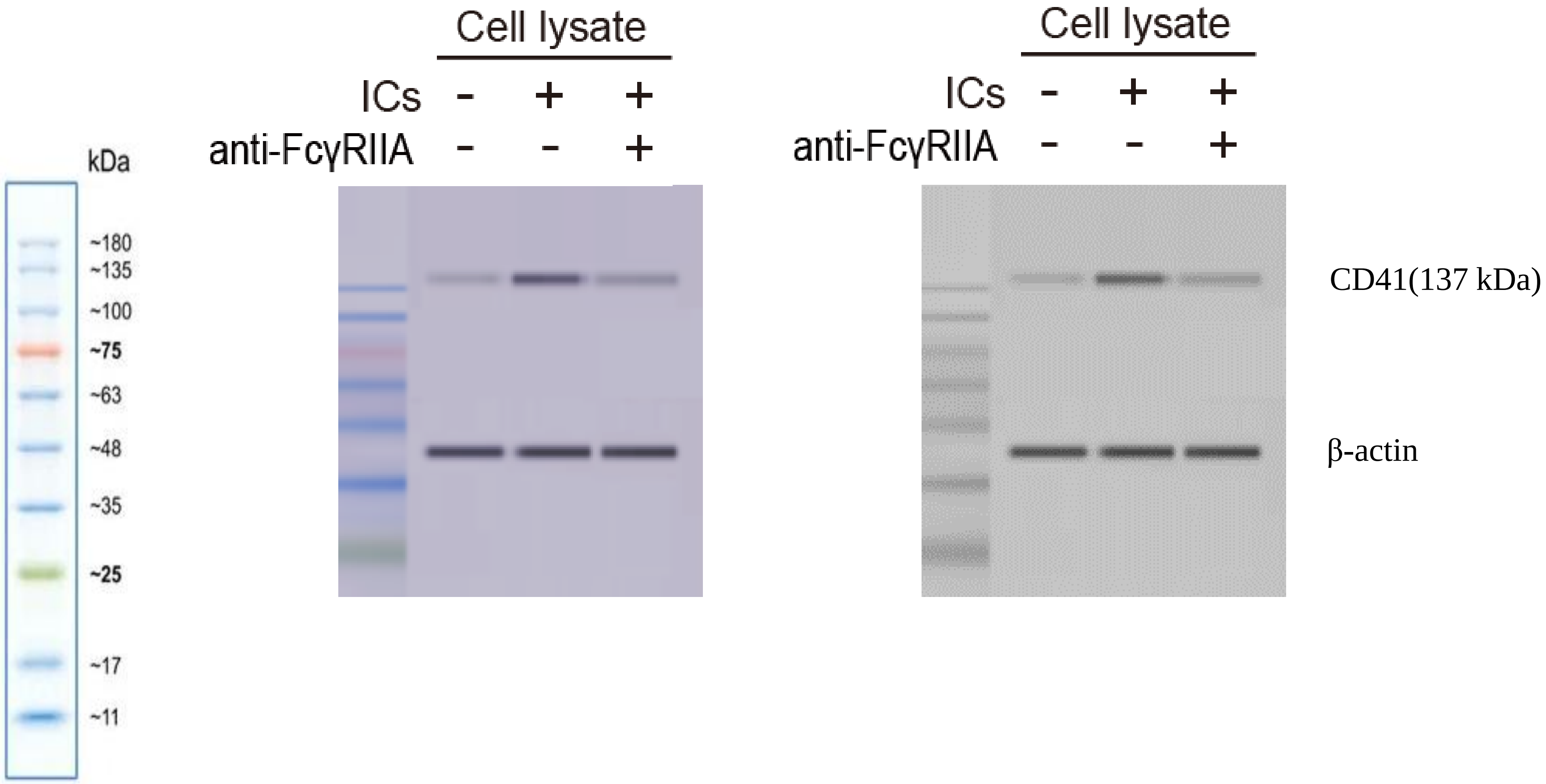
kDa
~180
~135
~100
~75
~63
~48
~35
~25
~17
~11
Cell lysate
ICs - + +
anti-FcγRIIA - - +
Cell lysate
ICs - + +
anti-FcγRIIA - - +
CD41(137 kDa)
β-actin

Calnexin (90 kda)

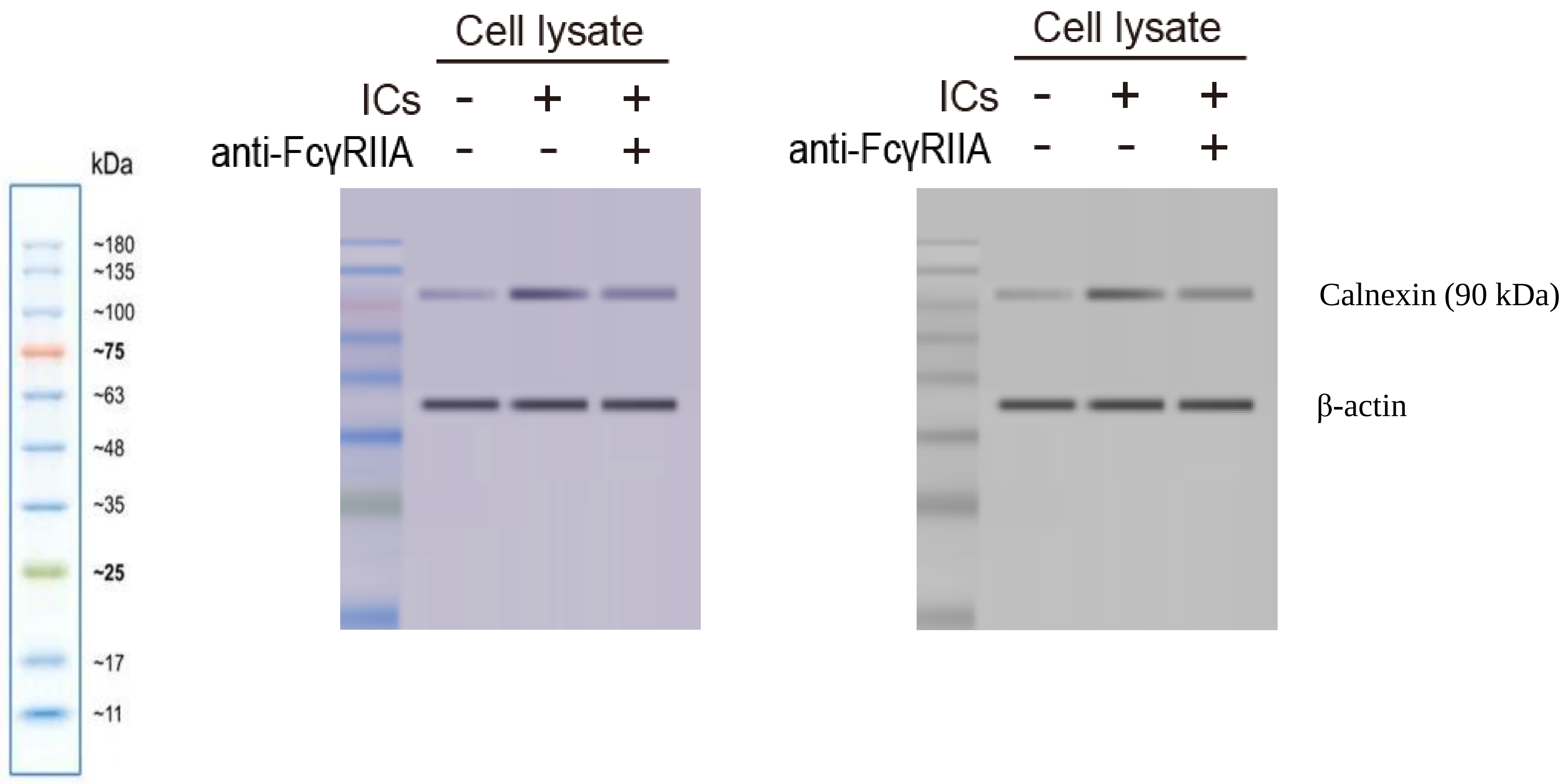

CD41(137 kda) & CD63 (43 kDa)

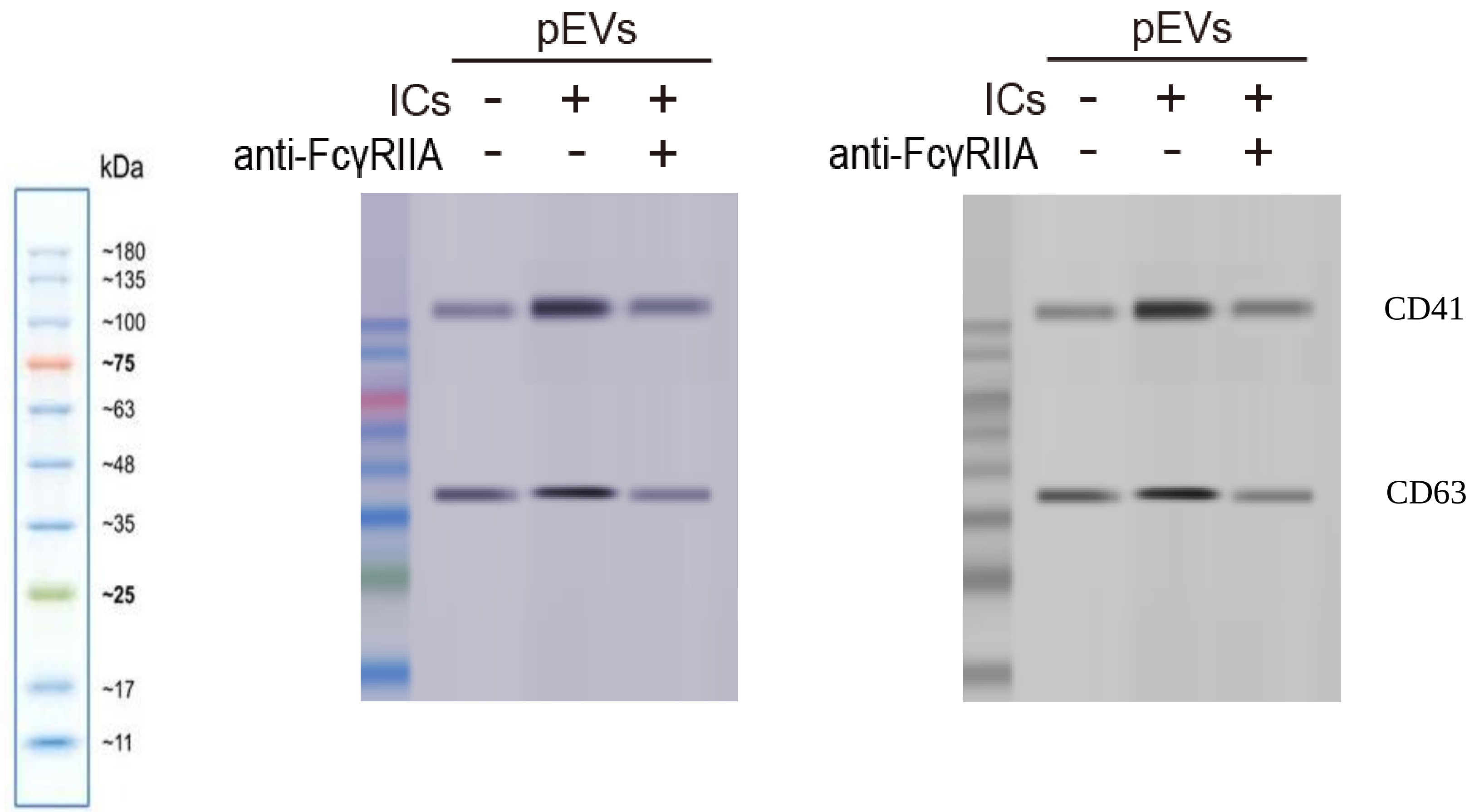

CD81 (25 kDa)

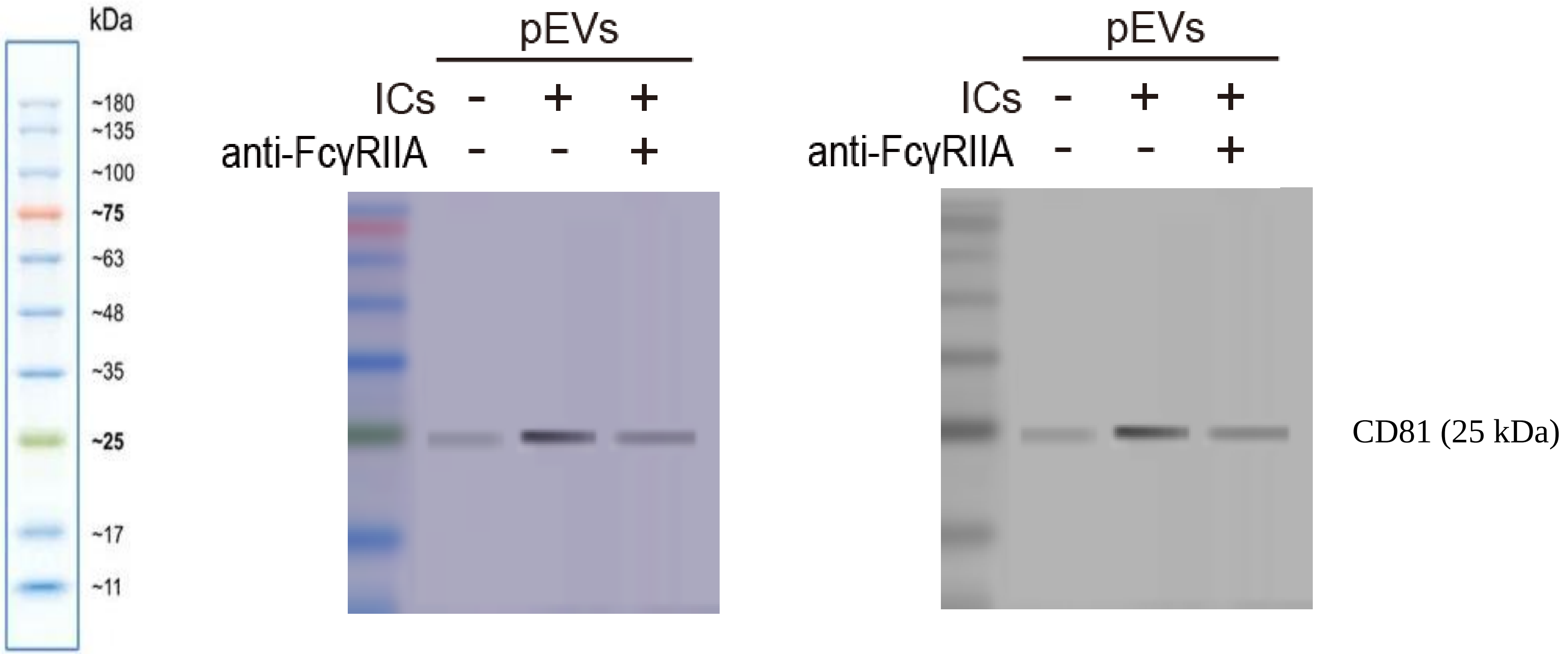
kDa
~180
~135
~100
~75
~63
~48
~35
~25
~17
~11
pEVs
ICs - + +
anti-FcγRIIA - - +
pEVs
ICs - + +
anti-FcγRIIA - - +
CD81 (25 kDa)

Fig. 2E

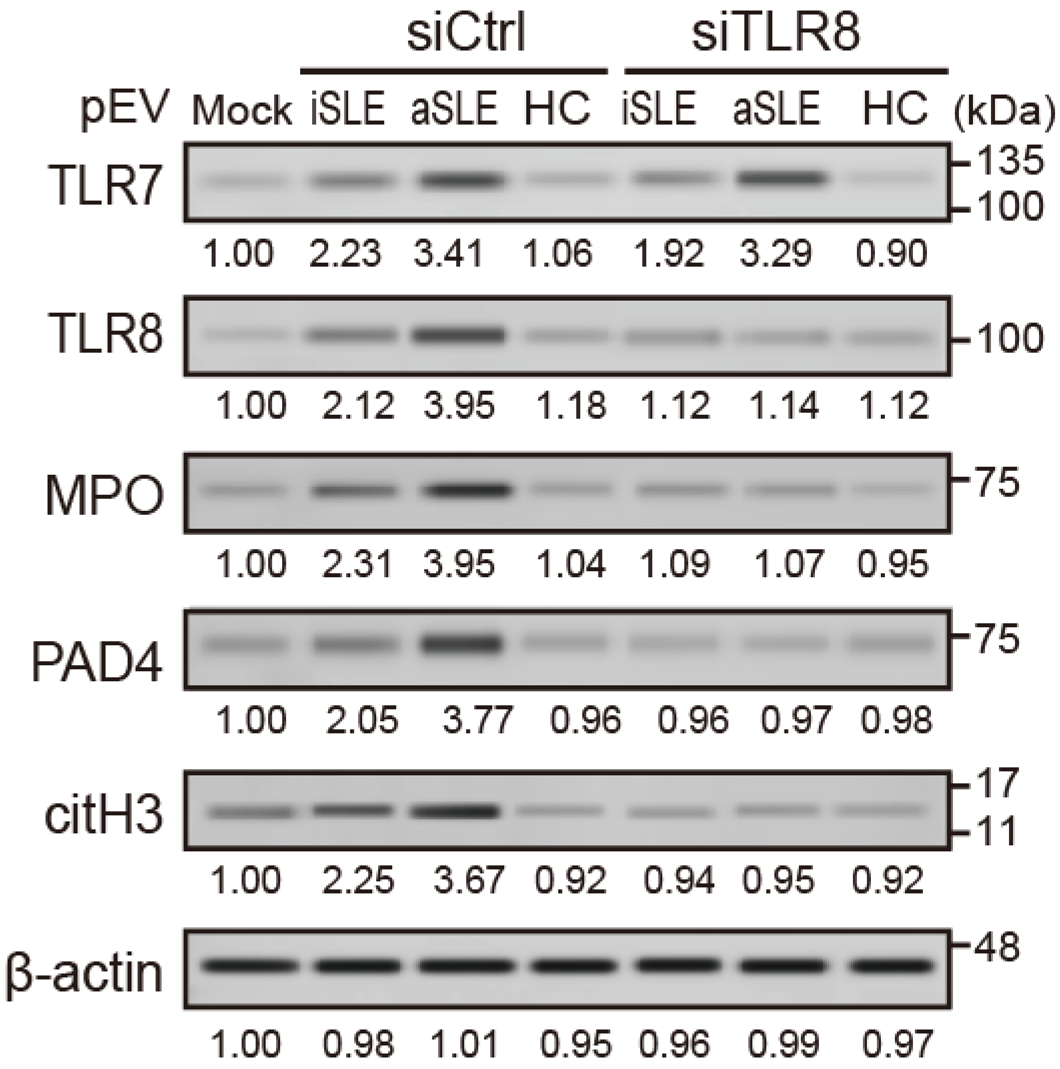
siCtrl
siTLR8
pEV
Mock
iSLE
aSLE
HC
iSLE
aSLE
HC
(kDa)
TLR7
135
100
1.00 2.23 3.41 1.06 1.92 3.29 0.90
TLR8
100
1.00 2.12 3.95 1.18 1.12 1.14 1.12
MPO
75
1.00 2.31 3.95 1.04 1.09 1.07 0.95
PAD4
75
1.00 2.05 3.77 0.96 0.96 0.97 0.98
citH3
17
11
1.00 2.25 3.67 0.92 0.94 0.95 0.92
β-actin
48
1.00 0.98 1.01 0.95 0.96 0.99 0.97

TLR7 (121 kDa)

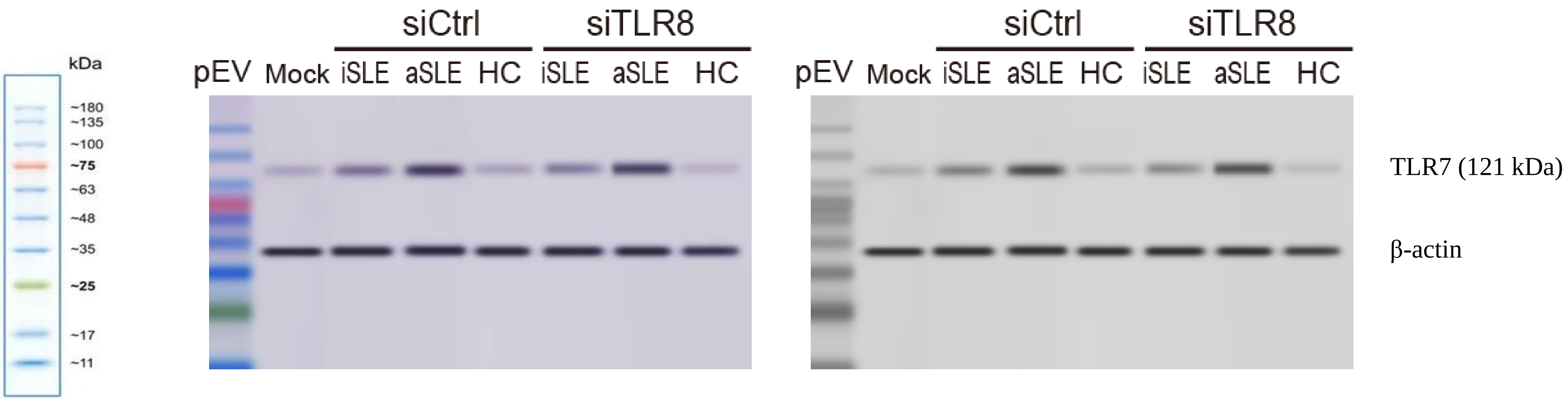
kDa
~180
~135
~100
~75
~63
~48
~35
~25
~17
~11
siCtrl
siTLR8
pEV
Mock
iSLE
aSLE
HC
iSLE
aSLE
HC
siCtrl
siTLR8
pEV
Mock
iSLE
aSLE
HC
iSLE
aSLE
HC
TLR7 (121 kDa)
β-actin

TLR8 (110 kDa)

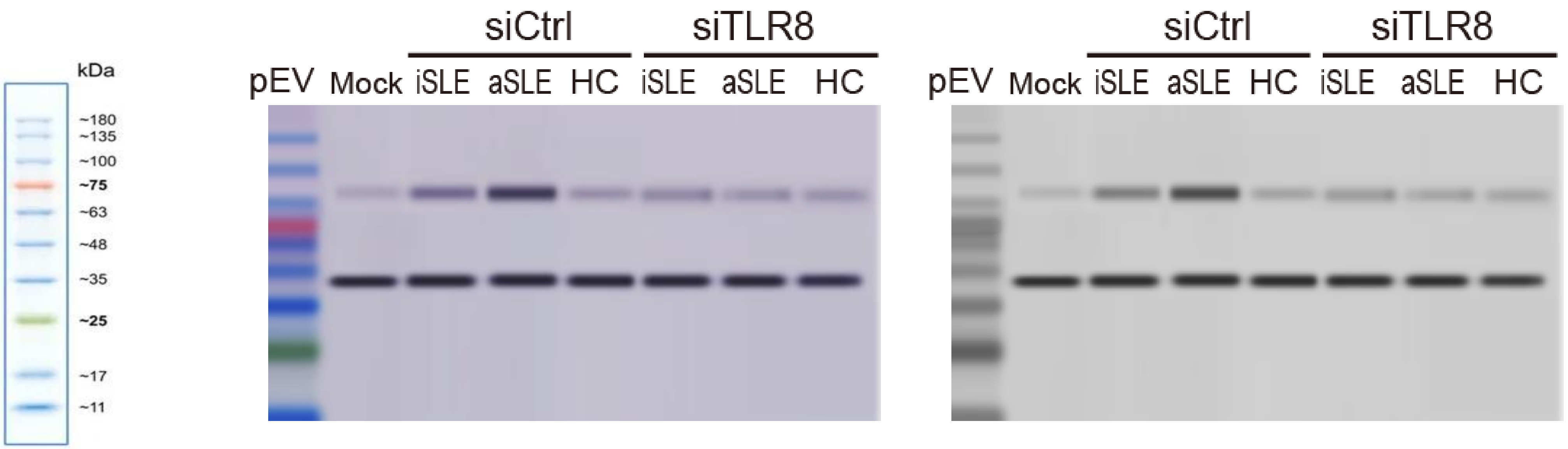

TLR8 (110 kDa)

β-actin

MPO (72 kDa)

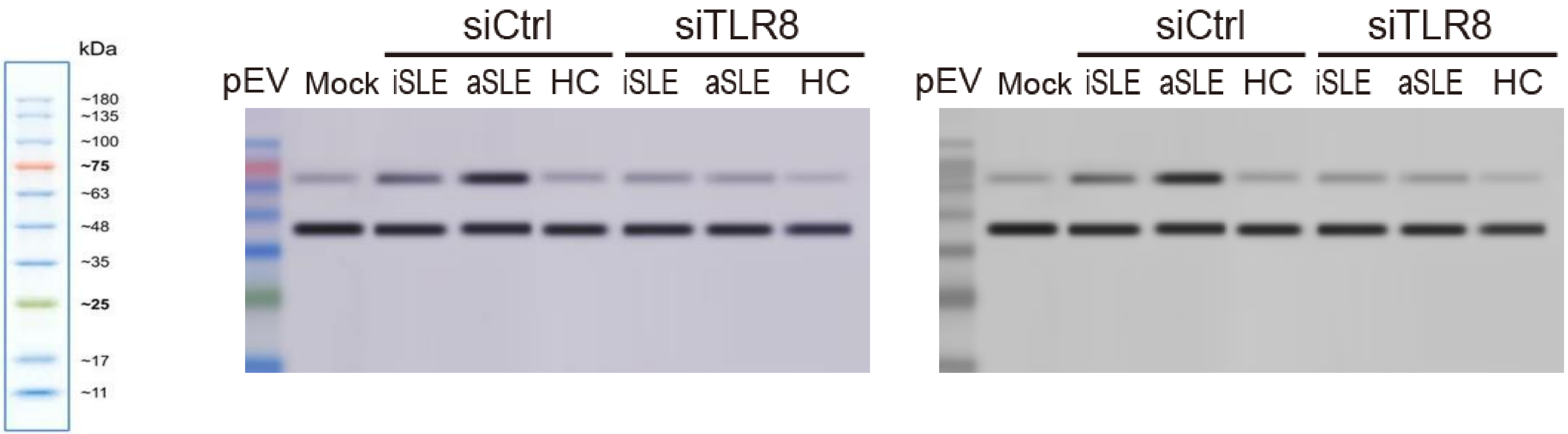

MPO

β-actin

PAD4(72 kDa)

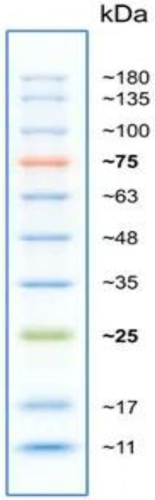

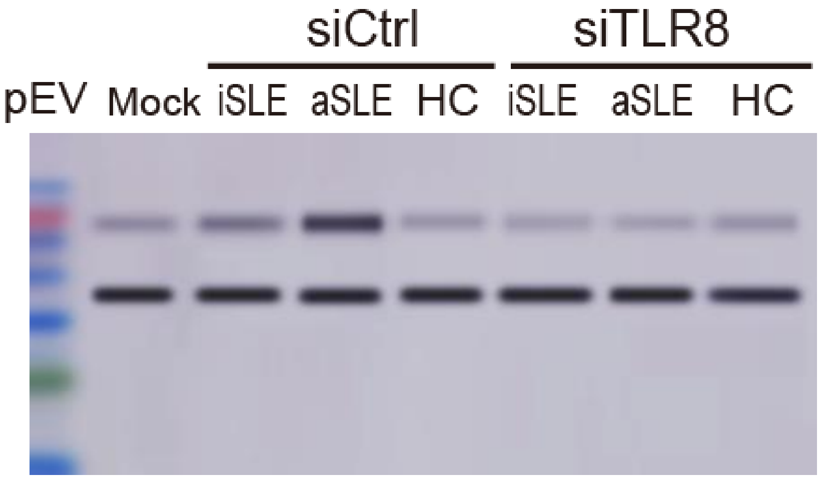

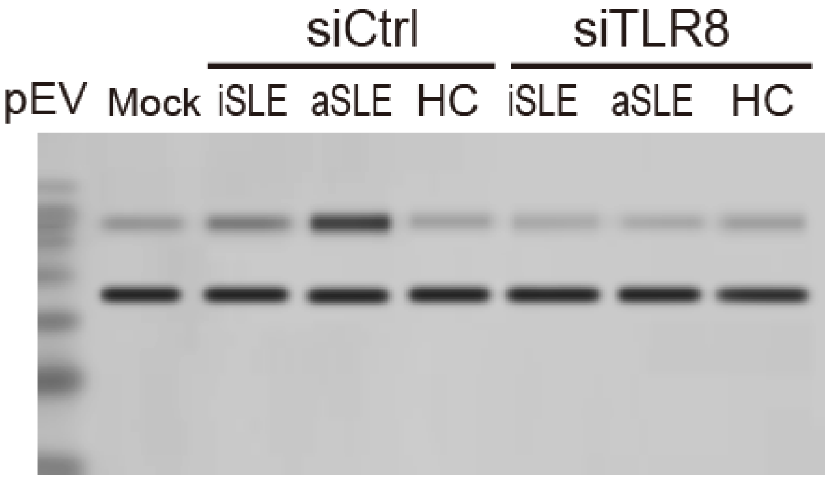

PAD4(72 kDa)

β–actin

citH3(14~17 kDa)

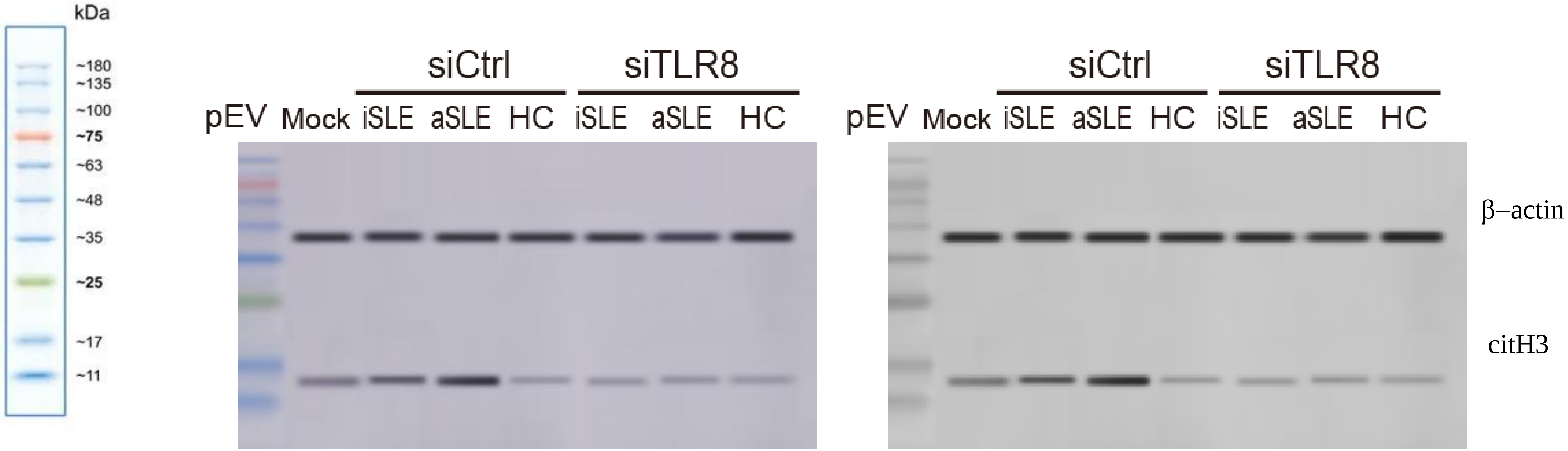

Fig. 2H

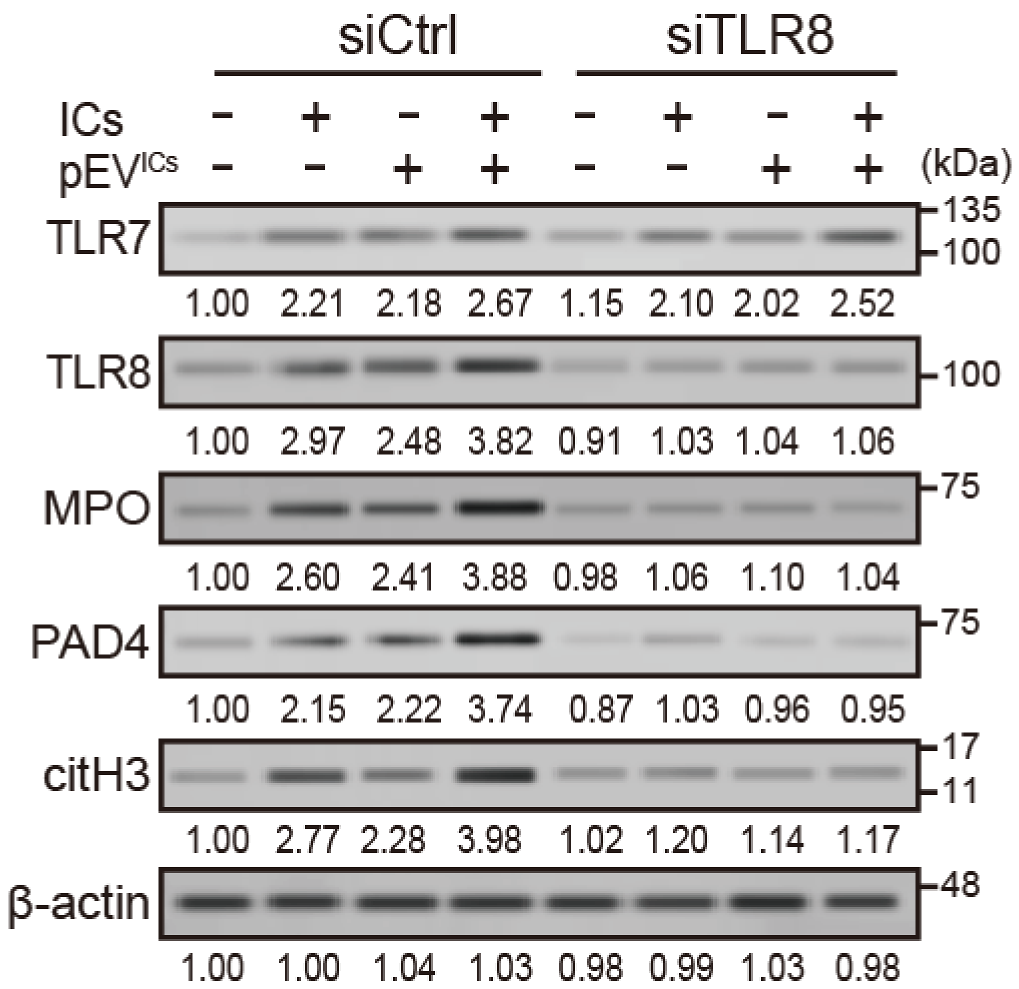

siCtrl
siTLR8
ICs - + - + - + - +
pEV[ICs] - - + + - - + +
(kDa)
TLR7
135
100
1.00 2.21 2.18 2.67 1.15 2.10 2.02 2.52
TLR8
100
1.00 2.97 2.48 3.82 0.91 1.03 1.04 1.06
MPO
75
1.00 2.60 2.41 3.88 0.98 1.06 1.10 1.04
PAD4
75
1.00 2.15 2.22 3.74 0.87 1.03 0.96 0.95
citH3
17
11
1.00 2.77 2.28 3.98 1.02 1.20 1.14 1.17
β-actin
48
1.00 1.00 1.04 1.03 0.98 0.99 1.03 0.98

TLR7 (121 kDa)

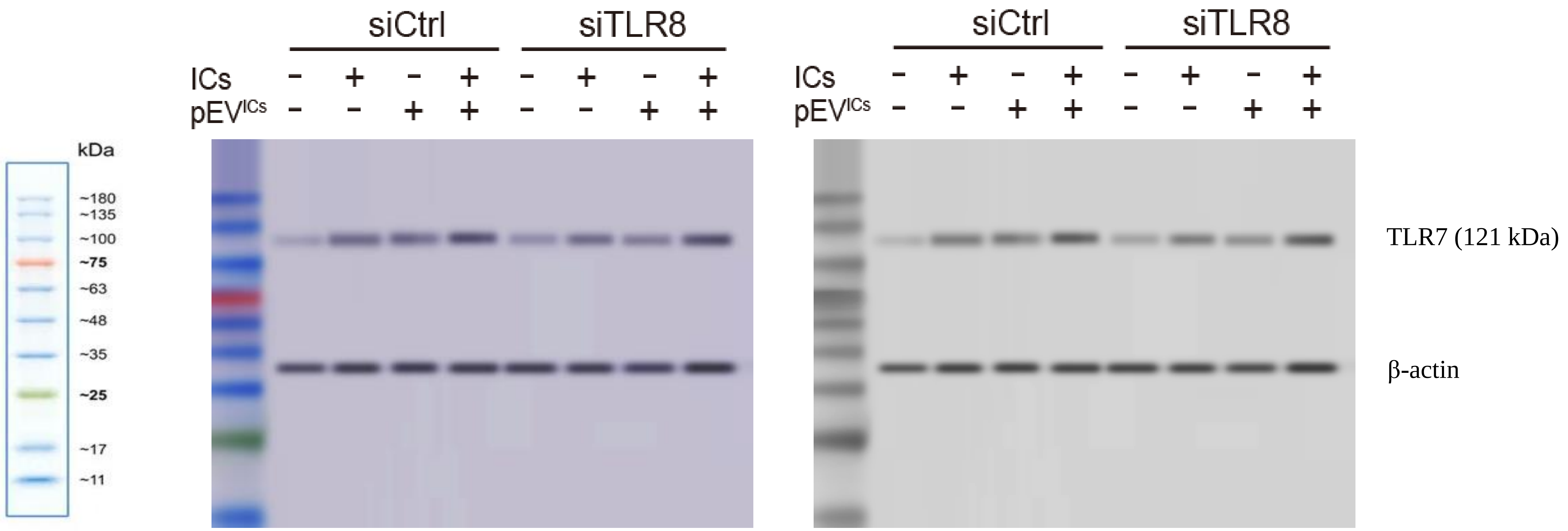
kDa
~180
~135
~100
~75
~63
~48
~35
~25
~17
~11
siCtrl
siTLR8
ICs - + - + - + - +
pEVICs - - + + - - + +
siCtrl
siTLR8
ICs - + - + - + - +
pEVICs - - + + - - + +
TLR7 (121 kDa)
β-actin

TLR8 (110 kDa)

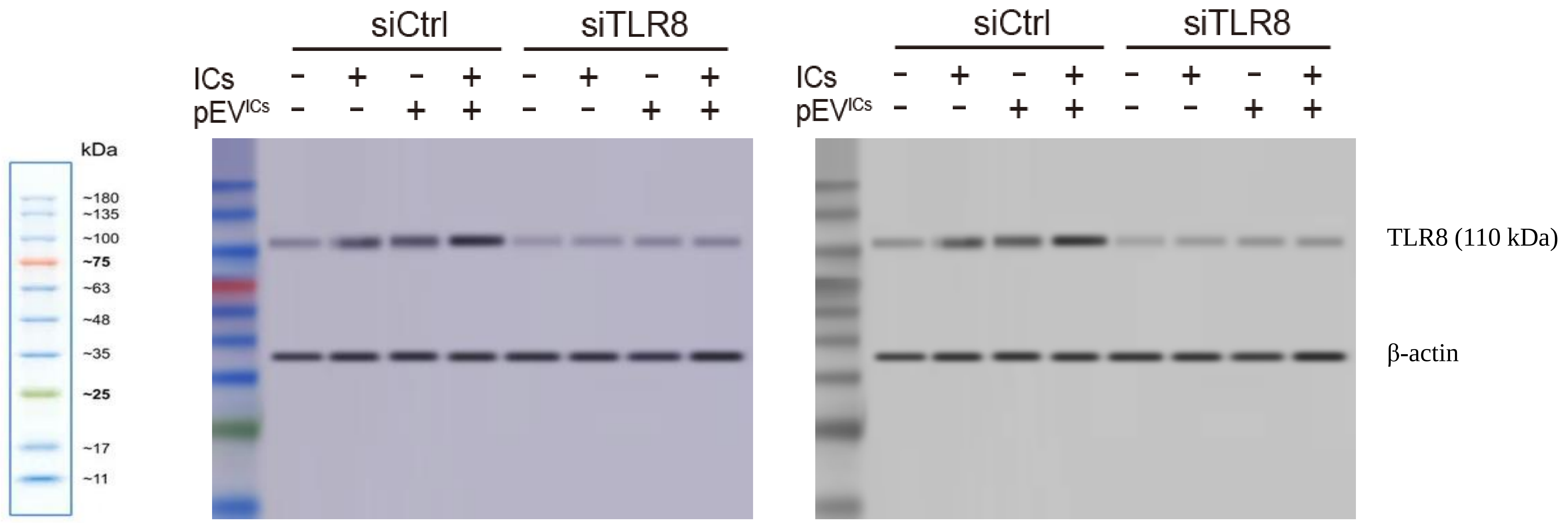
kDa
~180
~135
~100
~75
~63
~48
~35
~25
~17
~11
siCtrl
siTLR8
ICs – + – + – + – +
pEV^ICs – – + + – – + +
siCtrl
siTLR8
ICs – + – + – + – +
pEV^ICs – – + + – – + +
TLR8 (110 kDa)
β-actin

MPO (72 kDa)

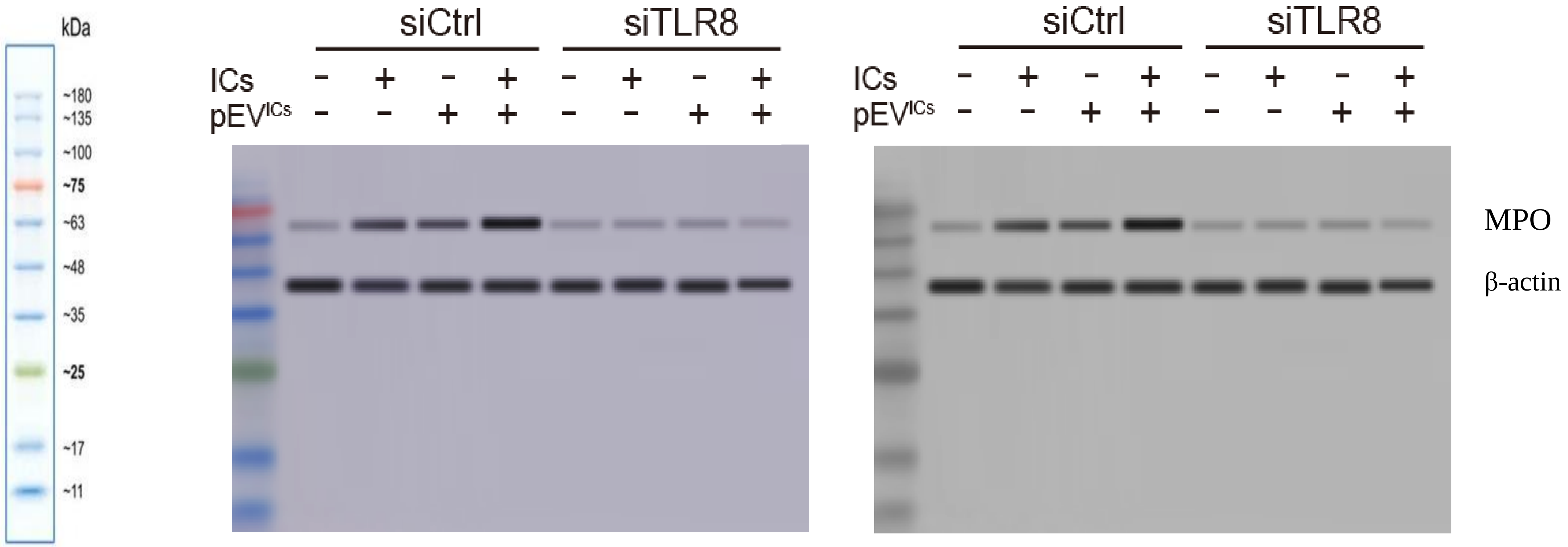
kDa
~180
~135
~100
~75
~63
~48
~35
~25
~17
~11
siCtrl
siTLR8
ICs - + - + - + - +
pEV^ICs - - + + - - + +
siCtrl
siTLR8
ICs - + - + - + - +
pEV^ICs - - + + - - + +
MPO
β-actin

PAD4(72 kDa)

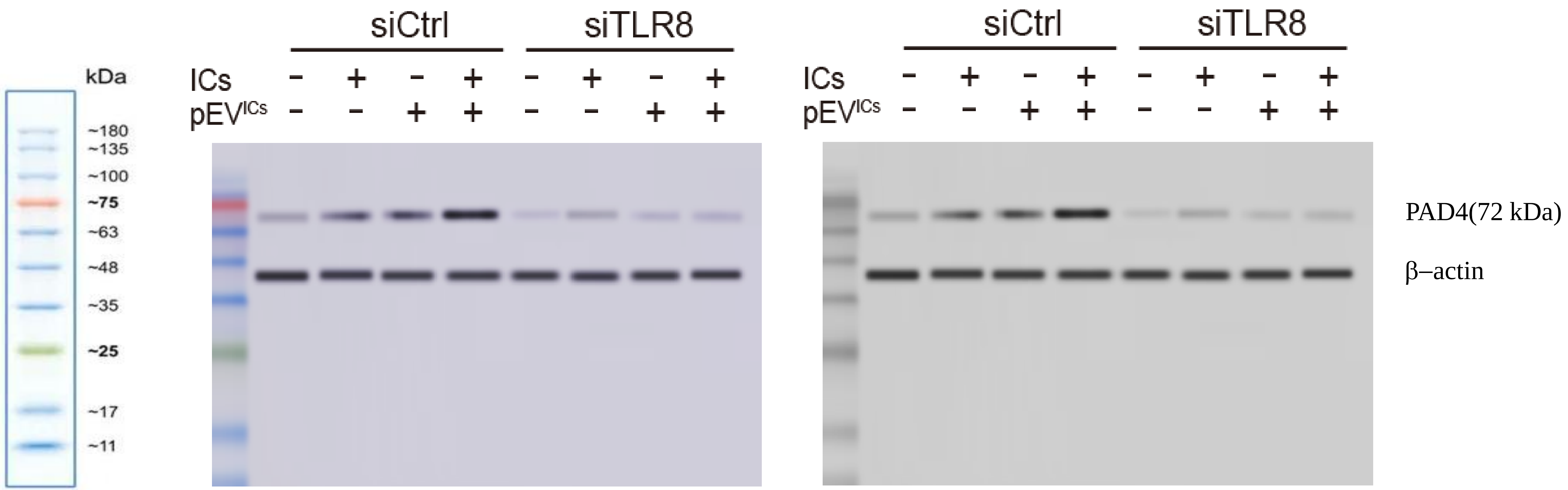
kDa
~180
~135
~100
~75
~63
~48
~35
~25
~17
~11
siCtrl
siTLR8
ICs - + - + - + - +
pEVICs - - + + - - + +
siCtrl
siTLR8
ICs - + - + - + - +
pEVICs - - + + - - + +
PAD4(72 kDa)
β–actin

citH3(14~17 kDa)

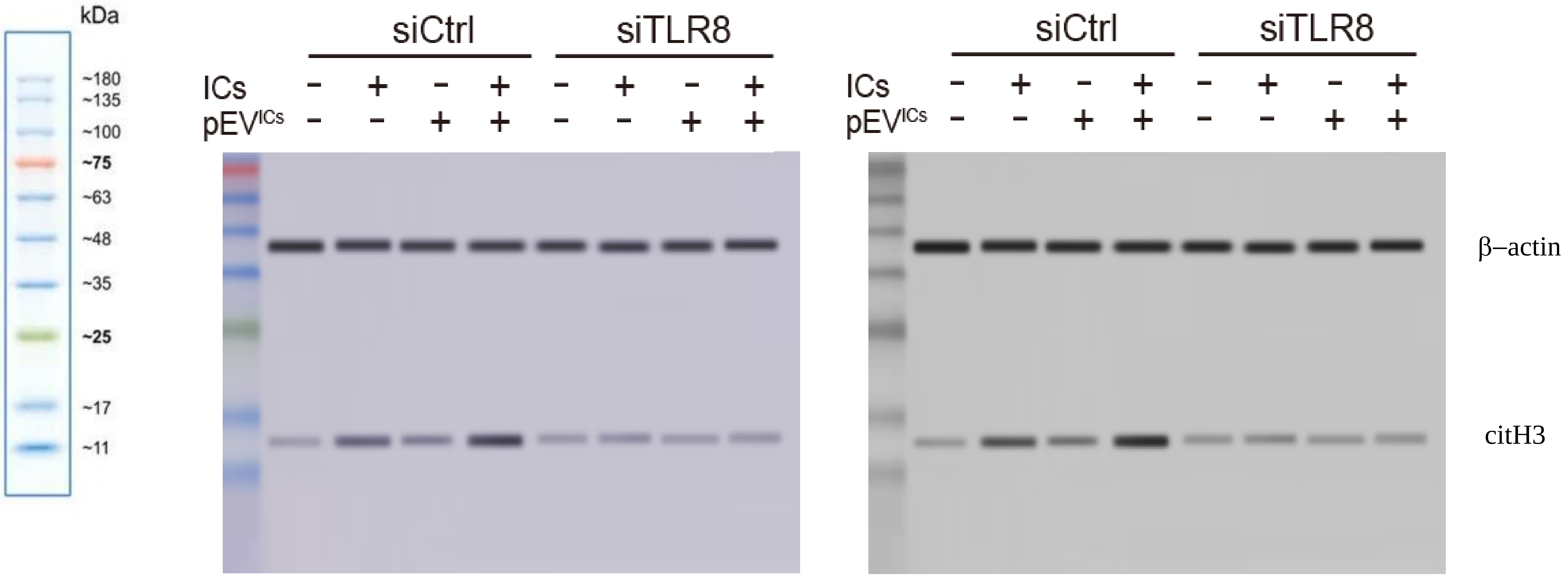

Fig. 3H

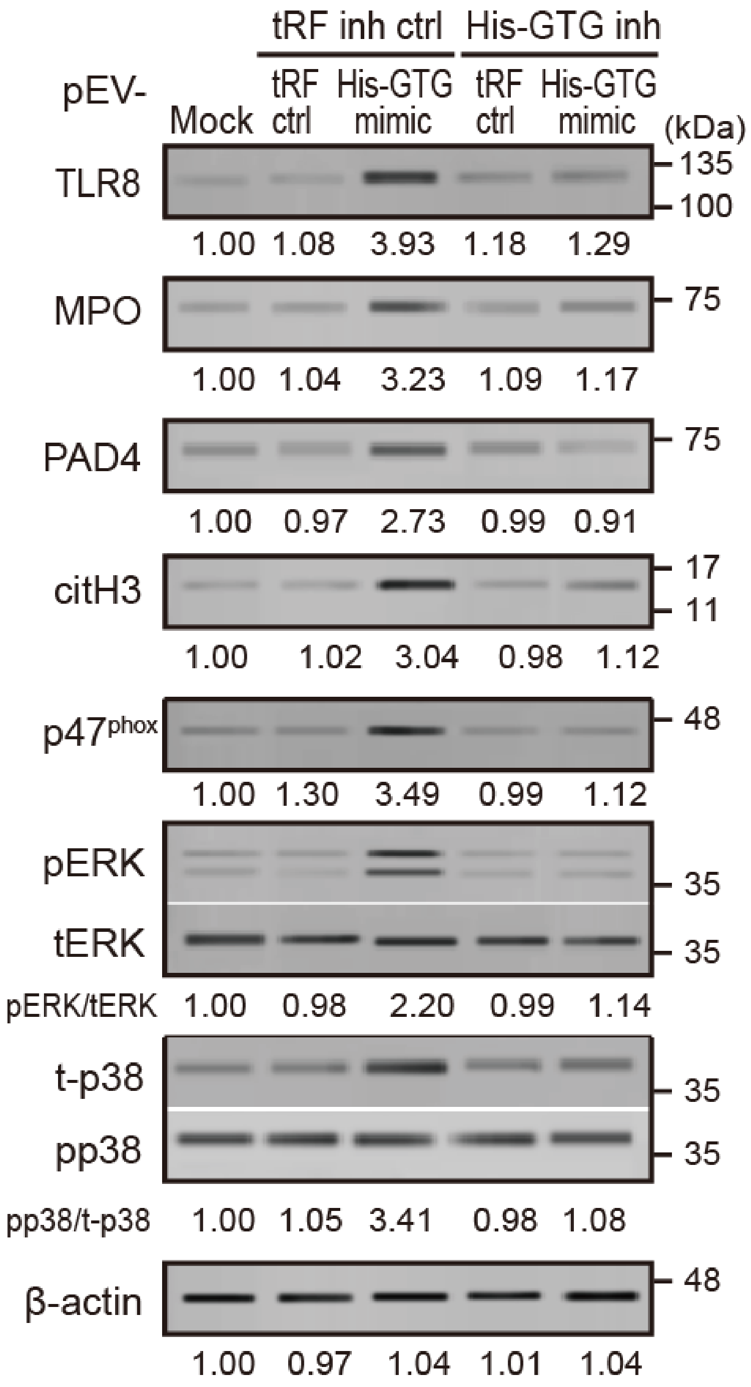

tRF inh ctrl
His-GTG inh
pEV-
Mock
tRF ctrl
His-GTG mimic
tRF ctrl
His-GTG mimic
(kDa)
TLR8
135
100
1.00 1.08 3.93 1.18 1.29
MPO
75
1.00 1.04 3.23 1.09 1.17
PAD4
75
1.00 0.97 2.73 0.99 0.91
citH3
17
11
1.00 1.02 3.04 0.98 1.12
p47phox
48
1.00 1.30 3.49 0.99 1.12
pERK
35
tERK
35
pERK/tERK 1.00 0.98 2.20 0.99 1.14
t-p38
35
pp38
35
pp38/t-p38 1.00 1.05 3.41 0.98 1.08
β-actin
48
1.00 0.97 1.04 1.01 1.04

TLR8 (110 kDa)

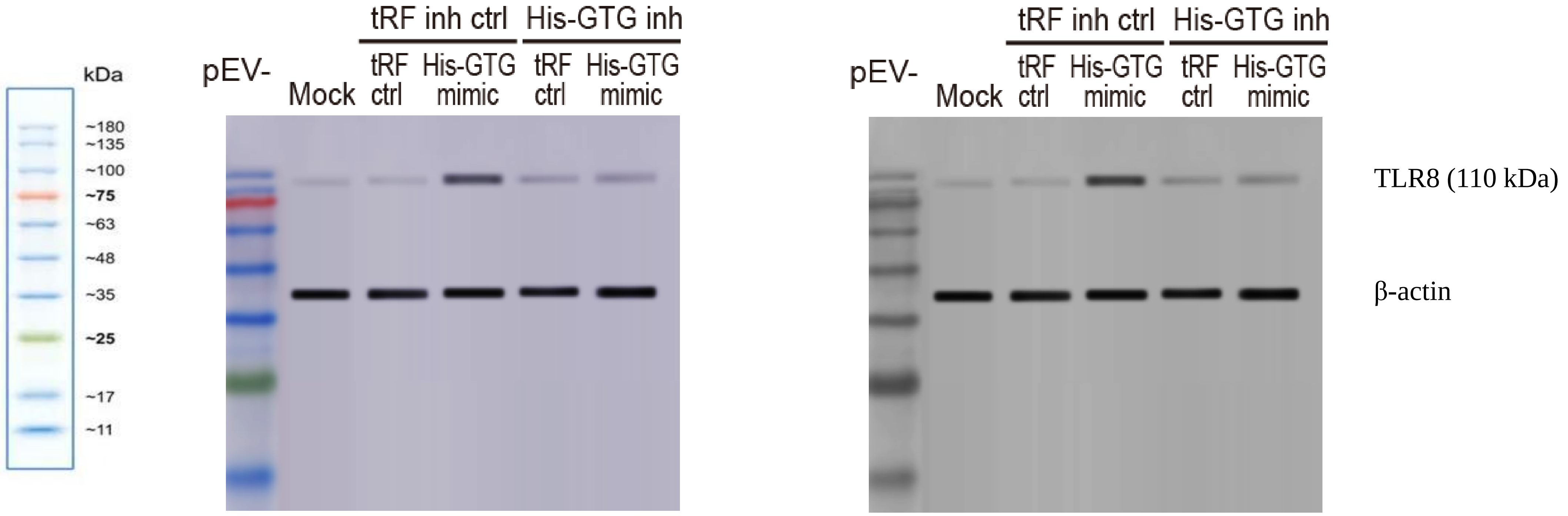
kDa
~180
~135
~100
~75
~63
~48
~35
~25
~17
~11
pEV-
Mock
tRF inh ctrl
His-GTG inh
tRF ctrl
His-GTG mimic
tRF ctrl
His-GTG mimic
pEV-
Mock
tRF inh ctrl
His-GTG inh
tRF ctrl
His-GTG mimic
tRF ctrl
His-GTG mimic
TLR8 (110 kDa)
β-actin

MPO (72 kDa)

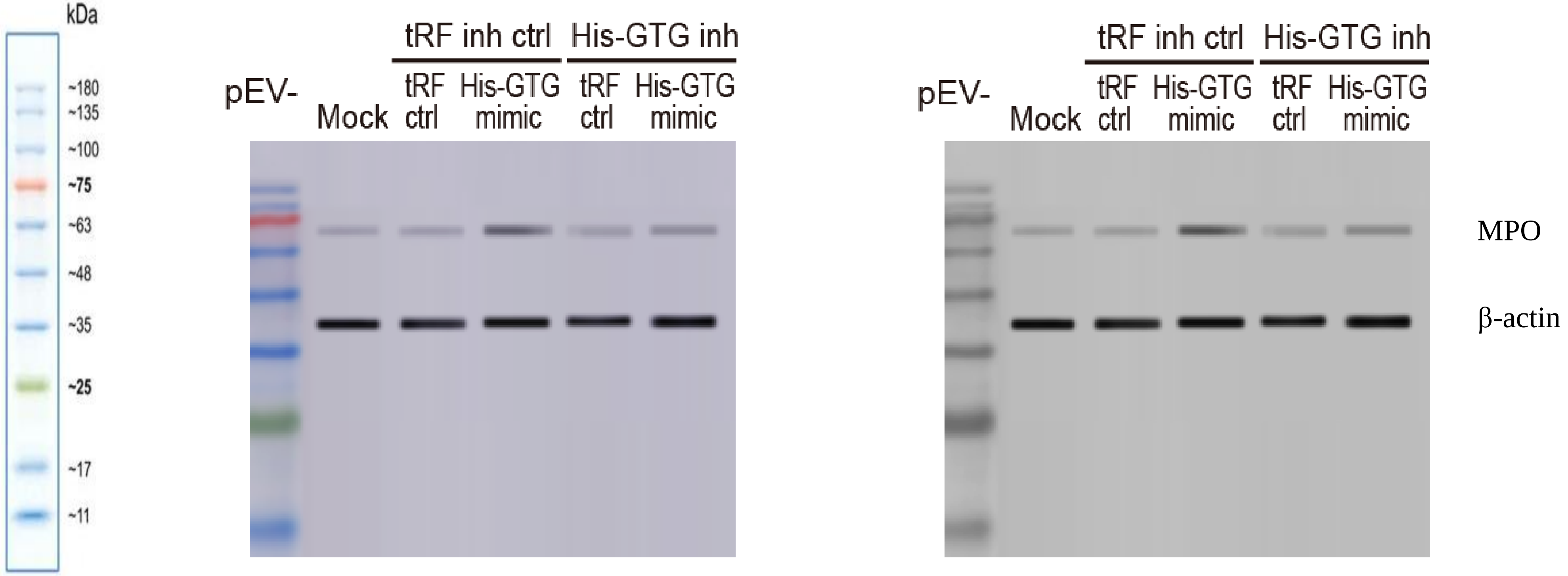

kDa
~180
~135
~100
~75
~63
~48
~35
~25
~17
~11
tRF inh ctrl
His-GTG inh
pEV-
Mock
tRF ctrl
His-GTG mimic
tRF ctrl
His-GTG mimic
tRF inh ctrl
His-GTG inh
pEV-
Mock
tRF ctrl
His-GTG mimic
tRF ctrl
His-GTG mimic
MPO
β-actin

PAD4(72 kDa)

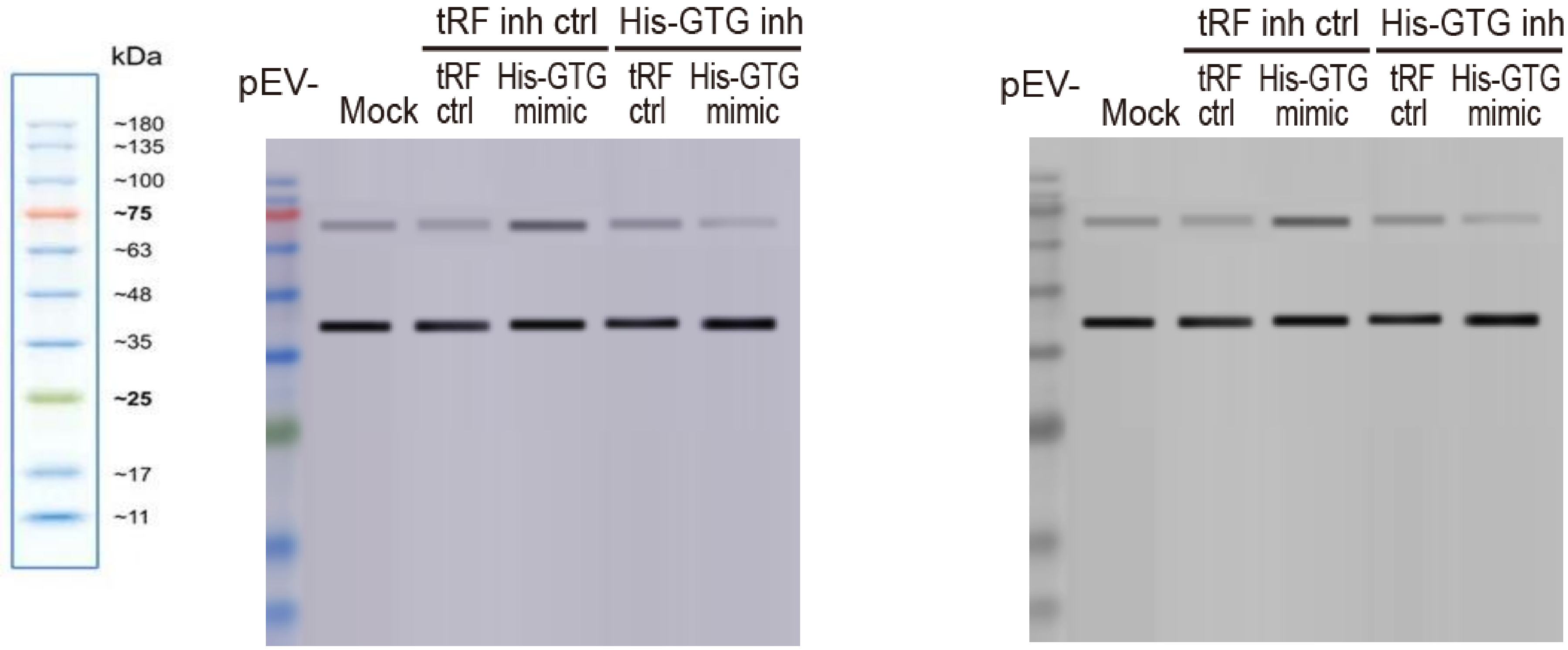

PAD4(72 kDa)

β–actin

citH3(14~17 kDa)

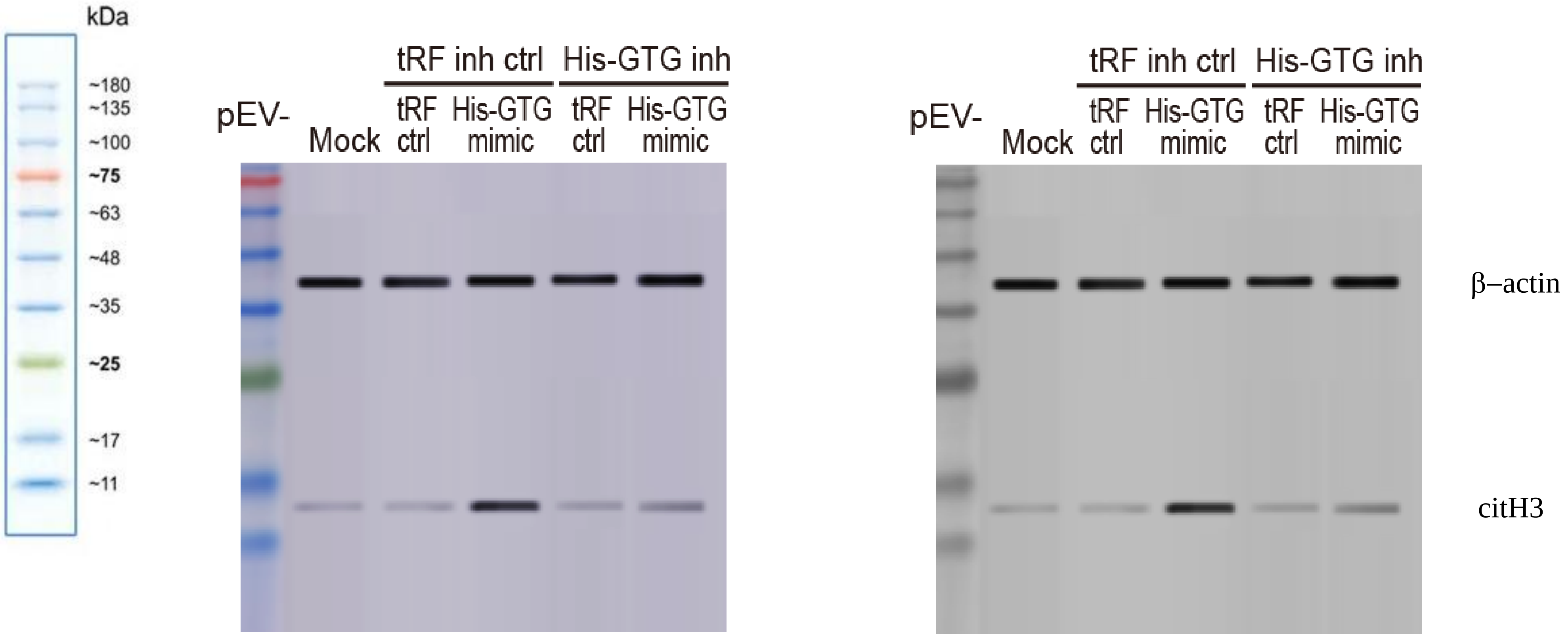

p47 phox (47 kDa)

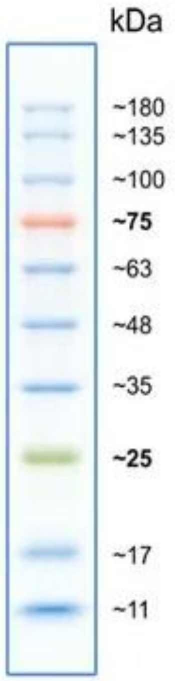

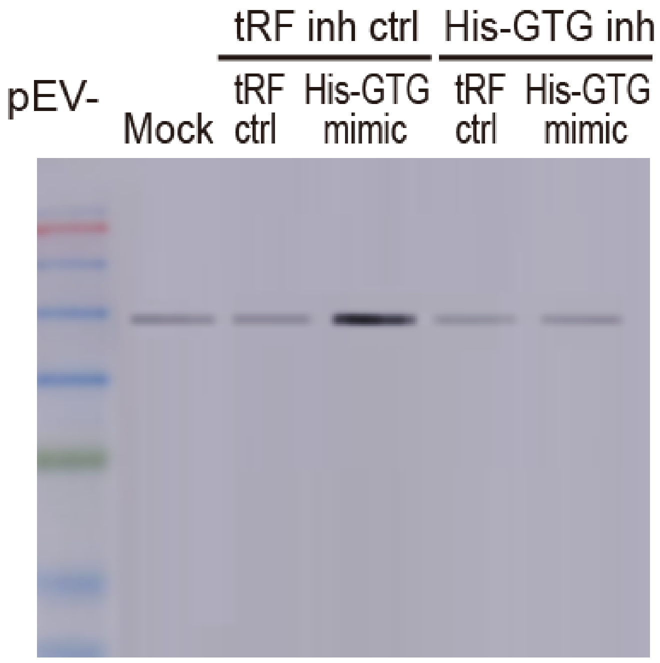

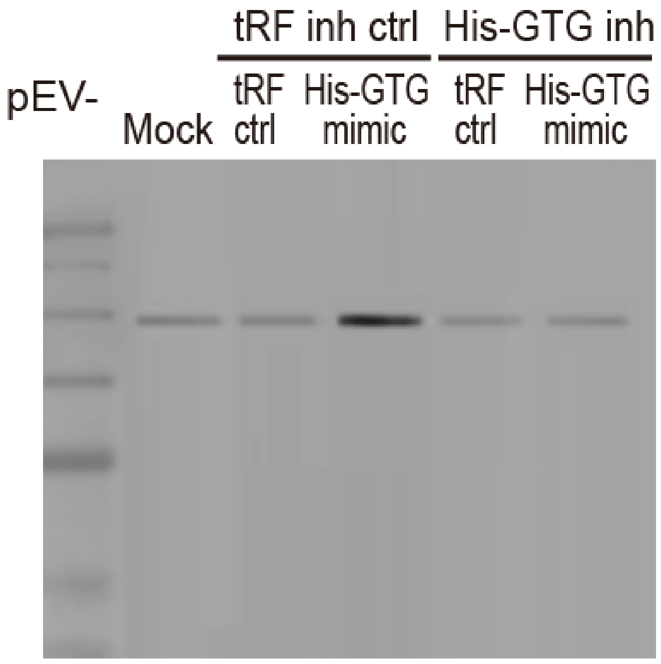

p47 phox (47 kDa)

pERK (42, 44 kDa)

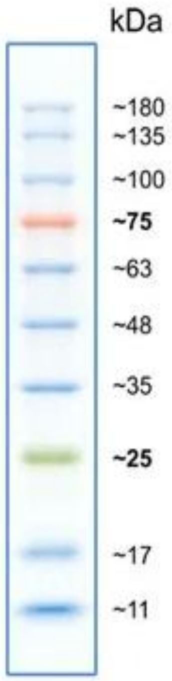

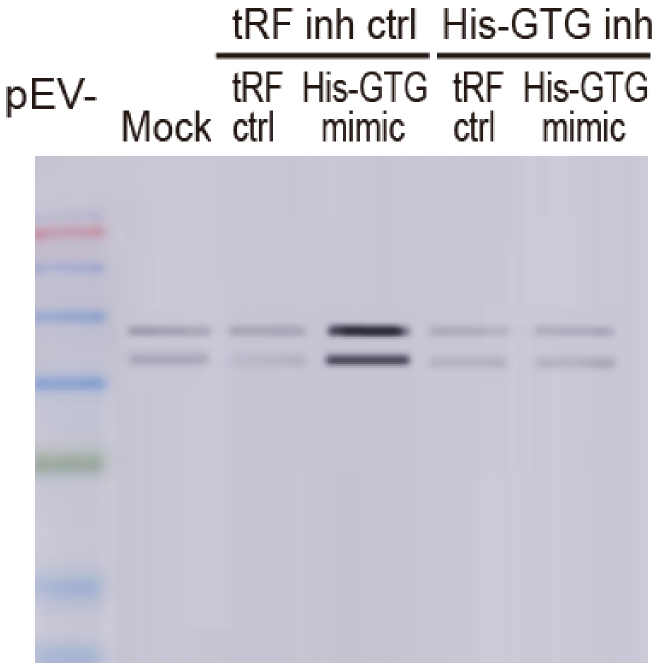

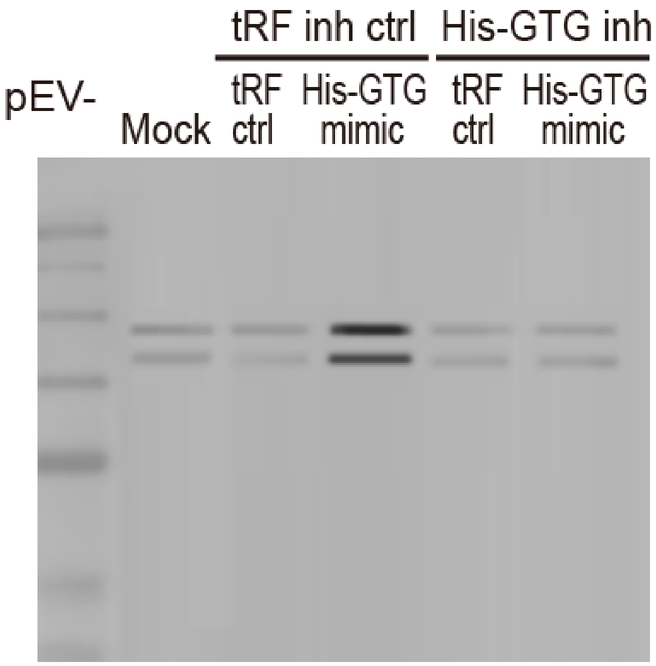

pERK (42,44 kDa)

tERK (44 kDa)

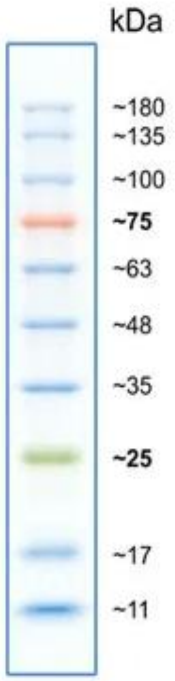

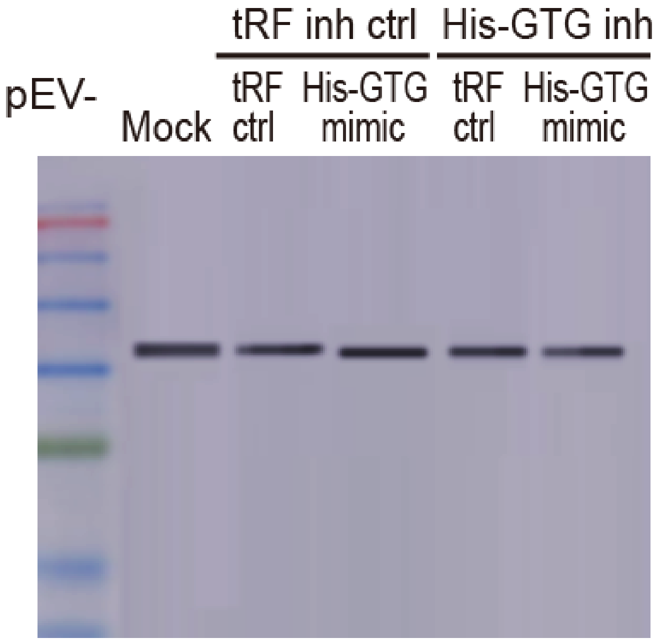

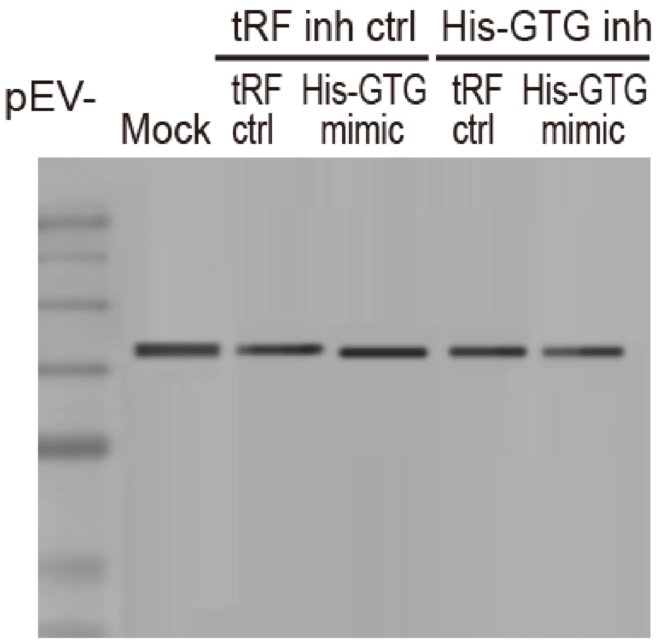

tERK (44 kDa)

Phos-p38 (43 kDa)

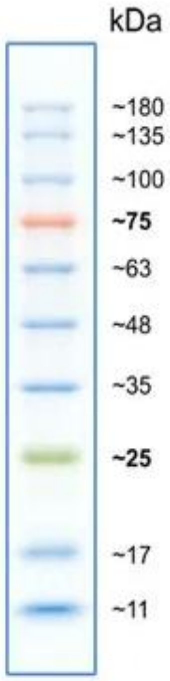

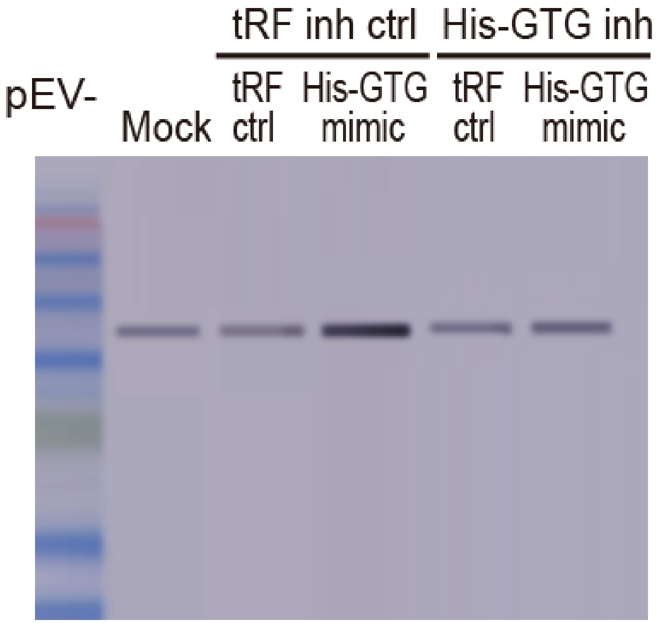

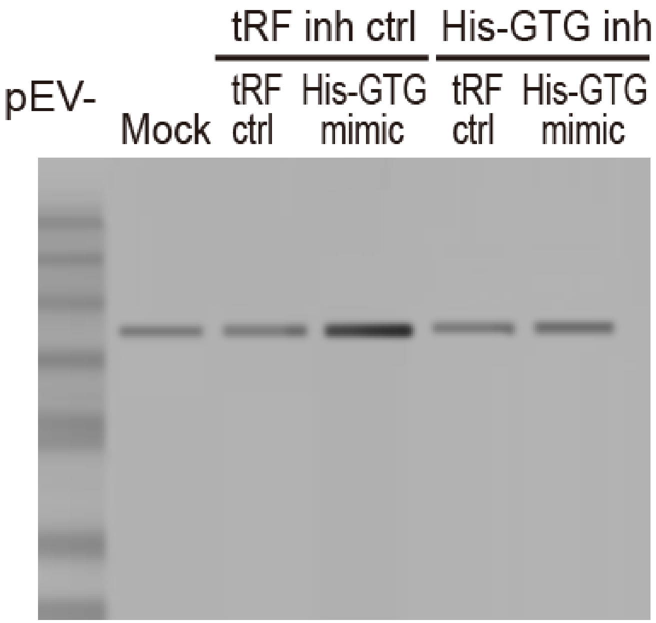

Phos-p38 (43 kDa)

t-p38 (40 kDa)

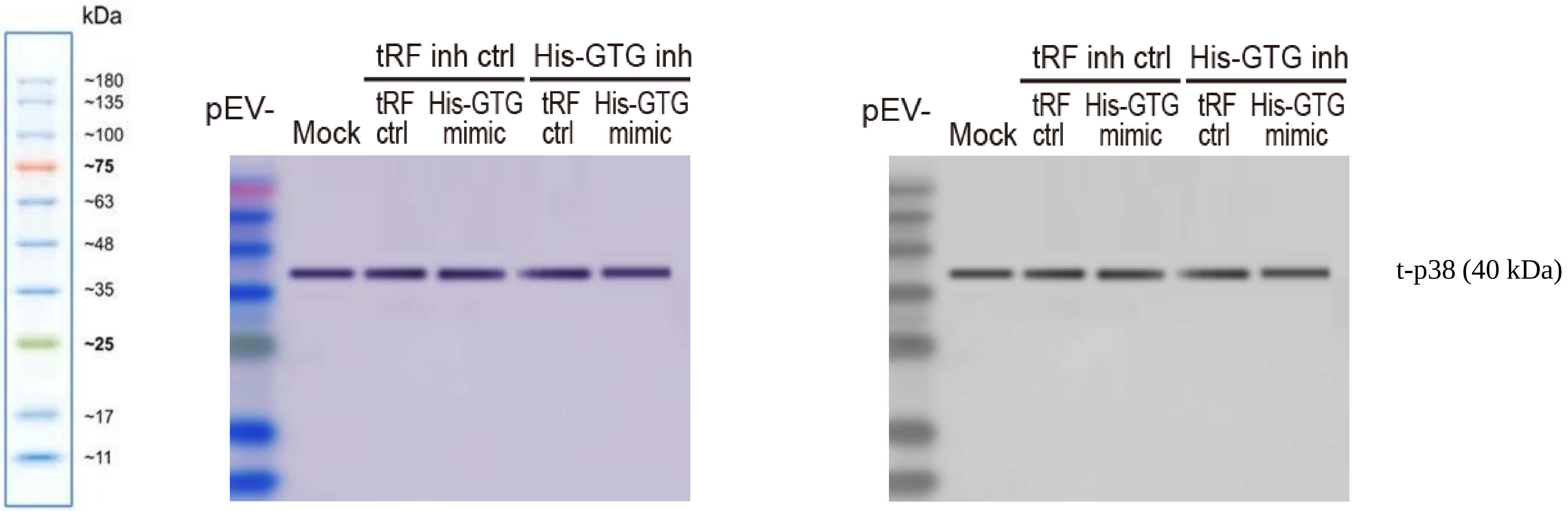
kDa
~180
~135
~100
~75
~63
~48
~35
~25
~17
~11
pEV-
Mock
tRF inh ctrl
His-GTG inh
tRF ctrl
His-GTG mimic
tRF ctrl
His-GTG mimic
pEV-
Mock
tRF inh ctrl
His-GTG inh
tRF ctrl
His-GTG mimic
tRF ctrl
His-GTG mimic
t-p38 (40 kDa)

Fig. 4D

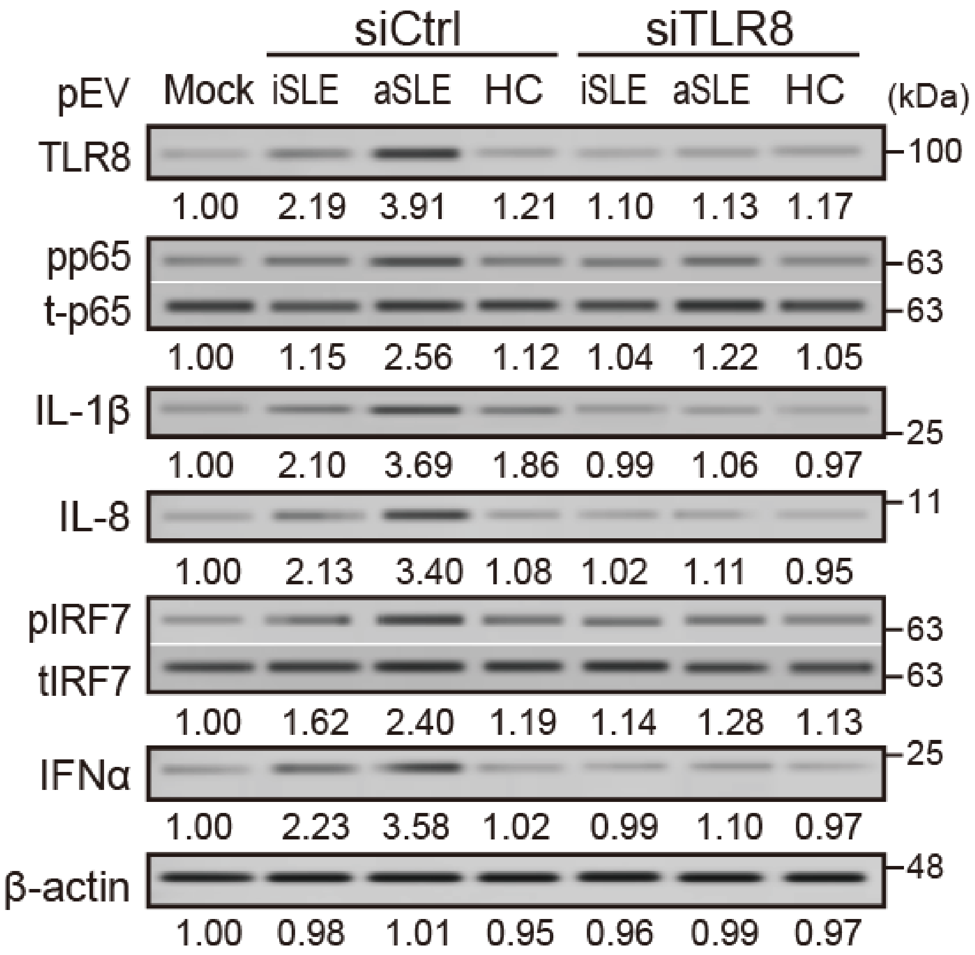
siCtrl
siTLR8
pEV Mock iSLE aSLE HC iSLE aSLE HC
(kDa)
TLR8
100
1.00 2.19 3.91 1.21 1.10 1.13 1.17
pp65
t-p65
63
63
1.00 1.15 2.56 1.12 1.04 1.22 1.05
IL-1β
25
1.00 2.10 3.69 1.86 0.99 1.06 0.97
IL-8
11
1.00 2.13 3.40 1.08 1.02 1.11 0.95
pIRF7
tIRF7
63
63
1.00 1.62 2.40 1.19 1.14 1.28 1.13
IFNα
25
1.00 2.23 3.58 1.02 0.99 1.10 0.97
β-actin
48
1.00 0.98 1.01 0.95 0.96 0.99 0.97

TLR8 (110 kDa)

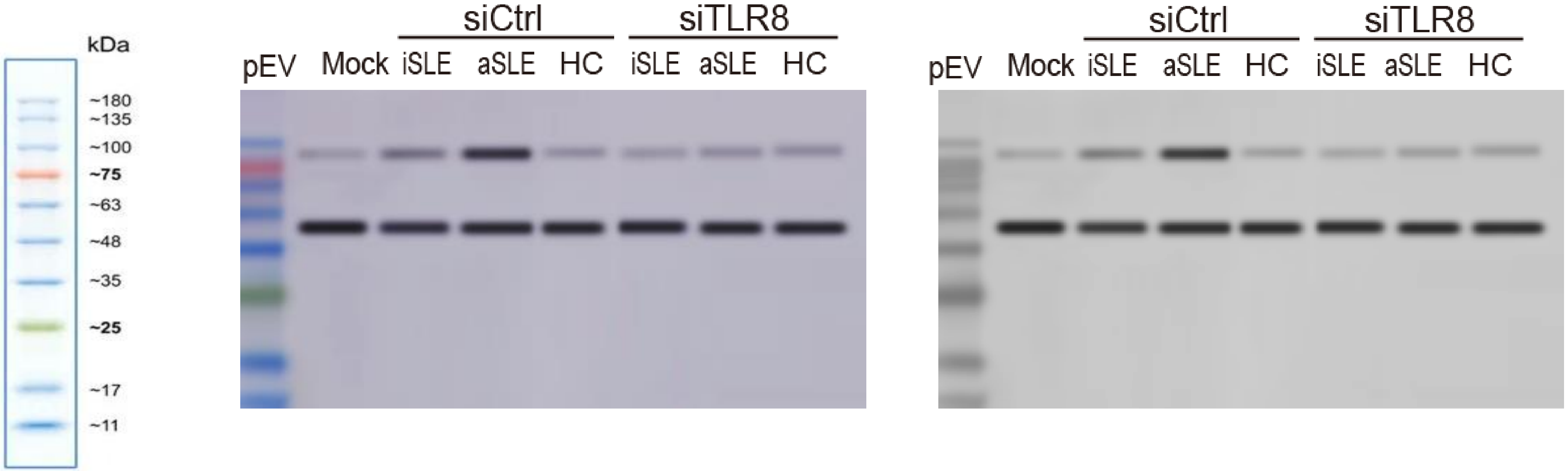

TLR8 (110 kDa)

β-actin

Phos-p65 (65 kDa)

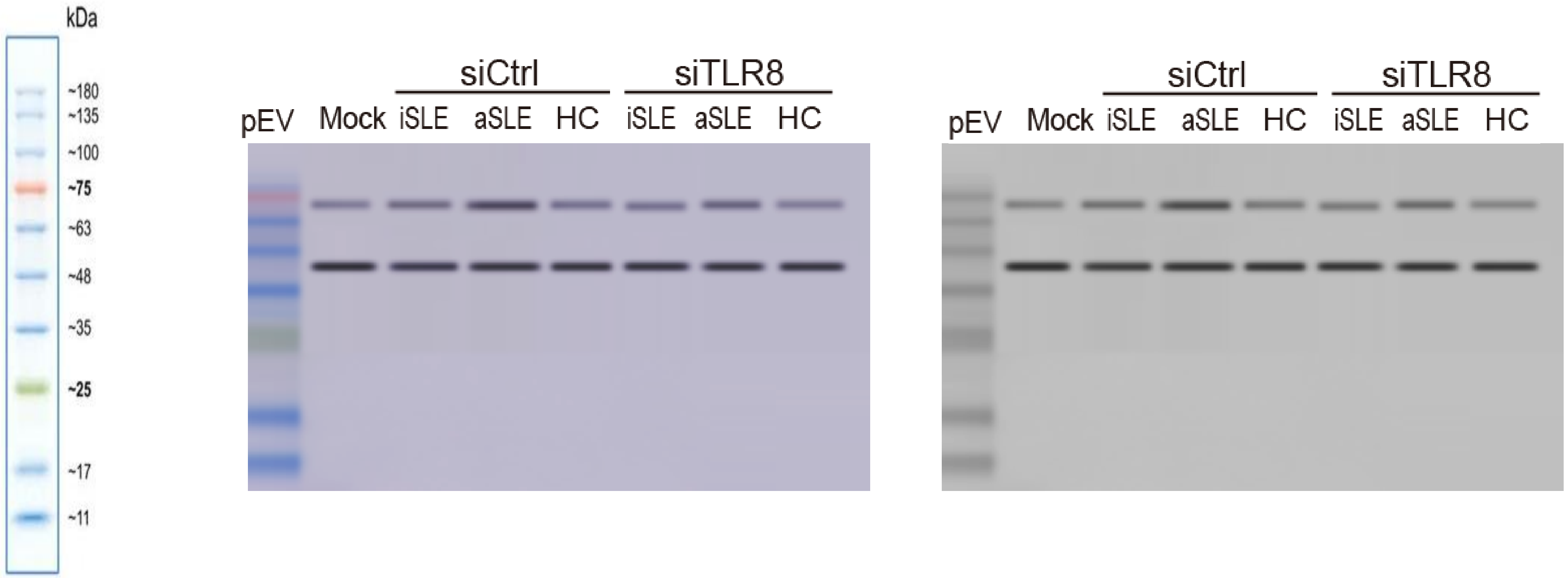

Phos-p65 (65 kDa)

β-actin

t-p65 (65 kDa)

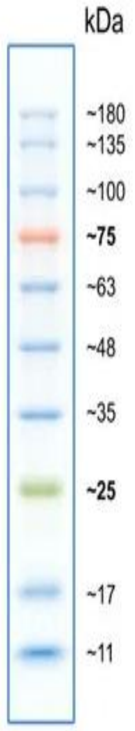

kDa
~180
~135
~100
~75
~63
~48
~35
~25
~17
~11

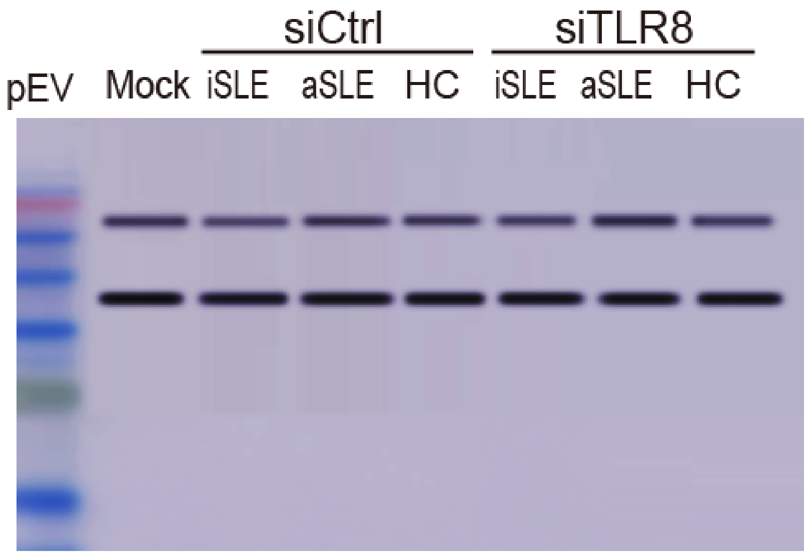

siCtrl
siTLR8
pEV
Mock
iSLE
aSLE
HC
iSLE
aSLE
HC

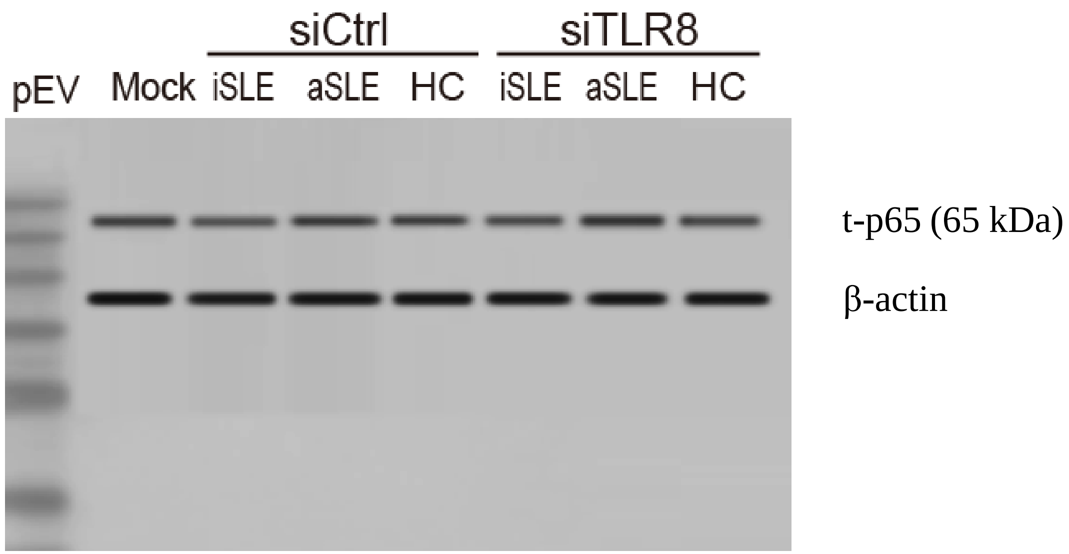

siCtrl
siTLR8
pEV
Mock
iSLE
aSLE
HC
iSLE
aSLE
HC
t-p65 (65 kDa)
β-actin

IL-1β (31 kDa)

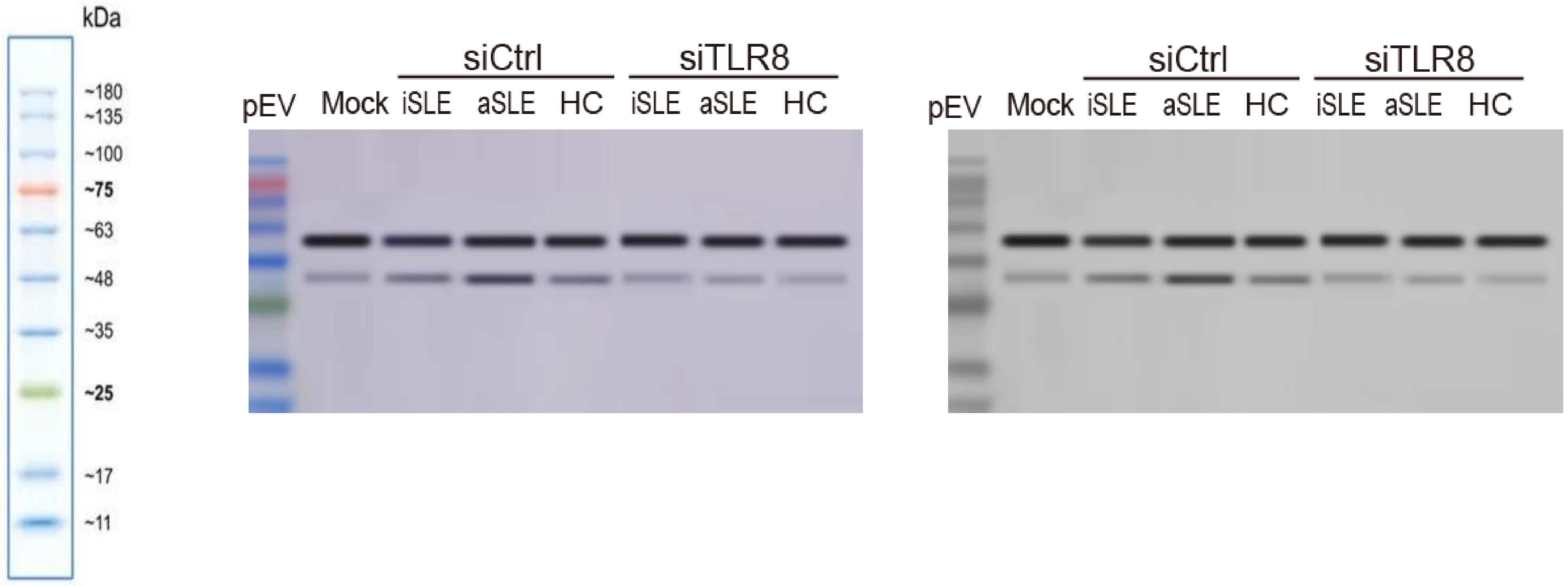

β-actin

IL-1β (31 kDa)

IL-8 (8 kDa)

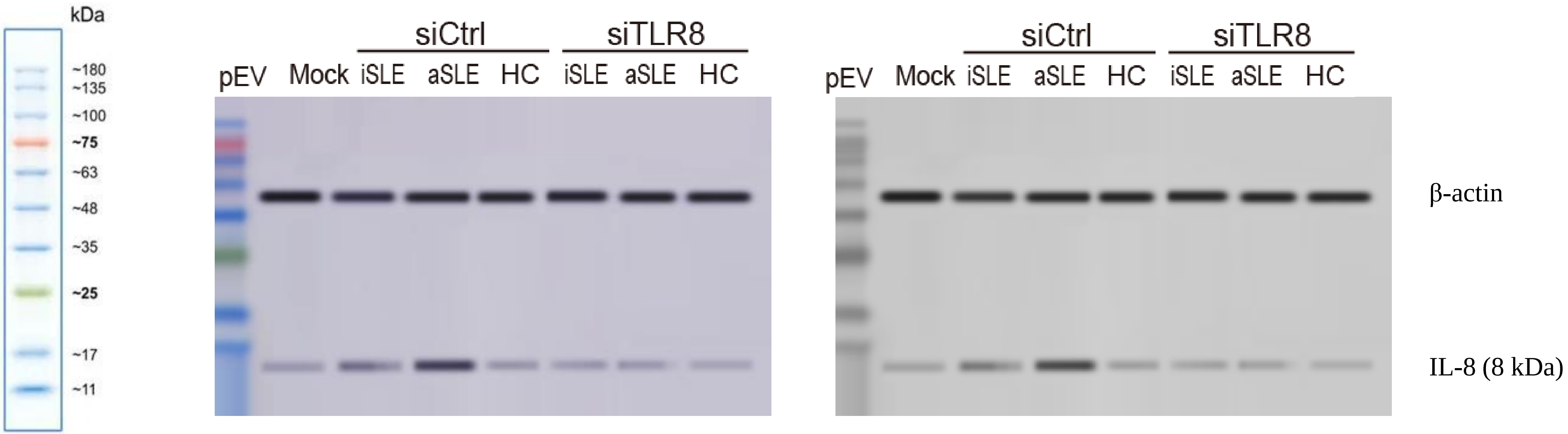
kDa
~180
~135
~100
~75
~63
~48
~35
~25
~17
~11
siCtrl
siTLR8
pEV
Mock
iSLE
aSLE
HC
iSLE
aSLE
HC
siCtrl
siTLR8
pEV
Mock
iSLE
aSLE
HC
iSLE
aSLE
HC
β-actin
IL-8 (8 kDa)

Phos-IRF7 (65 kDa)

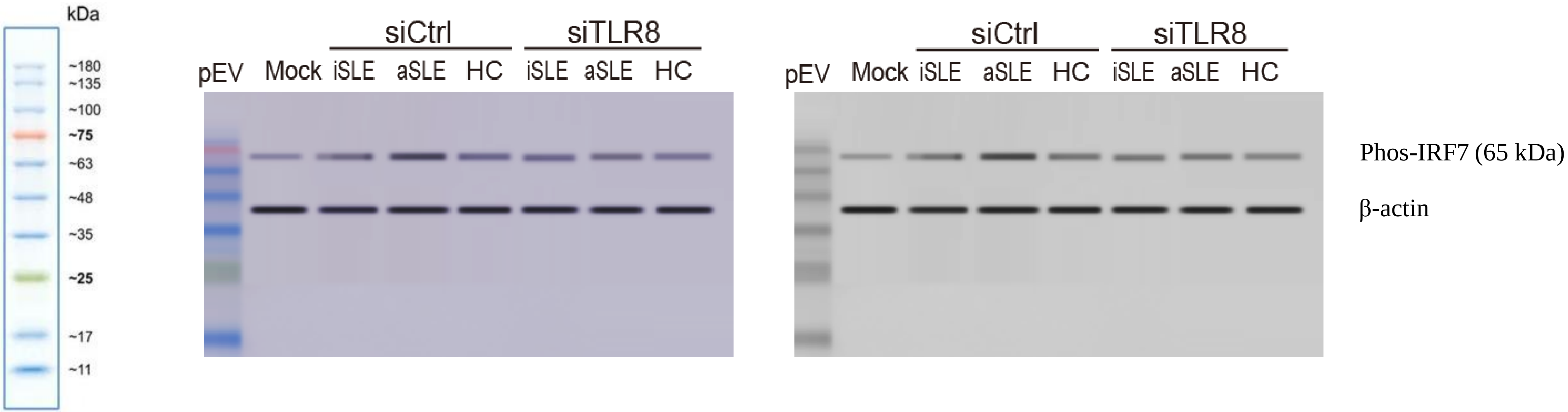

t-IRF7 (65 kDa)

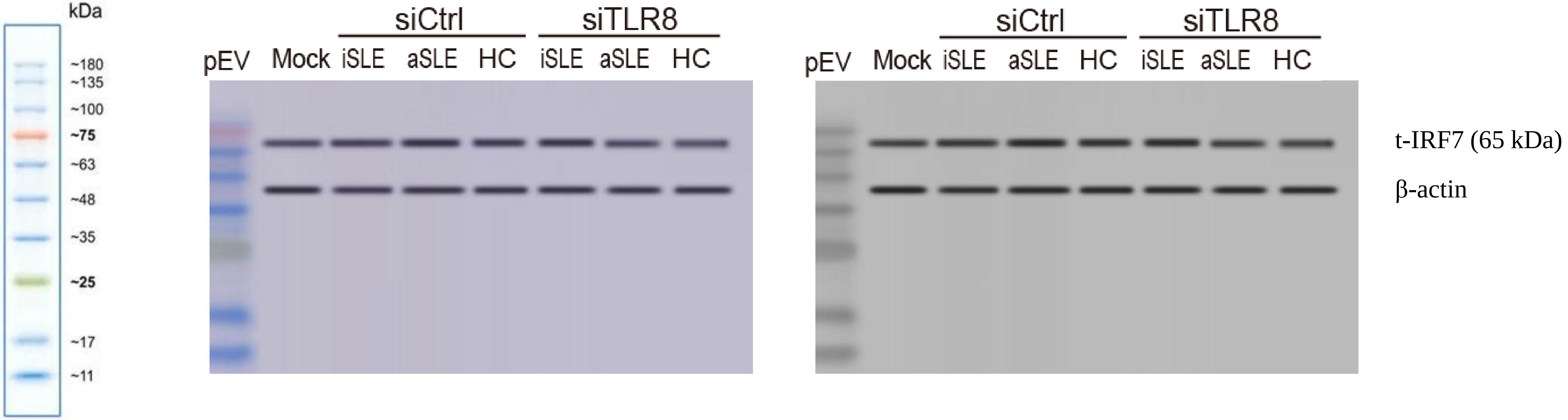
kDa
~180
~135
~100
~75
~63
~48
~35
~25
~17
~11
siCtrl
siTLR8
pEV
Mock
iSLE
aSLE
HC
iSLE
aSLE
HC
siCtrl
siTLR8
pEV
Mock
iSLE
aSLE
HC
iSLE
aSLE
HC
t-IRF7 (65 kDa)
β-actin

IFNα (22 kDa)

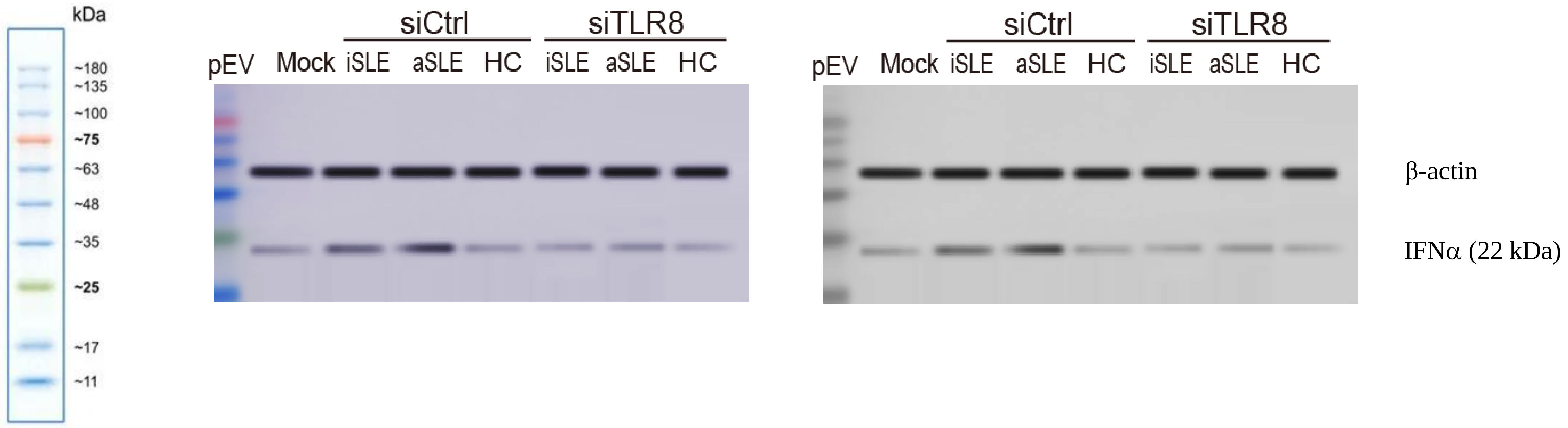
kDa
~180
~135
~100
~75
~63
~48
~35
~25
~17
~11
siCtrl
siTLR8
pEV
Mock
iSLE
aSLE
HC
iSLE
aSLE
HC
siCtrl
siTLR8
pEV
Mock
iSLE
aSLE
HC
iSLE
aSLE
HC
β-actin
IFNα (22 kDa)

Fig. 4G

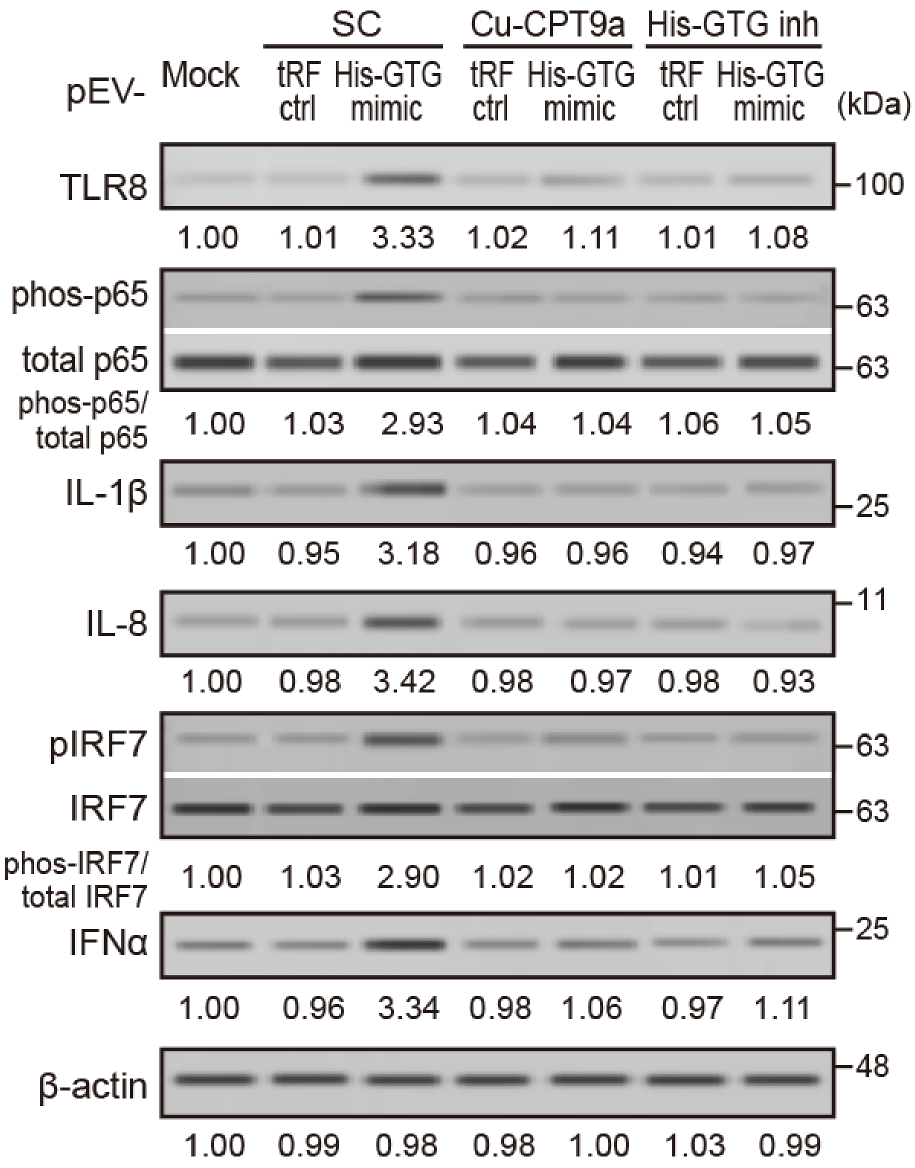
SC
Cu-CPT9a
His-GTG inh
pEV-
Mock
tRF ctrl
His-GTG mimic
tRF ctrl
His-GTG mimic
tRF ctrl
His-GTG mimic
(kDa)
TLR8
100
1.00 1.01 3.33 1.02 1.11 1.01 1.08
phos-p65
63
total p65
63
phos-p65/ total p65
1.00 1.03 2.93 1.04 1.04 1.06 1.05
IL-1β
25
1.00 0.95 3.18 0.96 0.96 0.94 0.97
IL-8
11
1.00 0.98 3.42 0.98 0.97 0.98 0.93
pIRF7
63
IRF7
63
phos-IRF7/ total IRF7
1.00 1.03 2.90 1.02 1.02 1.01 1.05
IFNα
25
1.00 0.96 3.34 0.98 1.06 0.97 1.11
β-actin
48
1.00 0.99 0.98 0.98 1.00 1.03 0.99

TLR8 (110 kDa)

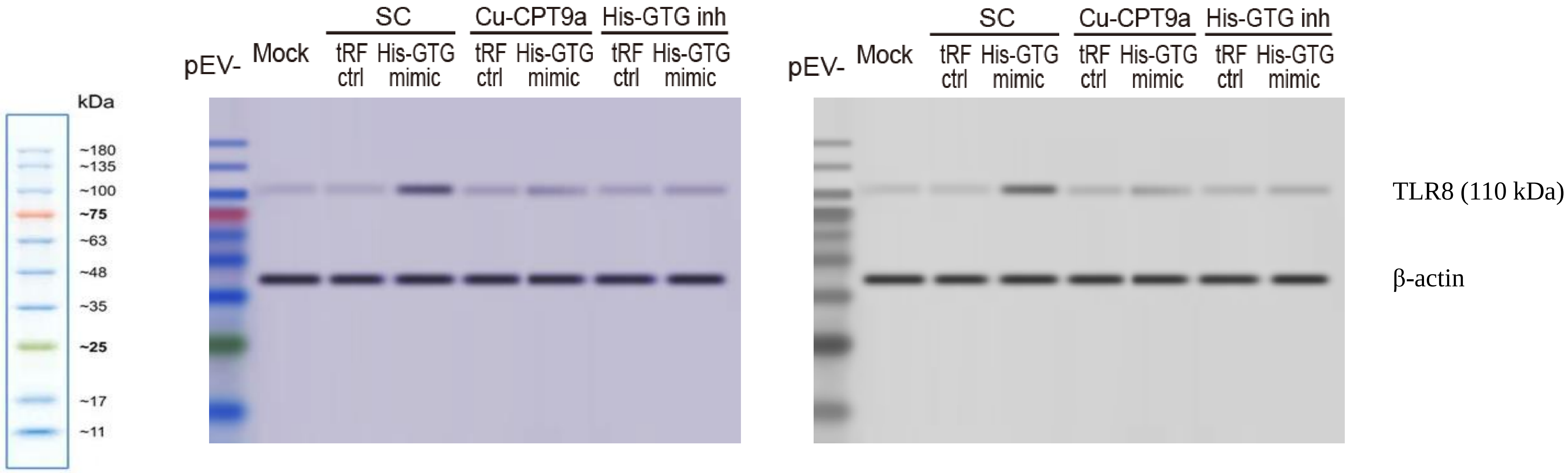
kDa
~180
~135
~100
~75
~63
~48
~35
~25
~17
~11
SC
Cu-CPT9a
His-GTG inh
pEV-
Mock
tRF ctrl
His-GTG mimic
tRF ctrl
His-GTG mimic
tRF ctrl
His-GTG mimic
SC
Cu-CPT9a
His-GTG inh
pEV-
Mock
tRF ctrl
His-GTG mimic
tRF ctrl
His-GTG mimic
tRF ctrl
His-GTG mimic
TLR8 (110 kDa)
β-actin

Phos-p65 (65 kDa)

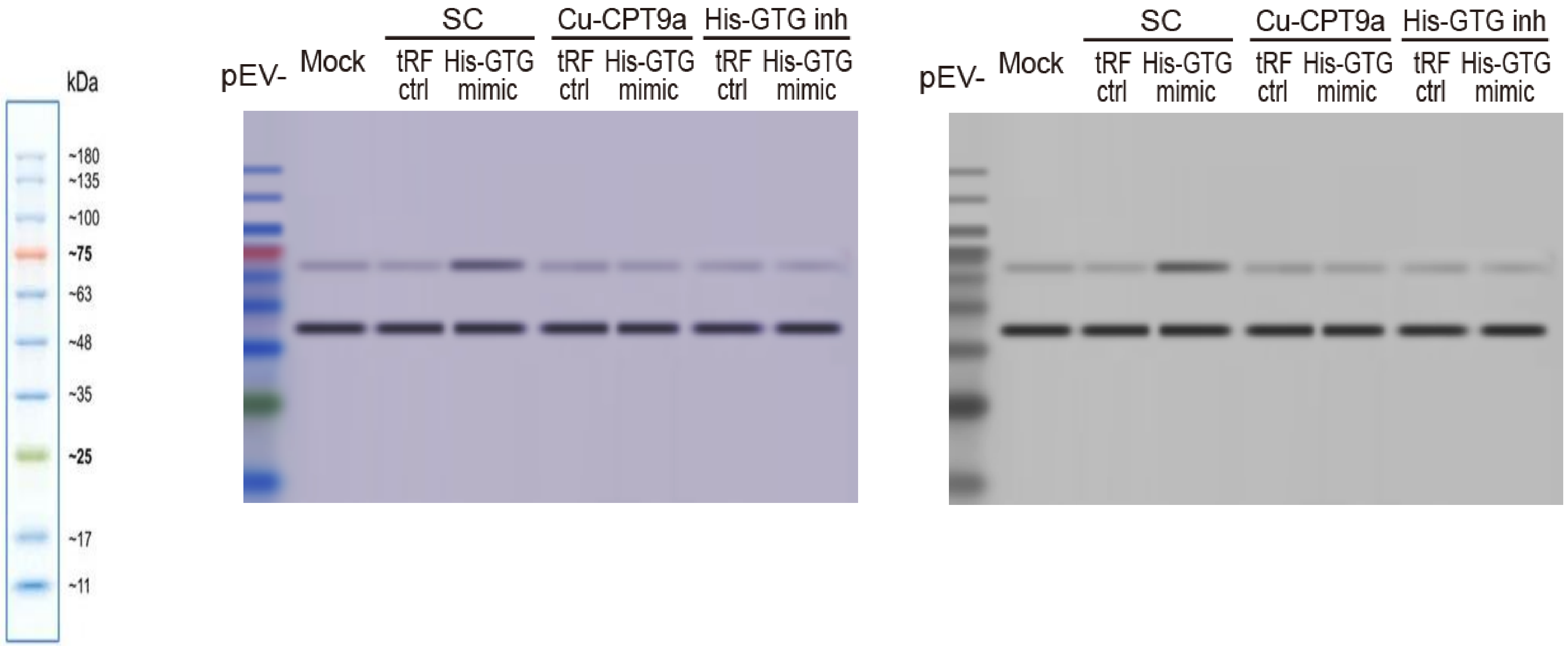

Phos-p65 (65 kDa)

β-actin

t-p65 (65 kDa)

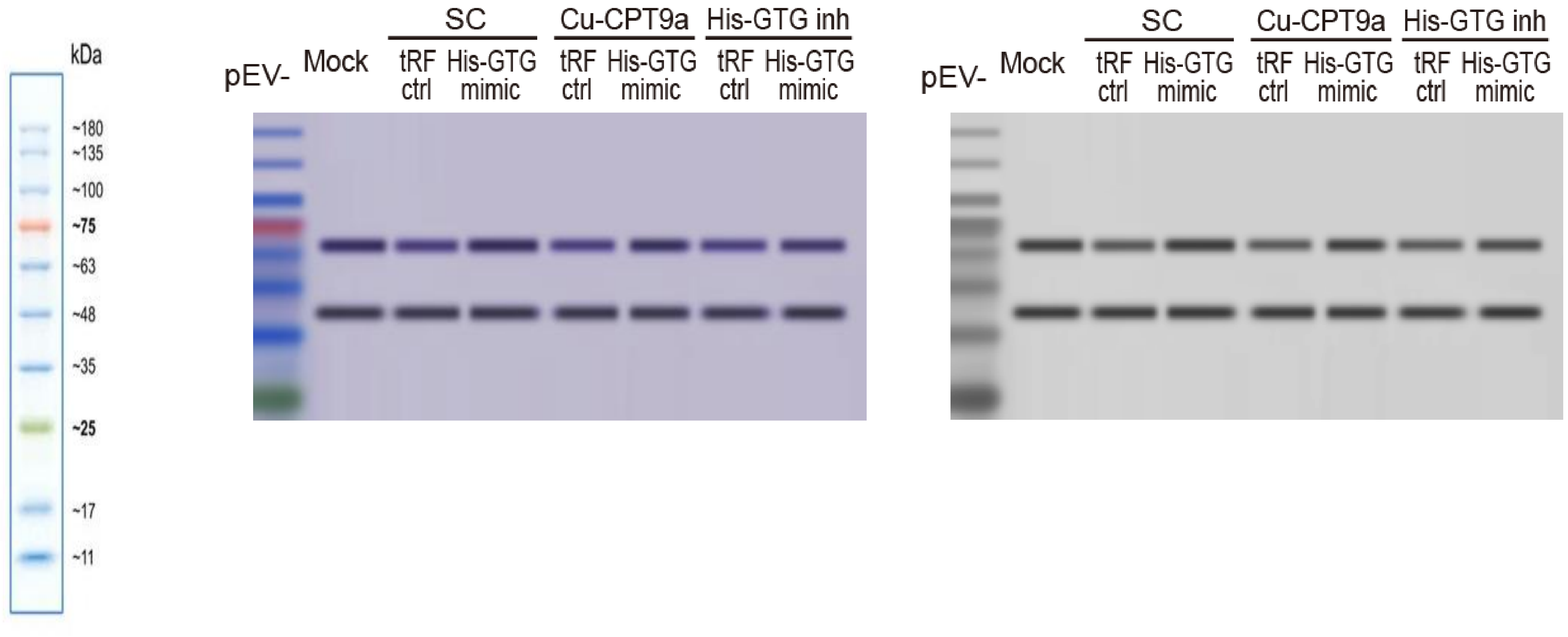

t-p65 (65 kDa)

β-actin

IL-1β (31 kDa)

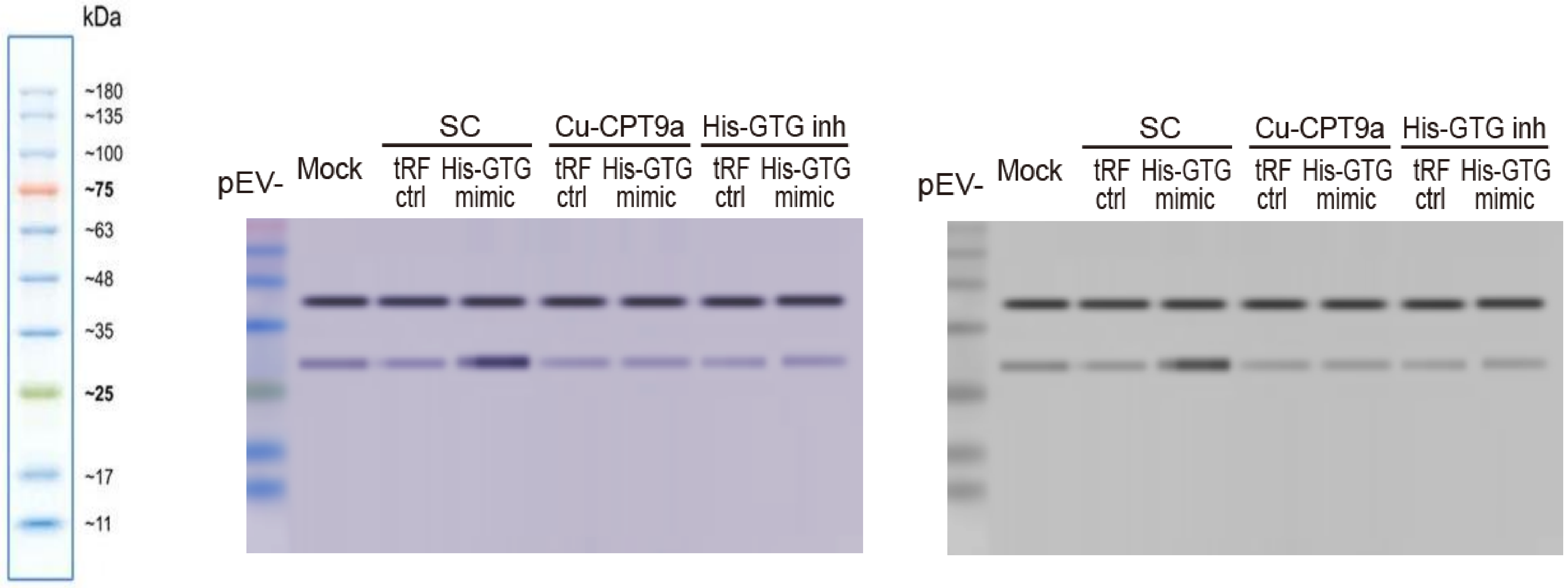

β-actin

IL-1β (31 kDa)

IL-8 (8 kDa)

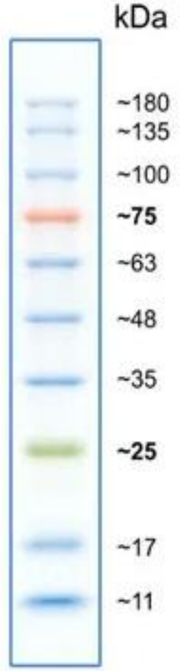

kDa
~180
~135
~100
~75
~63
~48
~35
~25
~17
~11

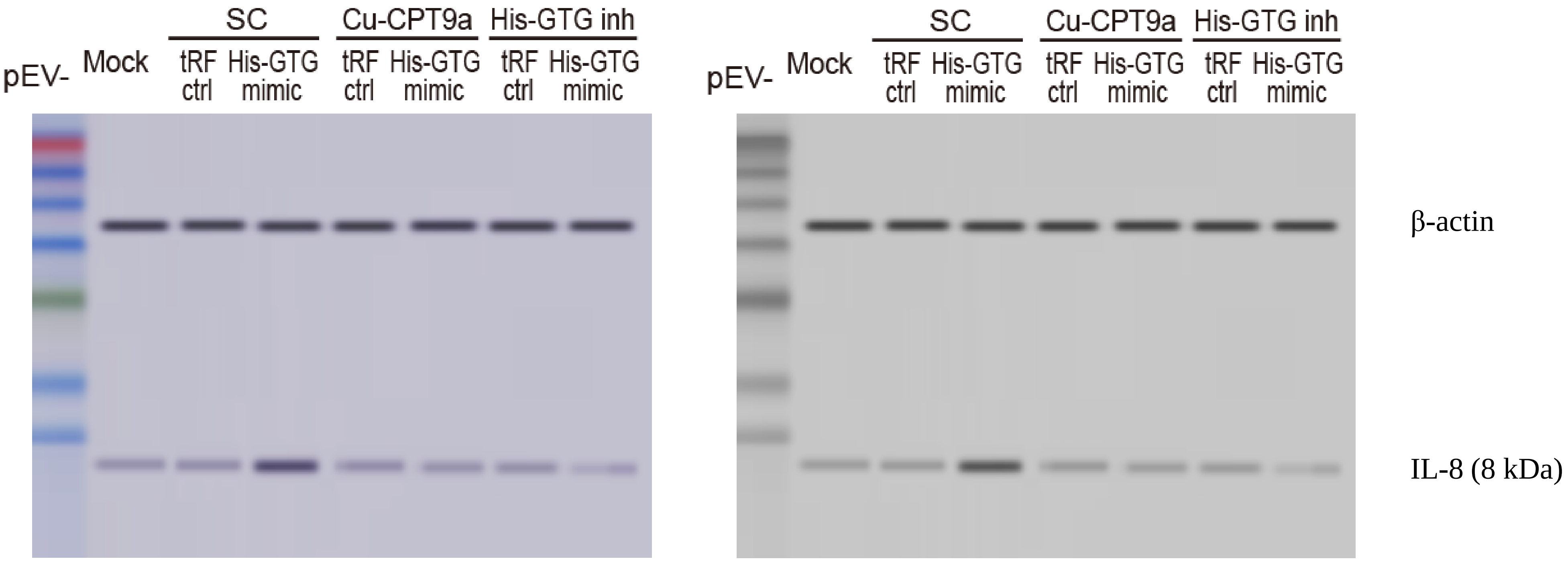

SC
Cu-CPT9a
His-GTG inh
pEV-
Mock
tRF ctrl
His-GTG mimic
tRF ctrl
His-GTG mimic
tRF ctrl
His-GTG mimic
SC
Cu-CPT9a
His-GTG inh
pEV-
Mock
tRF ctrl
His-GTG mimic
tRF ctrl
His-GTG mimic
tRF ctrl
His-GTG mimic
β-actin
IL-8 (8 kDa)

Phos-IRF7 (65 kDa)

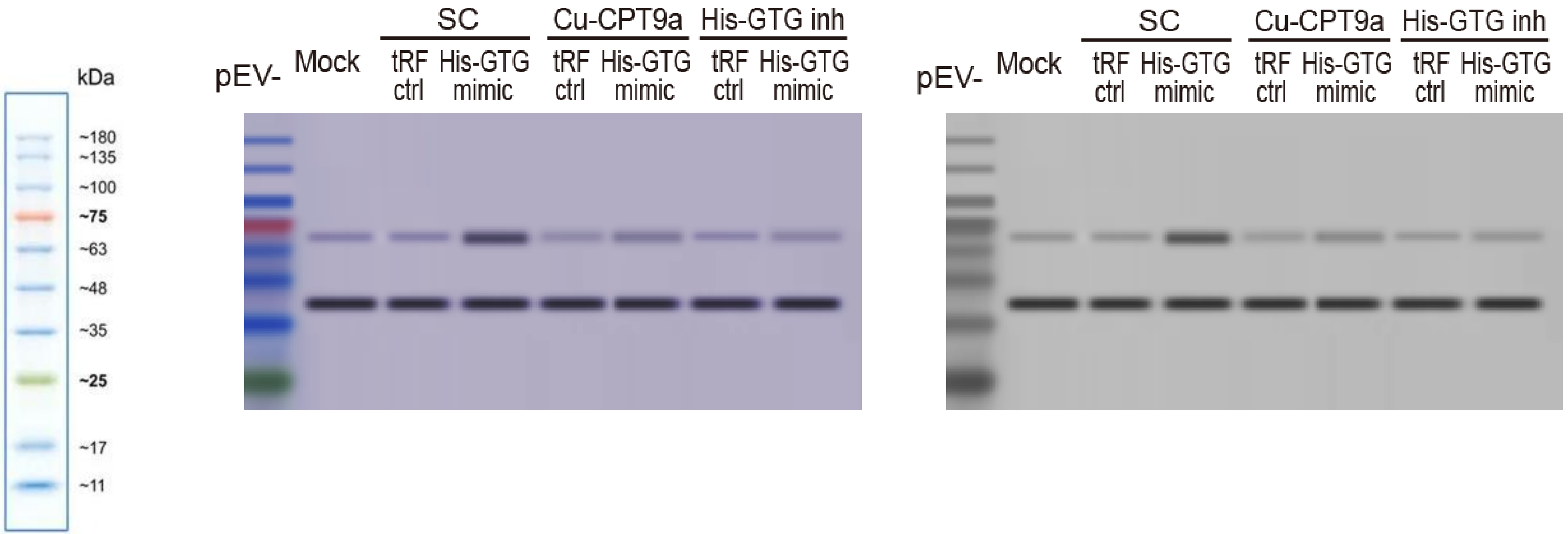

Phos-IRF7 (65 kDa)

β-actin

t-IRF7 (65 kDa)

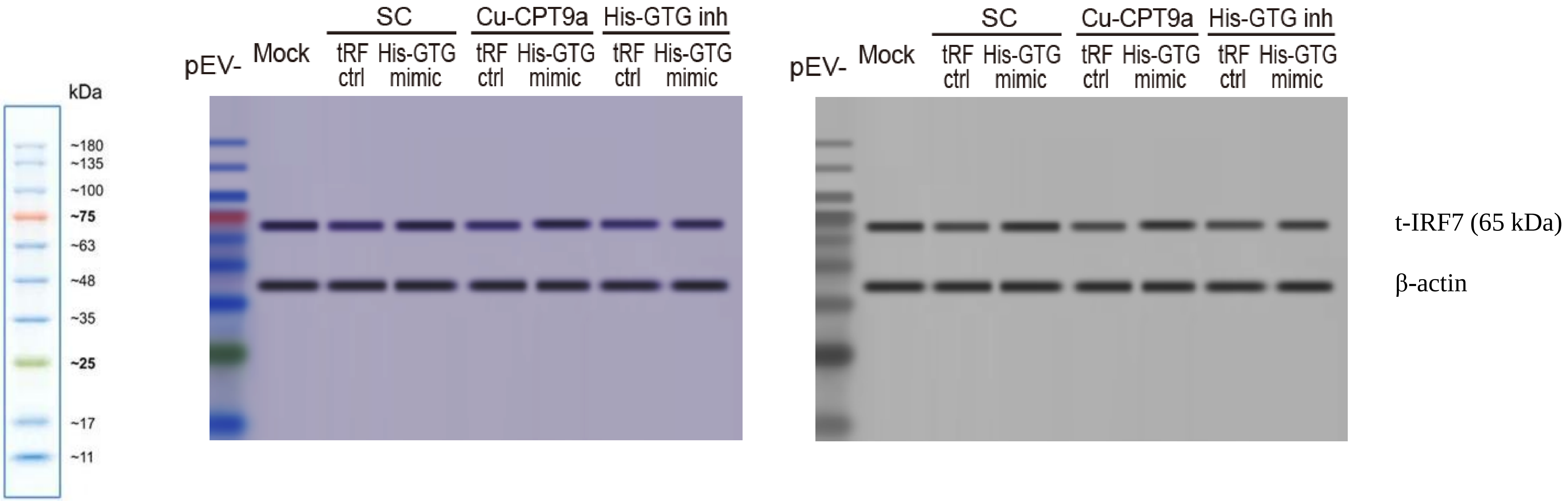
kDa
~180
~135
~100
~75
~63
~48
~35
~25
~17
~11
pEV-
Mock
SC
tRF ctrl
His-GTG mimic
Cu-CPT9a
tRF ctrl
His-GTG mimic
His-GTG inh
tRF ctrl
His-GTG mimic
pEV-
Mock
SC
tRF ctrl
His-GTG mimic
Cu-CPT9a
tRF ctrl
His-GTG mimic
His-GTG inh
tRF ctrl
His-GTG mimic
t-IRF7 (65 kDa)
β-actin

IFNα (22 kDa)

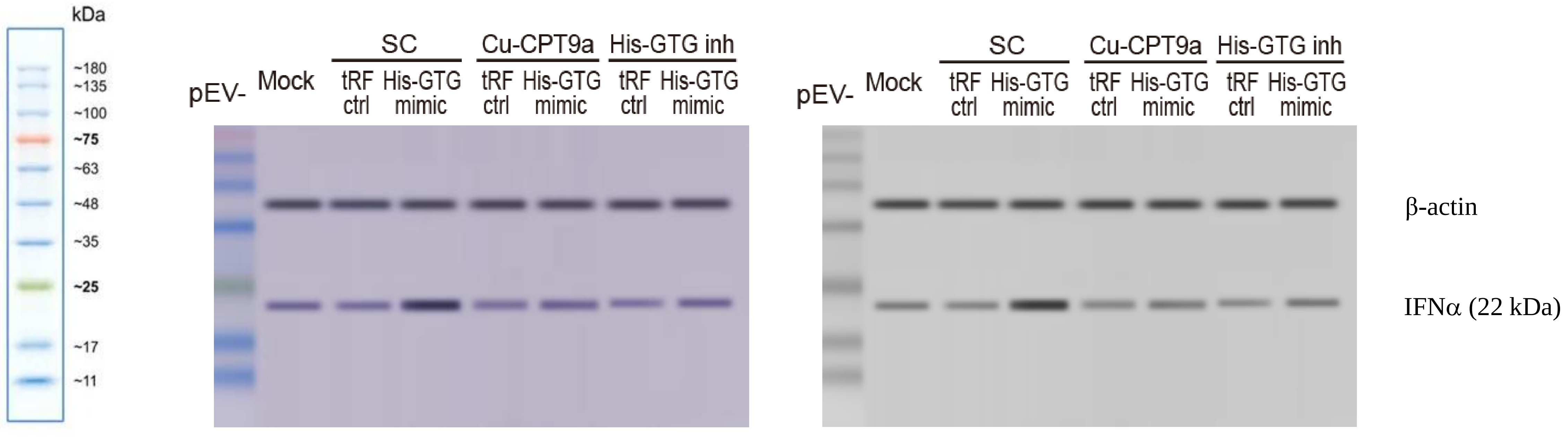
kDa
~180
~135
~100
~75
~63
~48
~35
~25
~17
~11
SC
Cu-CPT9a
His-GTG inh
pEV-
Mock
tRF ctrl
His-GTG mimic
tRF ctrl
His-GTG mimic
tRF ctrl
His-GTG mimic
SC
Cu-CPT9a
His-GTG inh
pEV-
Mock
tRF ctrl
His-GTG mimic
tRF ctrl
His-GTG mimic
tRF ctrl
His-GTG mimic
β-actin
IFNα (22 kDa)

Fig. 4I

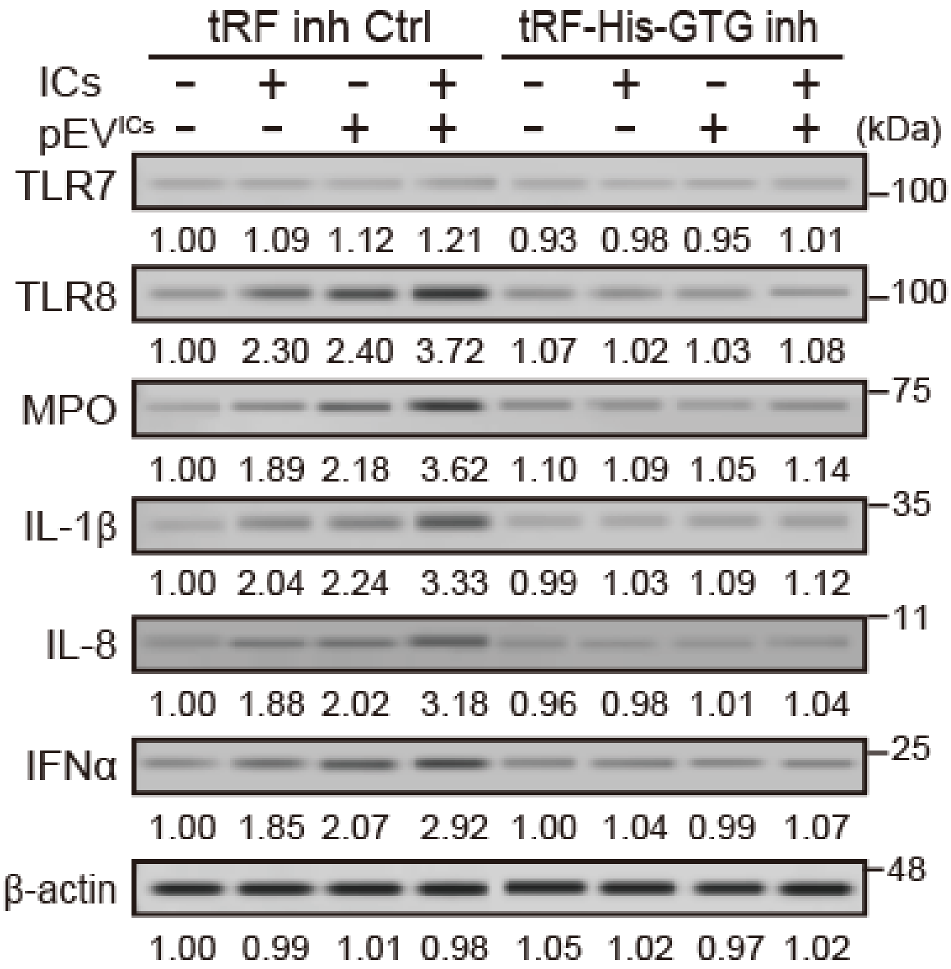
tRF inh Ctrl
tRF-His-GTG inh
ICs - + - + - + - +
pEV^ICs - - + + - - + +
(kDa)
TLR7
100
1.00 1.09 1.12 1.21 0.93 0.98 0.95 1.01
TLR8
100
1.00 2.30 2.40 3.72 1.07 1.02 1.03 1.08
MPO
75
1.00 1.89 2.18 3.62 1.10 1.09 1.05 1.14
IL-1β
35
1.00 2.04 2.24 3.33 0.99 1.03 1.09 1.12
IL-8
11
1.00 1.88 2.02 3.18 0.96 0.98 1.01 1.04
IFNα
25
1.00 1.85 2.07 2.92 1.00 1.04 0.99 1.07
β-actin
48
1.00 0.99 1.01 0.98 1.05 1.02 0.97 1.02

TLR7 (121 kDa)

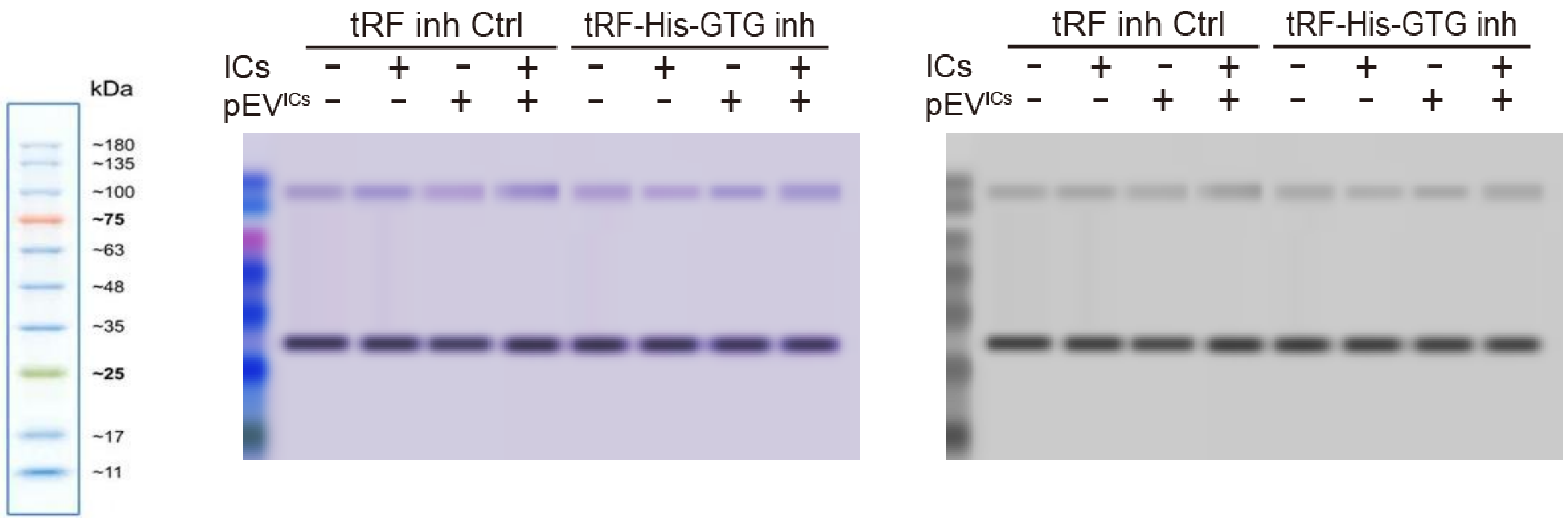

TLR7 (121 kDa)

β-actin

TLR8 (110 kDa)

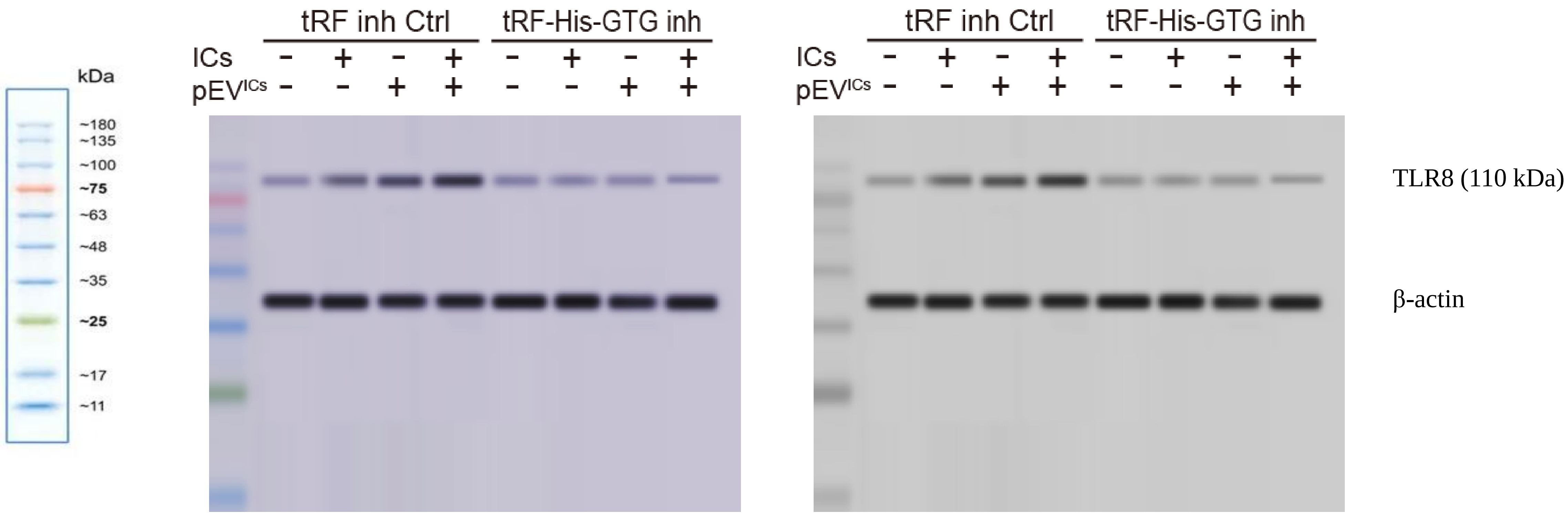
kDa
~180
~135
~100
~75
~63
~48
~35
~25
~17
~11
tRF inh Ctrl
tRF-His-GTG inh
ICs - + - + - + - +
pEV^ICs - - + + - - + +
tRF inh Ctrl
tRF-His-GTG inh
ICs - + - + - + - +
pEV^ICs - - + + - - + +
TLR8 (110 kDa)
β-actin

MPO (72 kDa)

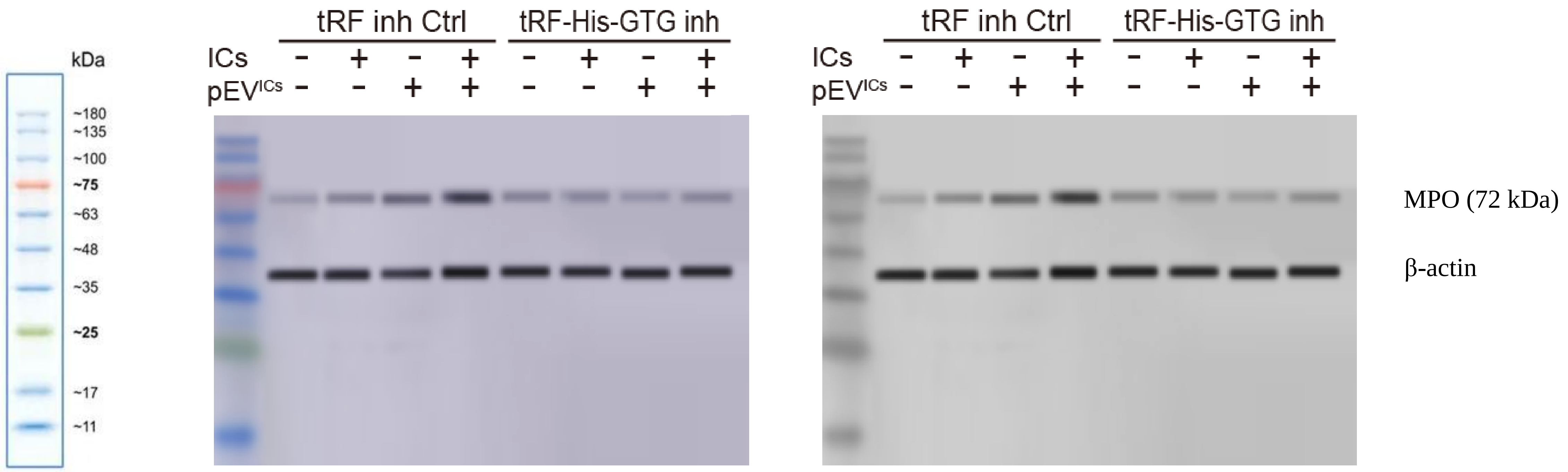
kDa
~180
~135
~100
~75
~63
~48
~35
~25
~17
~11
tRF inh Ctrl
tRF-His-GTG inh
ICs - + - + - + - +
pEVICs - - + + - - + +
tRF inh Ctrl
tRF-His-GTG inh
ICs - + - + - + - +
pEVICs - - + + - - + +
MPO (72 kDa)
β-actin

IL-1β (31 kDa)

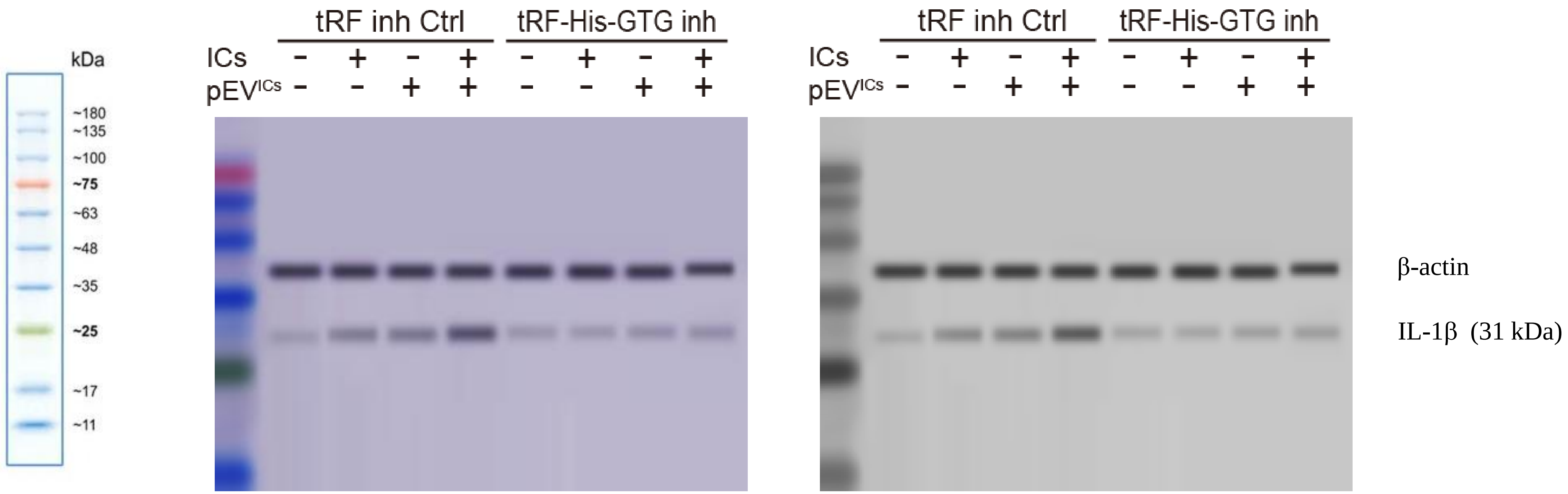

kDa
~180
~135
~100
~75
~63
~48
~35
~25
~17
~11
tRF inh Ctrl
tRF-His-GTG inh
ICs - + - + - + - +
pEV^ICs - - + + - - + +
β-actin
IL-1β (31 kDa)

IL-8 (8 kDa)

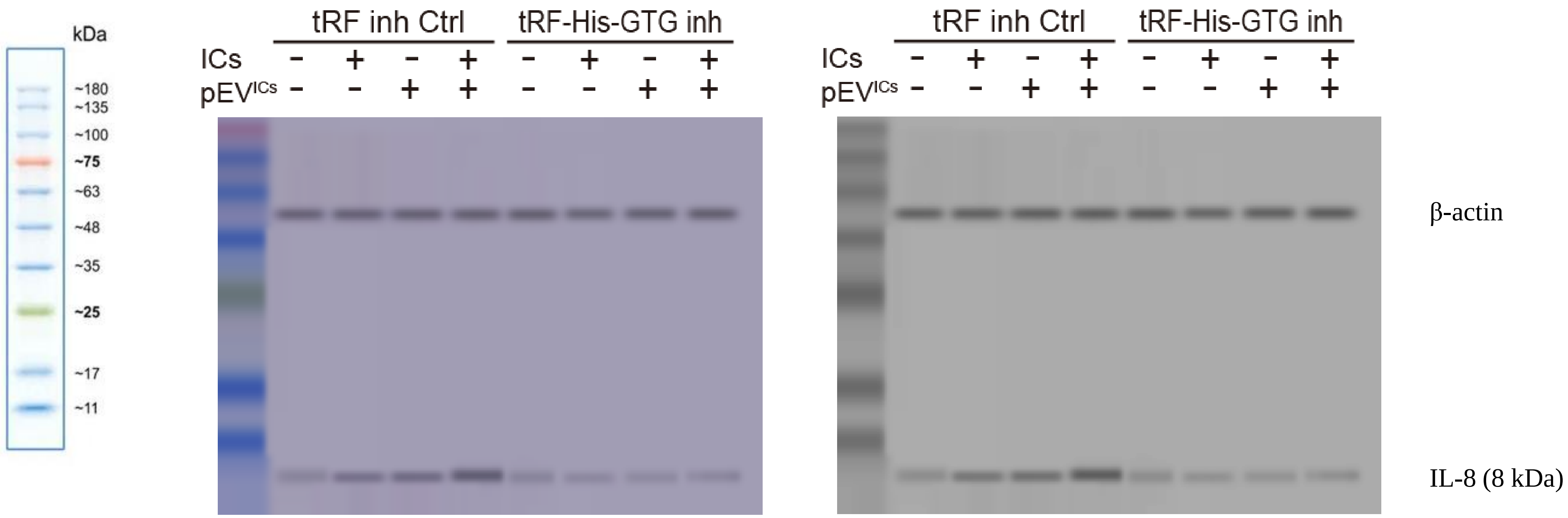
kDa
~180
~135
~100
~75
~63
~48
~35
~25
~17
~11
tRF inh Ctrl
tRF-His-GTG inh
ICs - + - + - + - +
pEVICs - - + + - - + +
tRF inh Ctrl
tRF-His-GTG inh
ICs - + - + - + - +
pEVICs - - + + - - + +
β-actin
IL-8 (8 kDa)

IFNα (22 kDa)

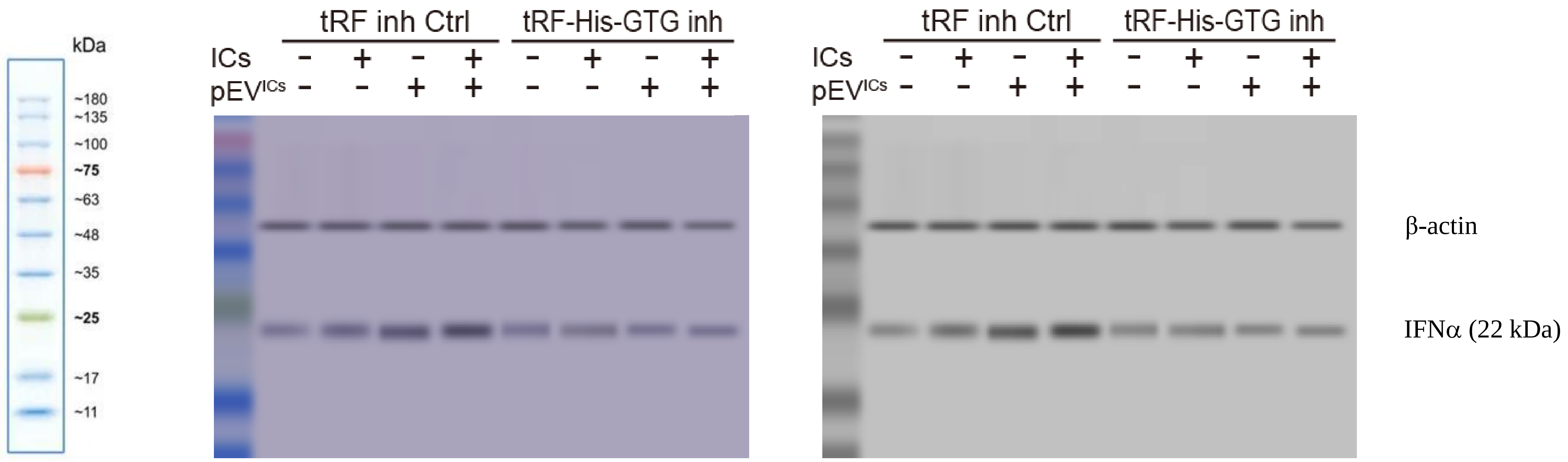
kDa
~180
~135
~100
~75
~63
~48
~35
~25
~17
~11
tRF inh Ctrl
tRF-His-GTG inh
ICs - + - + - + - +
pEV^ICs - - + + - - + +
tRF inh Ctrl
tRF-His-GTG inh
ICs - + - + - + - +
pEV^ICs - - + + - - + +
β-actin
IFNα (22 kDa)

Fig. S2

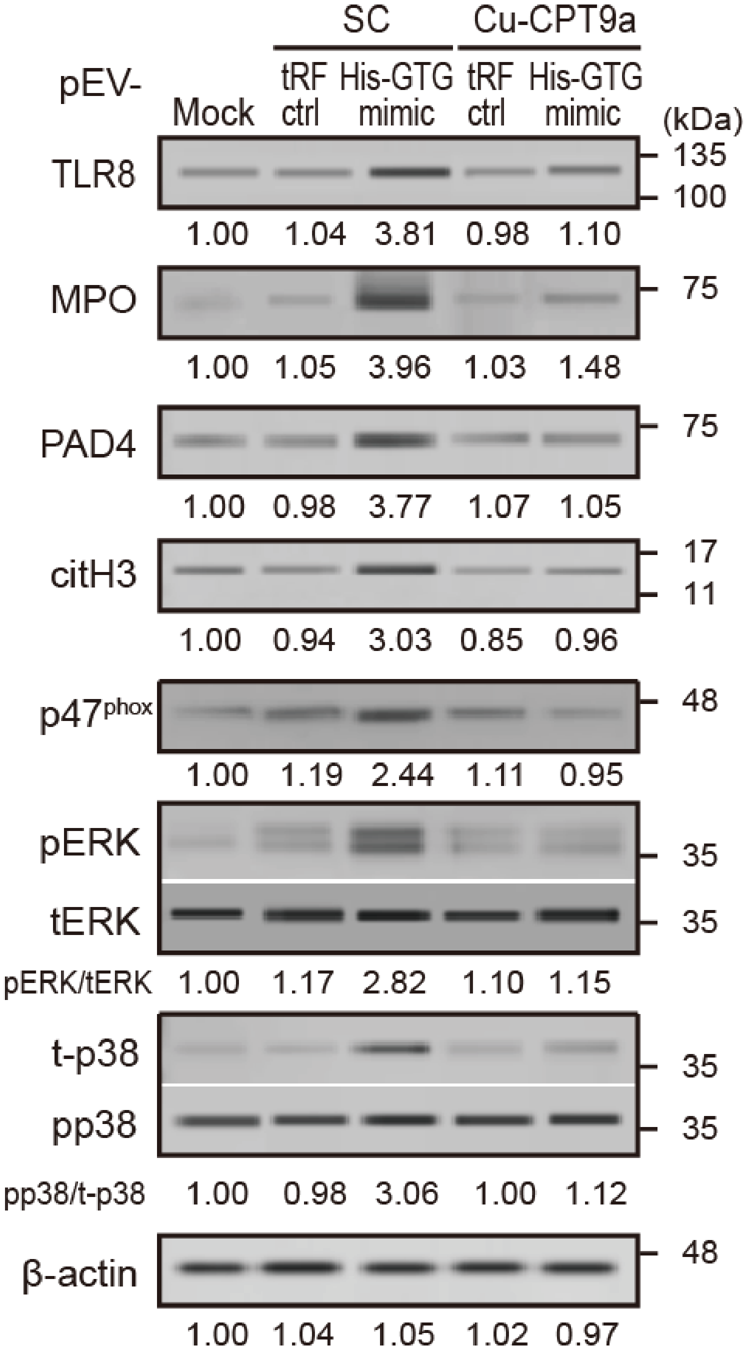
SC
Cu-CPT9a
pEV-
Mock
tRF ctrl
His-GTG mimic
tRF ctrl
His-GTG mimic
(kDa)
TLR8
135
100
1.00 1.04 3.81 0.98 1.10
MPO
75
1.00 1.05 3.96 1.03 1.48
PAD4
75
1.00 0.98 3.77 1.07 1.05
citH3
17
11
1.00 0.94 3.03 0.85 0.96
p47phox
48
1.00 1.19 2.44 1.11 0.95
pERK
35
tERK
35
pERK/tERK 1.00 1.17 2.82 1.10 1.15
t-p38
35
pp38
35
pp38/t-p38 1.00 0.98 3.06 1.00 1.12
β-actin
48
1.00 1.04 1.05 1.02 0.97

TLR8 (110 kDa)

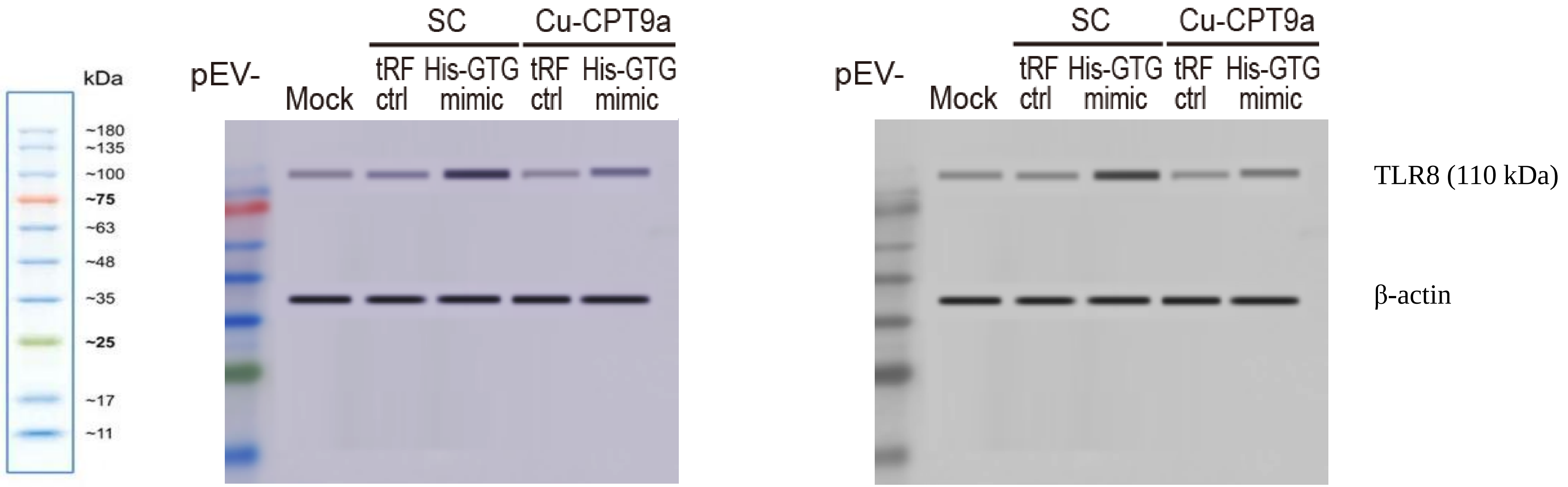
kDa
~180
~135
~100
~75
~63
~48
~35
~25
~17
~11
pEV-
Mock
SC
tRF ctrl
His-GTG mimic
Cu-CPT9a
tRF ctrl
His-GTG mimic
pEV-
Mock
SC
tRF ctrl
His-GTG mimic
Cu-CPT9a
tRF ctrl
His-GTG mimic
TLR8 (110 kDa)
β-actin

MPO (72 kDa)

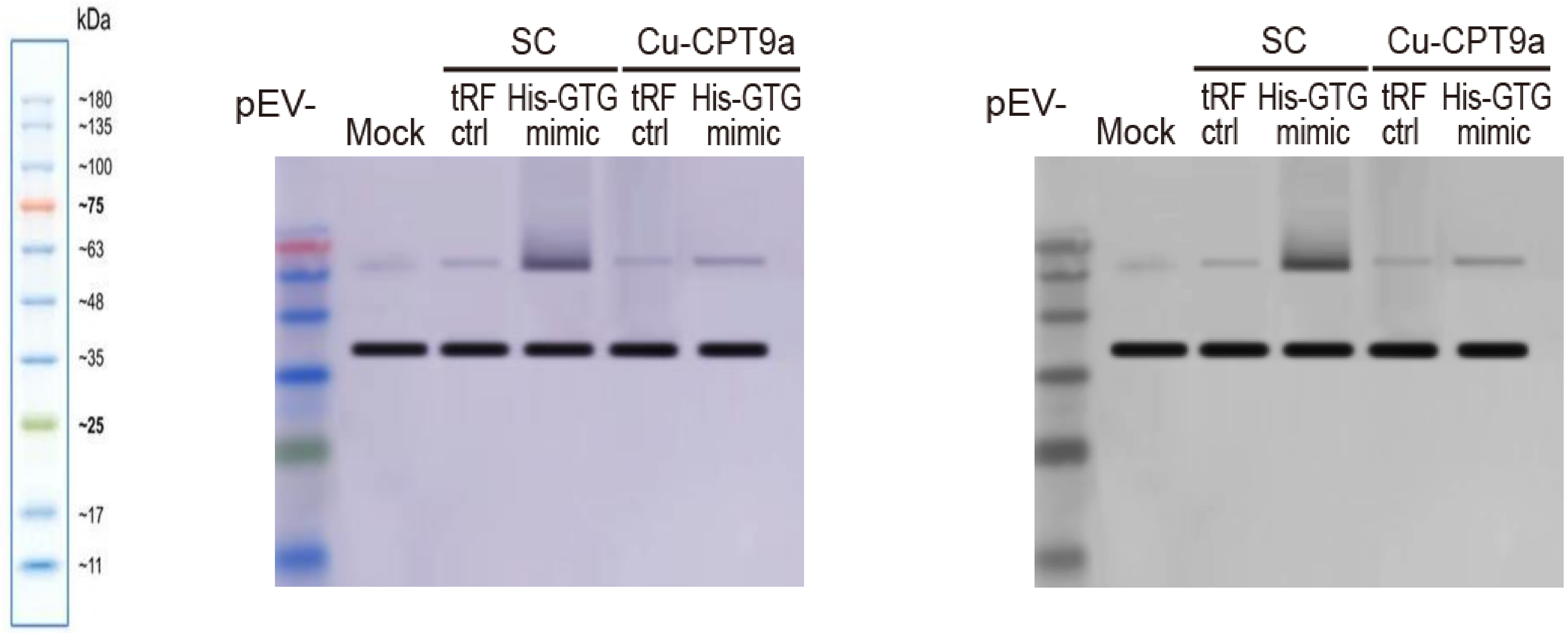

MPO

β-actin

PAD4(72 kDa)

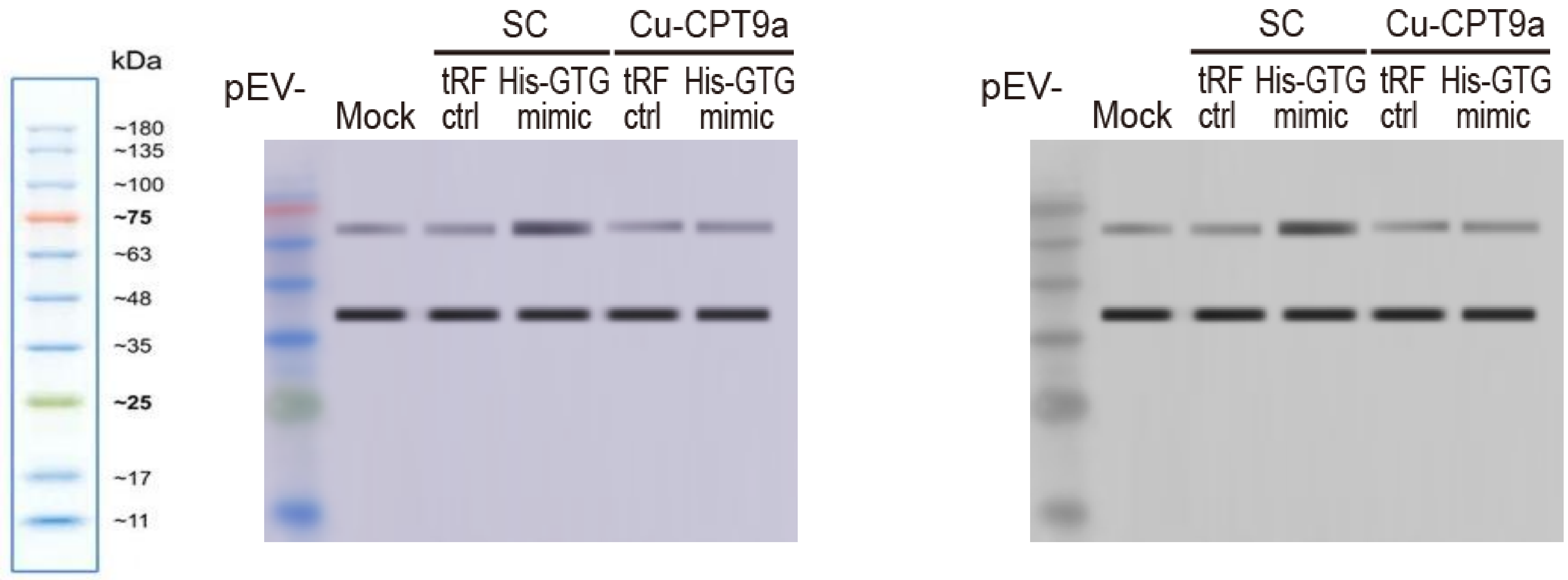

PAD4(72 kDa)

β–actin

citH3(14~17 kDa)

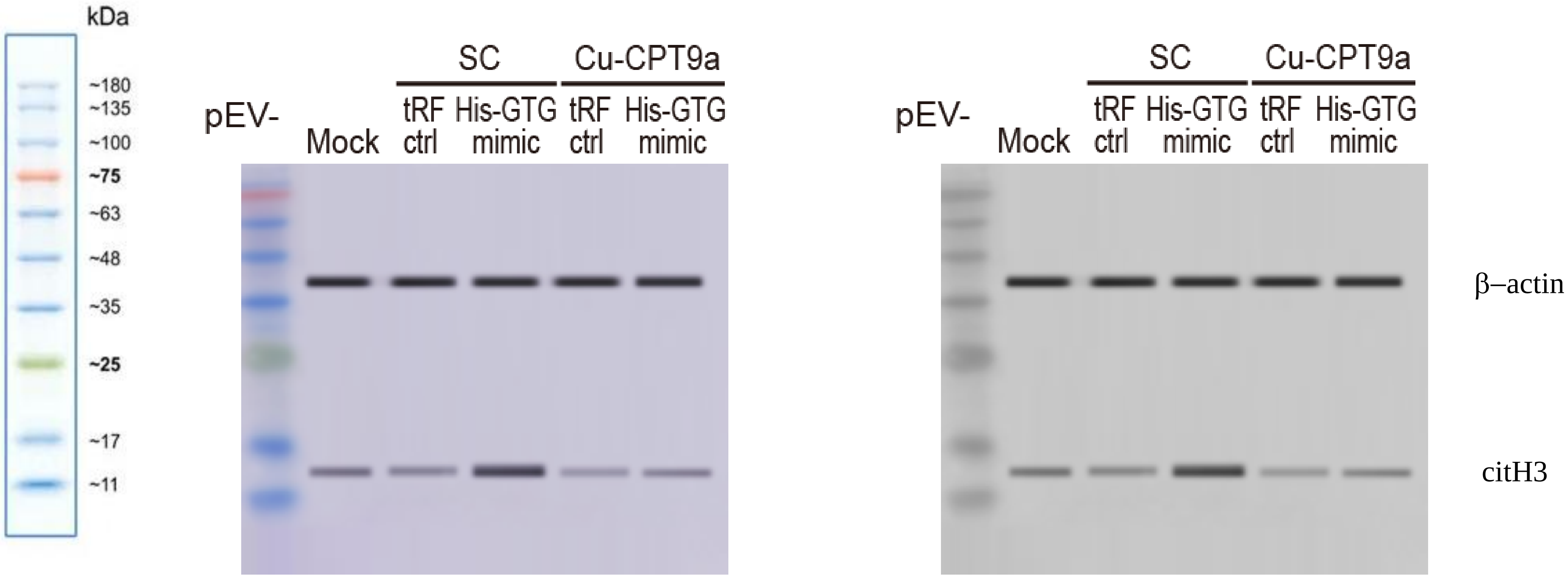

p47 phox (47 kDa)

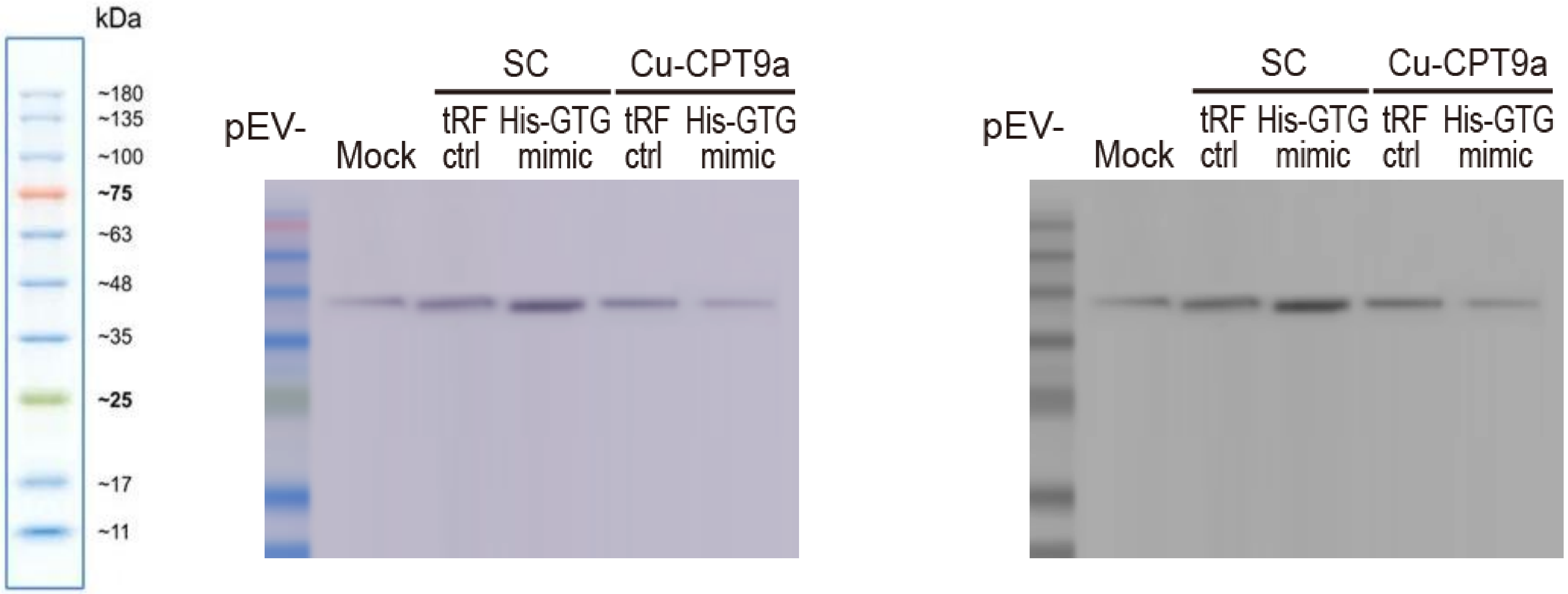

p47 phox (47 kDa)

pERK (42, 44 kDa)

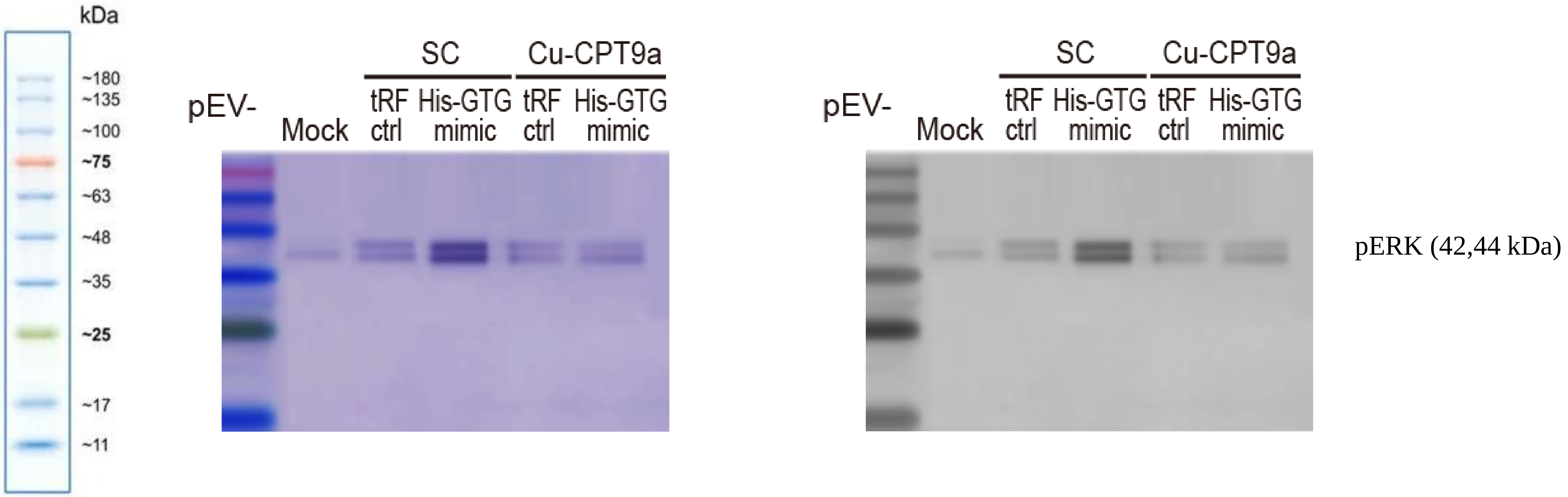

tERK (44 kDa)

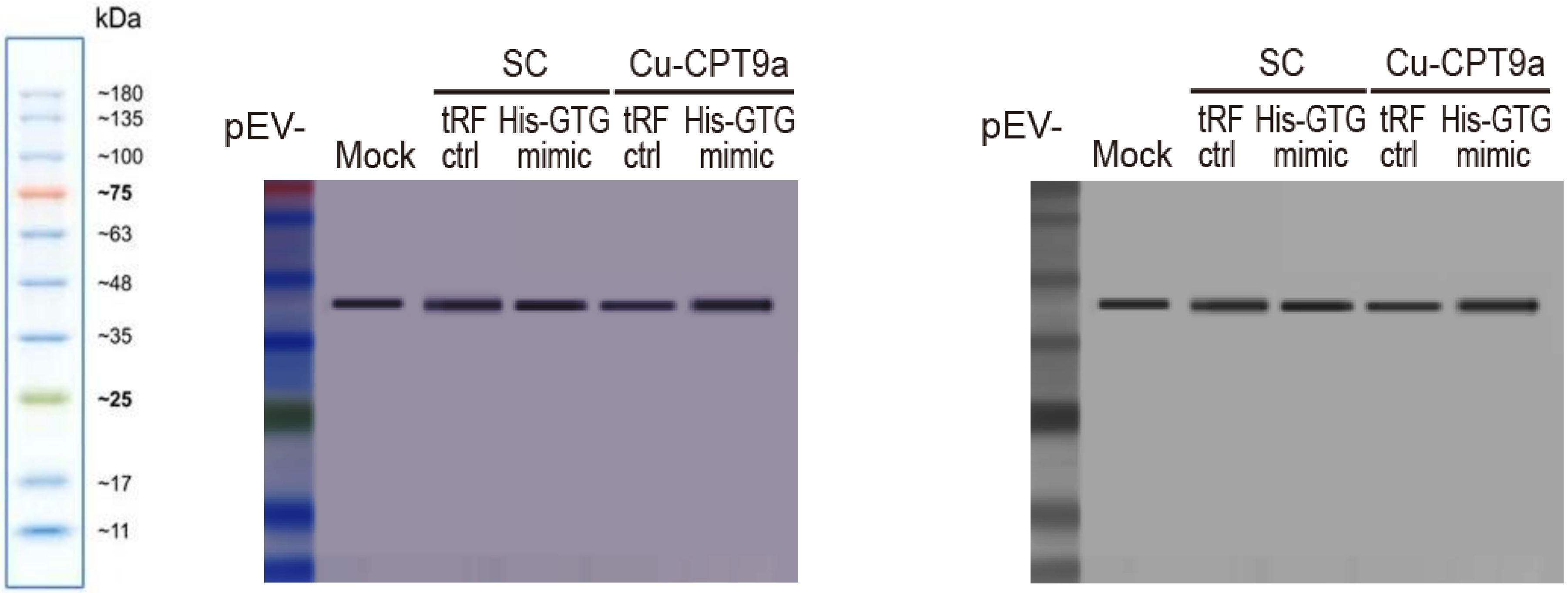

tERK (44 kDa)

Phos-p38 (43 kDa)

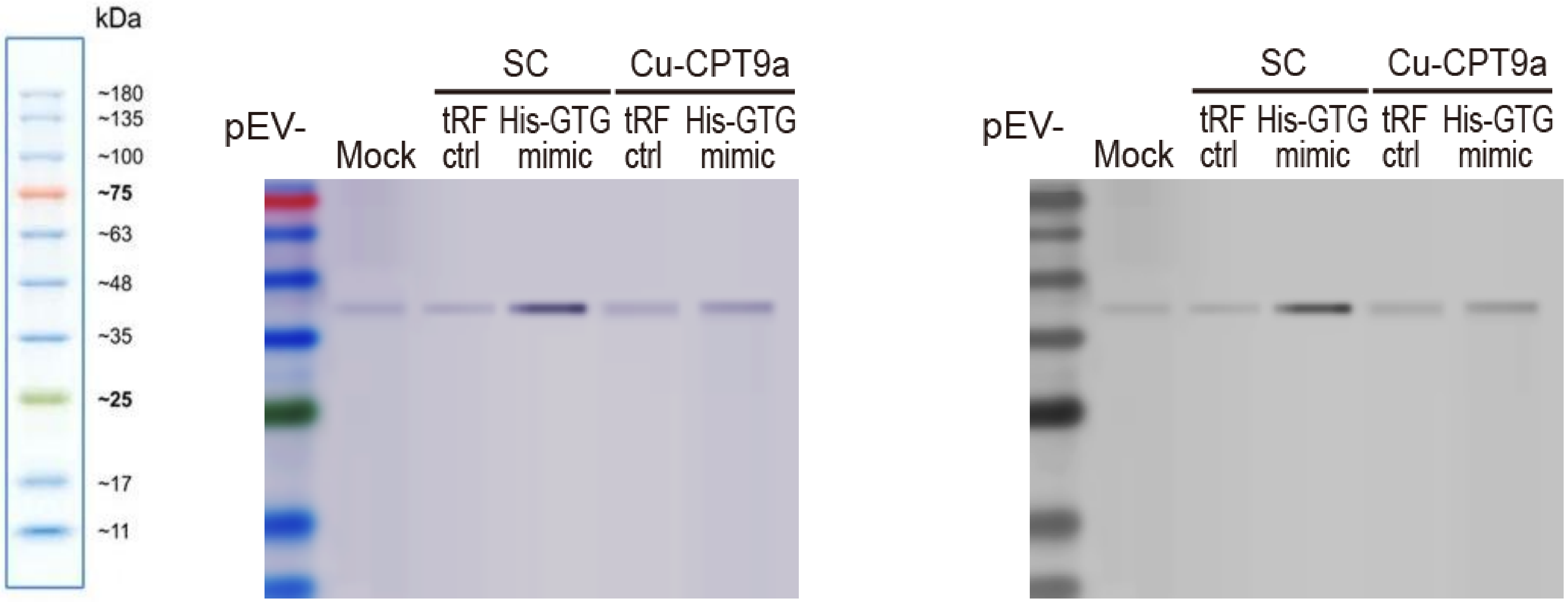

Phos-p38 (43 kDa)

t-p38 (40 kDa)

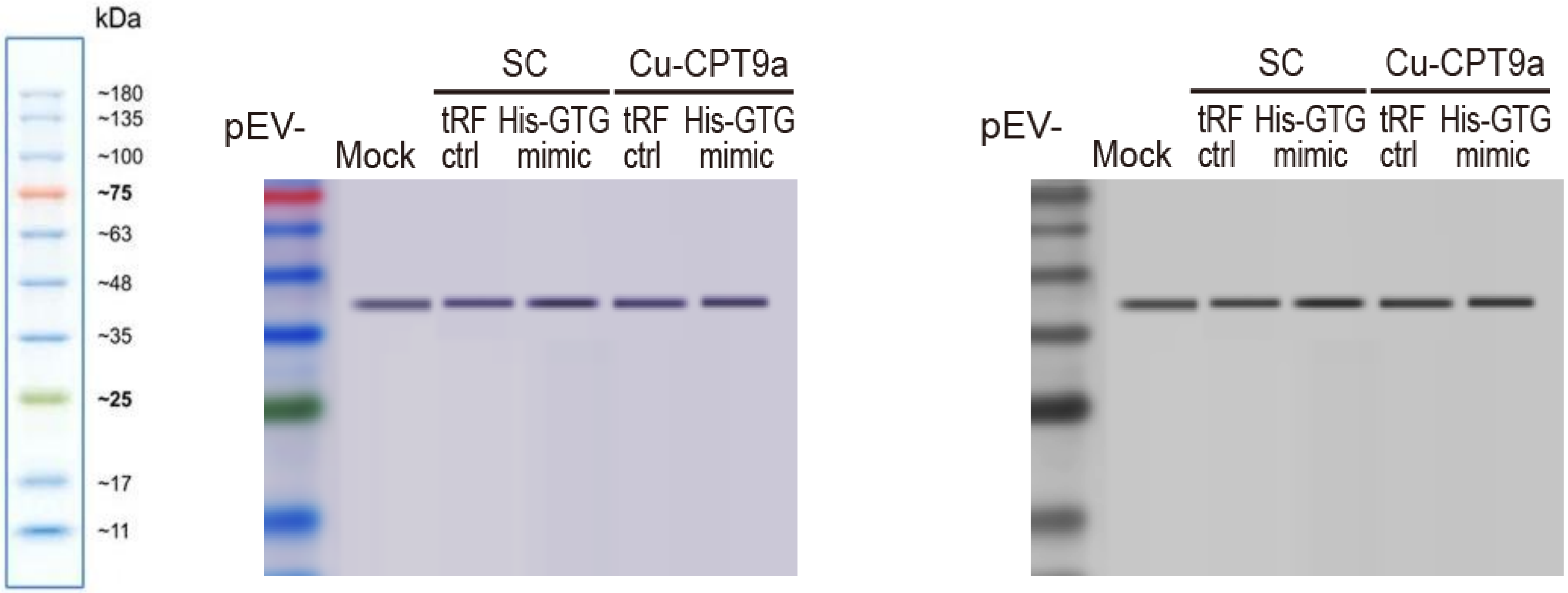

t-p38 (40 kDa)
